# Mesoporous Silica Nanoparticles‐Based Formulations for Enhanced Oral Delivery of Peptide Drugs: A Case Study on Insulin

**DOI:** 10.1002/smll.202513347

**Published:** 2026-03-22

**Authors:** Claudia Iriarte‐Mesa, Estelle Juère, Andrea Bileck, Thomas Kremsmayr, Michael L. Goodson, Allison Ehrlich, Adnan Hodžić, Martin Kunert, Christopher Gerner, Hanspeter Kählig, Doris Marko, Markus Muttenthaler, David Berry, Giorgia Del Favero, Freddy Kleitz

**Affiliations:** ^1^ Department of Functional Materials and Catalysis Faculty of Chemistry University of Vienna Vienna Austria; ^2^ Vienna Doctoral School in Chemistry (DoSChem) University of Vienna Vienna Austria; ^3^ Department of Analytical Chemistry Faculty of Chemistry University of Vienna Vienna Austria; ^4^ Joint Metabolome Facility University of Vienna and Medical University of Vienna Vienna Austria; ^5^ Institute of Biological Chemistry Faculty of Chemistry University of Vienna Vienna Austria; ^6^ Department of Environmental Toxicology College of Agriculture and Environmental Science University of California Davis California USA; ^7^ Department of Anatomy Physiology and Cell Biology School of Veterinary Medicine University of California Davis California USA; ^8^ Centre For Microbiology and Environmental Systems Science Department of Microbiology and Ecosystem Science Division of Microbial Ecology University of Vienna Vienna Austria; ^9^ Department of Organic Chemistry Faculty of Chemistry University of Vienna Vienna Austria; ^10^ Department of Food Chemistry and Toxicology Faculty of Chemistry University of Vienna Vienna Austria; ^11^ Institute for Molecular Bioscience The University of Queensland Brisbane Queensland Australia; ^12^ Joint Microbiome Facility of the Medical University of Vienna and the University of Vienna Vienna Austria; ^13^ Core Facility Multimodal Imaging Faculty of Chemistry University of Vienna Vienna Austria

**Keywords:** gastrointestinal degradation, insulin‐responsive signaling, insulin transport, mesoporous silica nanoparticles, oral peptide drug delivery

## Abstract

Peptide drugs have revolutionized modern medicine owing to their high potency, selectivity, and excellent tolerability. However, oral delivery remains limited, and most peptide drugs are administered parenterally due to their inherent instability to proteolytic digestion and poor ability to cross gastrointestinal barriers, which hinders efficient absorption into the bloodstream. This study presents a multifunctional oral delivery system based on mesoporous silica nanoparticles (MSN) customized for insulin administration. Insulin‐loaded MSN were co‐formulated with succinylated β‐lactoglobulin to produce pH‐responsive tablets that limited premature gastric release (≤13% after 2 h at pH 1.2) and protected insulin from enzymatic degradation, while enabling controlled intestinal release (up to 88%–98% at pH 7.4). Surface functionalization with polyethylene glycol and phosphonate moieties improved colloidal stability and increased insulin solubility by ∼2.5‐fold. The interaction of phosphonated MSN with intestinal epithelial cells further induced transient reorganization of tight junction proteins, enhancing paracellular insulin transport (26% after 24 h, compared with 13% for non‐confined insulin). Delivered insulin retained bioactivity, as demonstrated by activation of insulin‐responsive signaling pathways in vitro and reduced blood glucose levels in hyperglycemic mice. These results highlight MSN as a promising platform for oral peptide delivery with improved efficacy and patient compliance.

## Graphical abstract

This work explores the tailored functionalization of mesoporous silica nanoparticles (MSN) to improve the oral delivery of insulin, as a case study. Insulin confinement within silica mesopores increased drug solubility, and co‐formulation with succinylated β‐lactoglobulin yielded pH‐responsive tablets that limited gastric release and proteolysis, while enabling controlled intestinal delivery. MSN modulated intestinal barrier function, promoting paracellular transport of bioactive insulin.

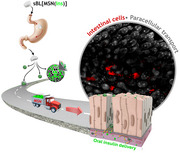

## 1 Introduction

Oral delivery is widely regarded as the gold standard for drug administration. However, for biologics—particularly peptides and proteins—this approach remains challenging [1]. Although these therapeutics exhibit high potency, target specificity, and low systemic toxicity, their intrinsically low oral bioavailability limits their administration to parenteral injection [2], which remains invasive, particularly for chronic treatments. Parental administration also entails risks of local irritation, infection, site pain, and tissue damage [3]. Therefore, the oral route has been pursued to enhance patient compliance and acceptance by offering a non‐invasive, convenient, and more suitable option for preventive and long‐term treatments [3, 4]. Advances in peptide and protein discovery, production, and modification have enhanced oral drug delivery by increasing stability against enzymatic degradation and optimizing molecular structures to facilitate membrane permeability [5, 6, 7, 8, 9]. Consequently, these engineered biomolecules have transformed the therapeutic landscape, enabling oral treatments for metabolic, hormonal, autoimmune, and inflammatory diseases [5, 6]. Clinical trials with oral peptide‐ and protein‐based therapeutics are progressively increasing, achieving unprecedented milestones in the oral administration of peptide drugs such as insulin, calcitonin, parathyroid hormone, somatostatin, and vasopressin, and even leading to the 2019 approval of oral semaglutide (Rybelsus) as the first orally administered glucagon‐like peptide‐1 (GLP‐1) receptor agonist for the treatment of type 2 diabetes [10, 11]. This achievement is largely attributed to its co‐formulation with the absorption enhancer sodium N‐(8‐[2‐hydroxybenzoyl]amino) caprylate (SNAC), which creates a localized buffering microenvironment around semaglutide. This neutralizes stomach acidity, stabilizes the peptide drug upon exposure to gastric fluids, and protects it from proteolysis [9, 12]. In addition, SNAC interacts with lipid membranes, transiently increasing their permeability, thereby facilitating the transcellular absorption of semaglutide [12]. Despite these advancements, the oral bioavailability of Rybelsus remains limited to about 1%, underscoring the need for further improvement in oral peptide delivery systems [13, 14]. Therefore, establishing safe and efficient strategies for oral administration is still crucial to expanding the range of treatable conditions and enhancing patient compliance [2]. To achieve this, drug carriers must be optimized to increase loading capacity, enable controlled release, reduce toxicity, and support cost‐effective large‐scale production [2, 15]. Furthermore, oral formulations should efficiently transit through the digestive tract, minimizing mucus trapping and peptide drug degradation while ensuring optimal intestinal absorption, high bioavailability, and effective therapeutic outcomes [5, 10].

Several peptide drugs have been used as models to test the efficacy of oral formulations [1, 4, 16]. Among them, insulin has received particular attention due to its clinical significance, large market cap, and long‐standing challenge to develop oral delivery systems for diabetes management [17, 18]. Insulin, like most peptides and proteins, is highly susceptible to proteolysis in the stomach and intestine [19]. Although it is possible to prevent degradation through structural modifications [5] or encapsulation in protective excipients [20], its large molecular size and poor water solubility limit its diffusion and permeability through the mucus and the intestinal epithelial barrier, resulting in essentially no drug bioavailability [21].

In recent years, mesoporous silica nanoparticles (MSN) have been used as carriers for peptide and protein drugs, thereby opening new opportunities for oral delivery [22, 23]. Owing to their nanometric size and high biocompatibility, MSN can reach the intestinal compartment while carrying protected cargo [22]. The technological versatility of MSN enables diversification of nanoparticle morphology, particle size, porosity, charge, and surface chemistry, for tailored interactions with intestinal cells [24, 25, 26]. Specifically, dendritic MSN offer tunable pores in the range of 6–20 nm, large enough to host high molecular weight drugs (0.5‒150 kDa), which have consolidated MSN‐based formulations as one of the most promising alternatives for the oral delivery of peptides and biologics [16, 22, 23]. Particularly for insulin delivery, negatively charged MSN with particle sizes below 200 nm can induce the redistribution and relaxation of tight junctions (TJs) by interacting with cell surface integrins, thereby enhancing intestinal permeability of insulin [24]. This permeation‐enhancing effect has also been observed with anionic, virus‐shaped MSN, which efficiently penetrate the mucus layer and traverse the intestinal epithelium to transport insulin [27]. To overcome mucus trapping, MSN have been functionalized with hydrophilic zwitterionic coatings, block copolymers, and cell‐penetrating peptides, facilitating cellular uptake and improving the pharmacological availability of peptide drugs [28, 29]. However, in these formulations, insulin has only been physically combined with the MSN carriers, and the potential of encapsulating the peptide within the silica mesopores has not yet been fully exploited to improve its bioactivity and overall bioavailability upon delivery [28].

Although considerable progress has been made in delivering insulin using MSN‐based formulations through paracellular permeation across the intestinal epithelium [19, 30], little is known about how MSN‐mediated insulin transport affects the biological state and metabolic activity of insulin upon contact with intestinal epithelial cells, or how MSN biodistribution in intestinal tissues contributes to this process. In this context, confinement of insulin within MSN mesopores represents a promising strategy to enhance drug stability in the harsh gastrointestinal environment while enabling intestinal permeation through carrier‐mediated modulation of barrier function [26, 31]. In this regard, insulin‐loaded MSN have been co‐formulated with protein excipients such as succinylated β‐lactoglobulin to obtain pH‐responsive tablet formulations that prevent premature gastric release and enzymatic degradation while promoting insulin internalization in intestinal epithelial cells [32]. However, key aspects, such as the biological activity of the delivered insulin and its paracellular permeability across the intestinal barrier, remain unexplored.

In this work, we developed novel multifunctional MSN‐based formulations for oral peptide delivery, using insulin as a model drug. Insulin was encapsulated into differently functionalized MSN, which were combined with succinylated β‐lactoglobulin (sBL) to obtain tablets that support oral administration. This strategy improved the colloidal stability of the MSN carriers and the insulin‐loading efficiency, ensuring control of the release rate and protection in intestinal conditions, as well as subsequent bioactivity upon interaction with intestinal cells, cellular uptake, and paracellular permeation. The particles were thoroughly characterized by transmission electron microscopy (TEM), dynamic light scattering (DLS), N_2_ physisorption, solid‐state cross‐polarization magic angle spinning nuclear magnetic resonance spectroscopy (CP/MAS NMR), attenuated total reflectance Fourier‐transformed infrared spectroscopy (ATR‐FTIR), and thermogravimetric analysis (TGA). The stability and controlled release of the insulin‐loaded formulations were assessed in gastrointestinal fluids. The ability of insulin‐loaded MSN to be internalized by intestinal cells, as well as the redistribution of TJ proteins to facilitate paracellular permeability, and intracellular distribution of MSN, were evaluated through live cell imaging and microscopy‐based techniques. Furthermore, the bioactivity of the delivered insulin was verified by its ability to support insulin‐responsive signaling pathways, sustaining cell viability and proliferation. Finally, oral delivery and insulin tolerance were assessed in diabetic mice with the developed tablet formulations. Taken together, this study provides new insights into the design and application of functional MSN‐based nanocarriers, offering a translational route for peptide/protein delivery by simultaneously ensuring drug stability, intestinal permeation, and retention of drug bioactivity. To our knowledge, this is the first study to systematically investigate the interplay between insulin release and stability in the gastrointestinal environment and MSN‐mediated insulin transport, integrating transcellular uptake and cellular availability, with insulin permeability via TJ modulation, while correlating these mechanisms with insulin metabolic activity in vitro and validating intestinal tolerability in vivo. Insulin was employed here as a representative model peptide, and the experimental framework and design principles established in this work are expected to be broadly applicable to other peptide drugs and biologics that require protection, transport, and controlled release during oral transit through the gastrointestinal tract.

## 2 Results and Discussion

### 2.1 MSN Functionalization Improves Colloidal Stability and Tailors Particle Surface Chemistry for Oral Insulin Delivery

The MSN were synthesized according to established protocols, yielding high‐quality materials with fine control of particle size, morphology, and porosity [32]. The calcined particles were functionalized with an ethoxy silane‐functionalized polyethylene glycol (PEG‐silane, 2 kDa) and a phosphonated silane, 3‐(trihydroxysilyl)propyl methylphosphonate (THMP), by implementing post‐grafting strategies [33, 34], which generated **PEG‐MSN** and **PO_3_‐MSN**, respectively. The introduction of such hydrophilic functions into the silica structure was expected to enhance the colloidal stability of the MSN carriers, thereby supporting insulin transport across the gastrointestinal tract. PEG has been used to enhance particle diffusion through the mucus layer [31, 35], improving the oral delivery of loaded therapeutics [36]. On the other hand, the increased negative charge of the THMP‐functionalized MSN supports enhanced cell uptake [33].

**FIGURE 1:**
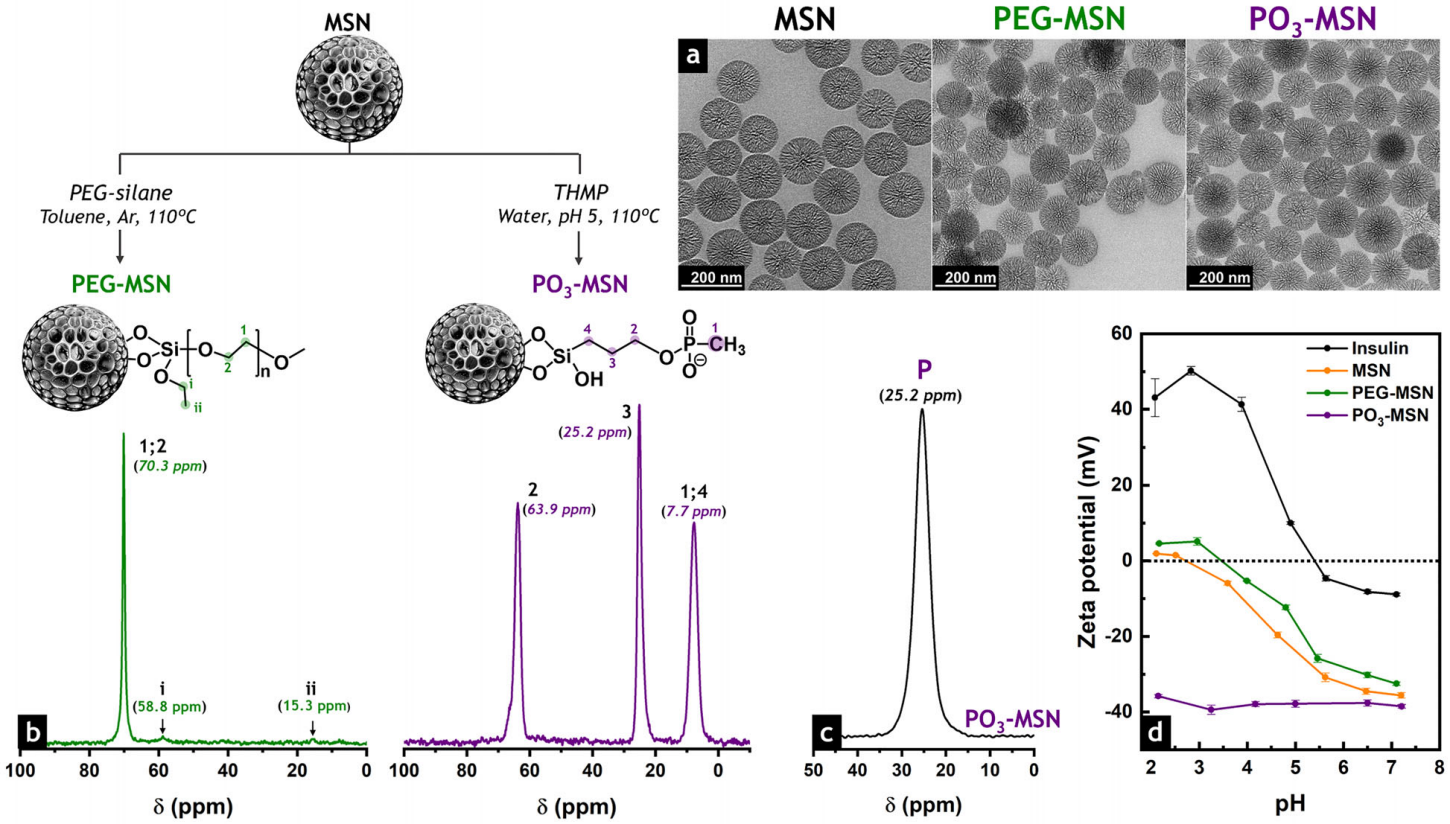
Functionalization of mesoporous silica nanoparticles (MSN) as insulin carriers. Calcined **MSN** were functionalized with polyethylene glycol and phosphonate moieties through post‐grafting strategies to introduce hydrophilic functions with neutral (**PEG‐MSN**) and negative (**PO_3_‐MSN**) charges, respectively. (a) Representative TEM images of **MSN**, **PEG‐MSN**, and **PO_3_‐MSN** exhibiting spherical morphology with dendritic‐like structures. Scale bars represent 200 nm. (b) Solid‐state ^13^C CP/MAS NMR spectra of **PEG‐MSN** (green) and **PO_3_‐MSN** (purple), as well as (c) solid‐state ^31^P CP/MAS NMR spectrum of **PO_3_‐MSN**, correlating the structure of the organosilanes tethered on the silica surface. (d) Zeta potential measurements from pH 2 to 7.4 indicate the range where insulin and the calcined and functionalized MSN exhibit opposite charges, which is essential for their electrostatic interactions during physical adsorption experiments.

TEM images of the calcined and functionalized particles revealed well‐organized, homogeneous dendrimer‐like structures with spherical morphology and particle size of 130 (± 10) nm, which remained unchanged after functionalization (Figure 1a). The hydrodynamic diameters of the modified particles (Figure S1a, Table S1) were measured via DLS, revealing a slight increase relative to the precursor **MSN** (160 ± 1 nm) and consistent with the inclusion of organic moieties on the surface of the **PEG‐MSN** and **PO_3_‐MSN** upon functionalization.

The N_2_ physisorption isotherms of the MSN were measured to characterize their surface area and pore structure, which are essential to ensure subsequent insulin adsorption. Both calcined and functionalized materials displayed isotherms with H3 hysteresis loops due to capillary condensation in mesoporous channels (Figure S2a), typical of center‐radial hierarchical pore systems with a narrow pore size distribution (Figure S2b) [37, 38]. The specific surface area decreased from 828 m^2^·g^−1^ (**MSN**) to 560 m^2^·g^−1^ (**PEG‐MSN**) and 666 m^2^·g^−1^ (**PO_3_‐MSN**) upon functionalization (Table S1). Likewise, the pore size of the calcined **MSN** was reduced from 7.3 to 6.6 nm and 6.1 nm in **PEG‐MSN** and **PO_3_‐MSN**, respectively. The same trend was observed for the pore volume of the grafted materials, which decreased by 26% (**PEG‐MSN**) and 14% (**PO_3_‐MSN**) compared to **MSN** (2 cm^3^·g^−1^). The modification of the porosity of the functionalized nanoparticles correlated with the introduction of organosilanes onto the mesoporous structure, without major pore blocking, still providing sufficient space for later insulin loading [39].

Successful MSN functionalization was confirmed by TGA, which showed the thermal decomposition of the organic moieties incorporated after grafting, measured between 150°C and 700°C (Figure S3a) and corresponded to weight losses of 11 wt% (**PEG‐MSN**) and 10 wt% (**PO_3_‐MSN**) (Table S1). The solid‐state ^29^Si CP/MAS NMR spectra of the functionalized materials exhibited *T* ^2^ and *T* ^3^ signals, confirming the chemical modification of the calcined **MSN** (Figure S4). No *T* ^0^ signals were observed, confirming the absence of non‐covalently attached silanes (Table S2). The resonances in the solid‐state ^13^C CP/MAS NMR spectra of **PEG‐MSN** and **PO_3_‐MSN** aligned with the structure of the molecules tethered onto the silica surface (Figure 1b). The intense peak at 70 ppm in the **PEG‐MSN** spectrum was attributed to the repeating unit of the PEG polymer [34, 40]. The three signals observed in the **PO_3_‐MSN** spectrum corresponded to the carbons of the propyl chain of THMP, together with the carbon of the methyl group of the phosphonate function [33]. The ^31^P CP/MAS NMR spectrum of **PO_3_‐MSN** (Figure 1c) exhibited a single peak at 25.2 ppm, confirming the presence of phosphonate species ROP(O)CH_3_O^–^. C─H stretching bands from aliphatic alkyl chains were observed in the ATR‐FTIR spectra of the functionalized particles in the 2850–2960 cm^−1^ range (Figure S5a), together with the C─H bending of the PEG‐silane at 1456 and 1350 cm^−1^ (**PEG‐MSN**), as well as the P═O stretching band of THMP at 1444 cm^−1^ (**PO_3_‐MSN**). An intense peak corresponding to the asymmetric Si─O─Si stretching of silica was visible around 815 cm^−1^ in the spectra of all the materials obtained. The assignment of the signals observed in the ATR‐FTIR spectra of calcined and functionalized MSN can be found in Table S3.

Chemical modification of MSN directly affects their size distribution and colloidal stability, which are fundamental to ensuring particle transit through the gut and to improving interaction with intestinal cells, uptake, and insulin transport [22]. Therefore, the hydrodynamic diameters of the calcined and functionalized particles were examined in water as a function of pH (Figure S1d). Additionally, phosphate‐buffered saline (PBS, pH 7.4; Figure S1e) was used to disperse the samples and to perform the DLS measurements, simulating human body fluid for biological applications (Figure S1e) [41, 42]. All colloidal dispersions exhibited narrow particle size distributions (PSD) in water and remained stable over a wide pH range (3–7), in correlation with polydispersity indexes (PDI) below 0.3 (Figure S1d), as values above this threshold generally correspond to highly polydisperse samples [43]. In PBS, substantially higher PDIs were observed for **PEG‐MSN**, attributed to particle aggregation (Figure S1e). PDIs above 0.3 were also observed for the calcined **MSN** dispersed in PBS (Figure S1e), albeit with a moderate increase in the hydrodynamic diameter compared to the respective dispersion in water (Figure S1d). The increased ionic strength of PBS reduced the colloidal stability of the **PEG‐MSN**, where the interaction of electrolytes with the neutral polymeric functions and silica surface did not prevent particle aggregation [41]. In turn, **PO_3_‐MSN** exhibited enhanced colloidal stability in both media, with particle size remaining stable over the entire pH range tested. This correlated with the higher negative surface charge of the phosphonated particles compared to calcined **MSN** and **PEG‐MSN**, as confirmed by zeta potential measurements in water (Figure 1d) and PBS (Figure S1f). The differences observed in the zeta potential of the MSN enabled the evaluation of the effect of surface charge on insulin loading, the release of confined insulin, and the interaction of the particles with intestinal cells.

### 2.2 Physical Adsorption of Insulin Ensures Confinement Within Silica Mesopores

**FIGURE 2:**
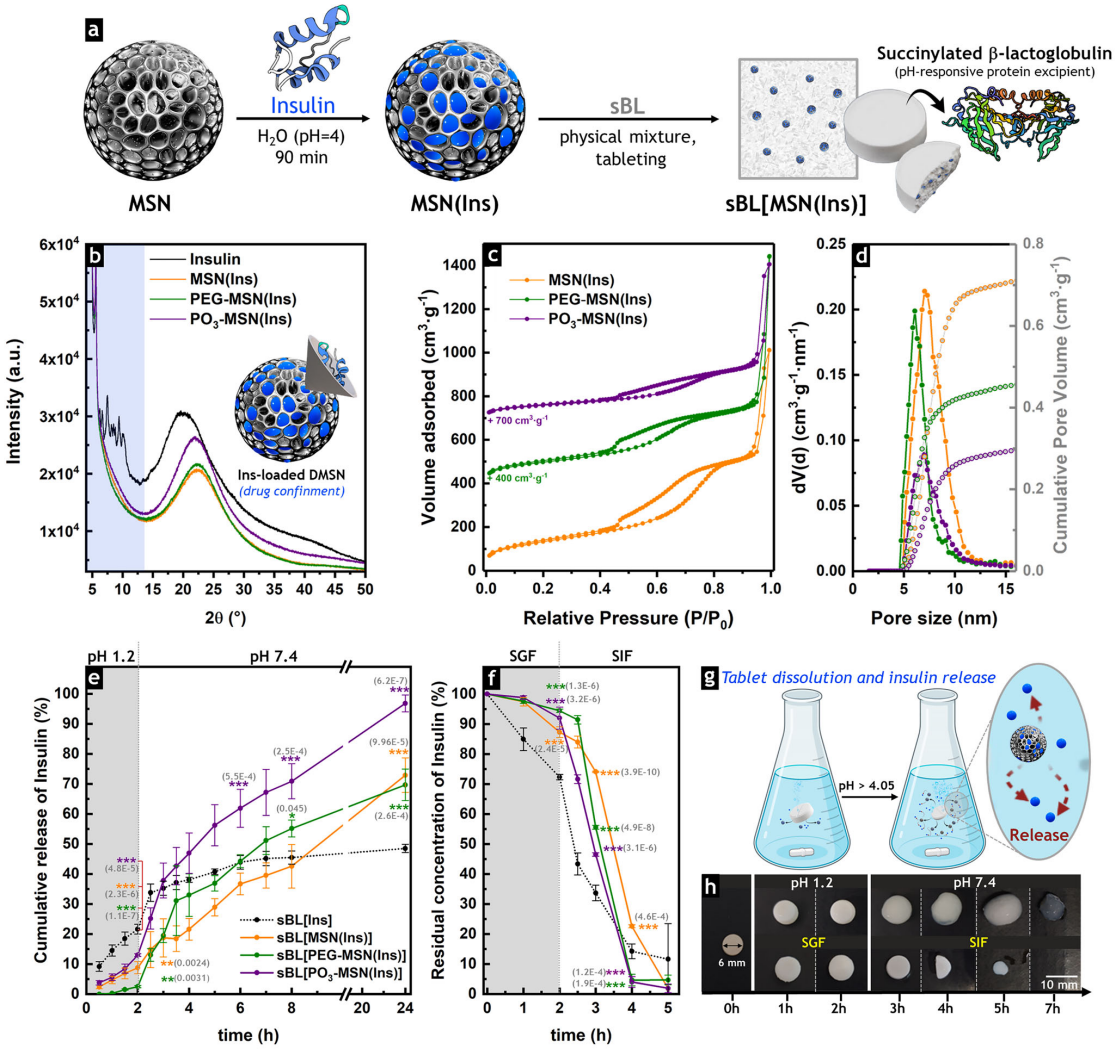
Insulin confinement within mesoporous silica and controlled release from tablet formulations. (a) Insulin was loaded into the mesopores of calcined and functionalized MSN and subsequently tableted with succinylated β‐lactoglobulin (sBL) to obtain pH‐responsive formulations. (b) Wide‐angle XRD patterns of insulin‐loaded MSN (MSN(Ins)) and non‐confined insulin (black) confirmed that insulin lost its semi‐crystalline structure after physical adsorption, supporting its encapsulation within the mesoporous silica network. (c) N_2_ physisorption isotherms (77 K) of MSN(Ins) revealed retention of the mesoporous structure after insulin loading, albeit with a reduction in surface area. (d) Respective pore size distributions (PSD) and cumulative pore volume plots confirmed the reduction of pore size and pore volume after insulin loading. (e) Cumulative insulin release from tablets immersed for 2 h at pH 1.2, followed by immersion at pH 7.4 (control conditions), exhibited enhanced solubility and controlled release when insulin was loaded into the MSN. (f) Residual concentration of insulin (%) after release in simulated gastric fluid (SGF) and simulated intestinal fluid (SIF) confirmed the protective effect of MSN against insulin degradation by digestive enzymes. Data are presented as the mean ± standard deviation (*N* = 3). One‐way ANOVA and Fisher Test were used to highlight significant differences with respect to the control tablets (**sBL[Ins]** without MSN), which are represented by * (*p* < 0.05), ** (*p* < 0.01), and *** (*p* < 0.001). (g) Schematic illustration of the pH‐dependent release of insulin from the tablets (created with BioRender.com). (h) Representative pictures of tablet dissolution over time during release tests in control conditions and in the presence of digestive enzymes (SGF and SIF).

Insulin was loaded into the silica pores through physical adsorption [44]. Zeta potential measurements of insulin in aqueous solutions covering a pH range of 2–7.4 (Figure 1d) indicated that pH 4 was optimal for loading, as insulin and MSN had opposite charges, favoring electrostatic interactions between the positively charged insulin (+41 mV, pH 4) and the negatively charged particles (Table S1) [32]. The loaded particles (**MSN(Ins)**, **PEG‐MSN(Ins)**, and **PO_3_‐MSN(Ins)**) were then combined with sBL and pressed to obtain the pH‐responsive tablet formulations (**sBL[MSN(Ins)]**, Figure 2a).

The incorporation of insulin into MSN was evaluated by TGA (Figure S3d), with loaded amounts (wt%) of 25%, 22%, and 23% in **MSN(Ins)**, **PEG‐MSN(Ins)**, and **PO_3_‐MSN(Ins)**, respectively (Table S1). The slight decrease in the adsorption capacity of the functionalized materials in comparison to **MSN** was attributed to their lower pore size and overall lower porosity (Figure S2b, Table S1).

To verify the physical state and location of loaded insulin within the MSN, TGA was performed in combination with differential scanning calorimetry (DSC). Insulin was physically mixed with calcined or functionalized particles, and the DSC profiles of these samples (Figure S3e) were compared to those of insulin‐loaded MSN (**MSN(Ins)**, **PEG‐MSN(Ins)**, or **PO_3_‐MSN(Ins)**), which contained equivalent amounts of insulin adsorbed into the silica mesopores (Figure S3d). The exothermic peak attributed to insulin decomposition was detected at lower temperatures in the physical mixtures (318°C–360°C) than in the loaded samples (330°C–368°C), indicating insulin confinement within the silica particles upon physical adsorption (Figure S3d). The structure of encapsulated insulin was additionally evaluated by wide‐angle X‐ray diffraction (WA‐XRD). The powder diffraction peaks from the semi‐crystalline structure of non‐confined insulin were not visible in the diffractograms of the insulin‐loaded materials (Figure 2b), supporting that insulin was entrapped inside the mesopores in an amorphous state [32, 45]. N_2_‐physisorption analysis performed on **MSN(Ins)**, **PEG‐MSN(Ins)**, and **PO_3_‐MSN(Ins)** (Figure 2c) revealed a substantial decrease in surface area and pore volume (Figure 2d, Table S1), supporting that most of the insulin was contained within the mesopores.

### 2.3 pH‐Responsive Formulations Protect Insulin Against Proteolysis and Control Drug Release Under Intestinal Conditions

The insulin release from the MSN was assessed according to an established protocol [32] using tablets of sBL combined with insulin (**sBL[Ins]**, control) or with insulin‐loaded particles (**sBL[MSN(Ins)]**, **sBL[PEG‐MSN(Ins)]**, and **sBL[PO_3_‐MSN(Ins)]**). At neutral pH, sBL exists as a non‐covalently linked dimer, exhibiting a negative charge due to the succinyl functions. This increases sBL's solubility and prevents aggregation. However, under acidic conditions, sBL forms larger oligomers that self‐associate into gels due to hydrogen bonds, as well as van der Waals, hydrophobic, and electrostatic interactions [46, 47]. Consistent with this pH‐dependent solubility, the characterization of sBL (200 µg·mL^−1^) by circular dichroism (CD) confirmed distinct structural rearrangements at different pH values (Figure S6a). At pH 1.2, the CD spectrum of sBL showed a pronounced loss of α‐helical and β‐strand signals, along with the appearance of a strong minimum below 200 nm, characteristic of random coil conformations [48, 49] and in accordance with the reduction of protein charge density, aggregation, and gel formation [50]. In contrast, at pH 7.4, the CD spectrum of sBL displayed well‐defined minima at 208 and 222 nm, typical of α‐helices, as well as a broad minimum near 218 nm, indicative of β‐strands, which suggested a more ordered and stable conformation [51]. Compared to native non‐succinylated BL, which retained α‐helical and β‐strand features at both pH values (Figure S6a), sBL (50% succinylation) exhibited enhanced ellipticity at 208 nm under neutral conditions, contrasting with its structural disruption at acidic pH. This correlated with the higher solubility at pH 7.4 and improved pH‐dependent conformational response of sBL relative to non‐succinylated BL [50]. Such structural stability and increased solubility at neutral pH, combined with the observed aggregation in acidic media, underpinned the choice of sBL as a pH‐responsive excipient in the preparation of tablet formulations containing insulin‐loaded MSN (**sBL[MSN(Ins)]**), minimizing premature drug release in the stomach while enabling controlled release in the intestine [47].

Thus, after 2 h in an acidic buffer (pH 1.2), only 3%, 9%, and 13% of insulin were released from **sBL[PEG‐MSN(Ins)]**, **sBL[MSN(Ins)]**, and **sBL[PO_3_‐MSN(Ins)]**, respectively. In contrast, when powders without sBL were tested, almost all the insulin was released within 2 h at pH 1.2 (Figure S7a), with 79%, 72%, and 63% detected in **PEG‐MSN(Ins)**, **MSN(Ins)**, and **PO_3_‐MSN(Ins)** suspensions, respectively. For comparison, 81% of the total concentration of non‐confined insulin (control) was detected in suspension after 2 h, corresponding to 16 µg·mL^−1^ (2.8 µM). Among the tested samples, **PO_3_‐MSN(Ins)** exhibited the lowest release in the absence of sBL (63% insulin release after 2 h at pH 1.2), which was still substantially higher than that observed for the tablet‐based formulation (13% insulin release; **sBL[PO_3_‐MSN(Ins)]**). Overall, these results confirmed the protective effect of sBL in preventing premature insulin release under acidic conditions. On the other hand, in the control tablets (**sBL[Ins]**), 22% of insulin was detected in suspension, which was higher than in all MSN‐based tablet formulations (Figure 2e), highlighting the additional protective effects of the MSN carriers.

After 2 h of incubation under acidic conditions, the tablets were transferred to a buffer at pH 7.4, which is above the isoelectric point of sBL (pH 4.05) [47, 52], allowing for the gradual dissolution of sBL, dispersion of particles, and subsequent drug release (Figure 2g). Sustained insulin release from the MSN‐based formulations was observed over 24 h (Figure 2e), in contrast to **sBL[Ins]**, which dissolved more rapidly and released only 40% of the drug, likely due to the limited solubility of non‐confined insulin [45]. Comparable results were obtained in the control experiment without sBL at pH 7.4, where a maximum concentration of 8.4 µg·mL^−1^ (1.45 µM, equivalent to 42% of the initial insulin) was detected for the non‐confined drug in suspension (Figure S7a). In contrast, maximum release values of 88%, 94%, and 98% were reached for **MSN(Ins)**, **PEG‐MSN(Ins)**, and **PO_3_‐MSN(Ins)**, respectively, after 8 h at pH 7.4 (Figure S7a), which confirmed the enhanced solubility of insulin confined within MSN, and supported near‐complete release profiles under intestinal conditions.

Kinetic analysis of the intestinal‐phase release profiles (Figure S8) further supported a diffusion‐ and hydration‐governed release mechanism from sBL‐based tablet formulations, rather than an erosion‐controlled mechanism. Fitting to zero‐order (Figure S8a), first‐order (Figure S8c), Higuchi (Figure S8e), and Korsmeyer–Peppas (Figure S8g) models was performed using pH 7.4 release data only, with time corrected to reflect the early intestinal phase (0.5–6 h after transfer from pH 1.2 to pH 7.4). This early window was selected to minimize the influence of late‐stage depletion and plateau effects observed at longer times. Insulin release values were normalized to the amount of insulin remaining after the acidic stage (M_r_). Zero‐ and first‐order models (Figure S8a–d) yielded moderate‐to‐high correlations (coefficient of determination *R* ^2^ ≥ 0.90, Table S4); however, residual analysis revealed systematic deviations for all tablet formulations, including the control **sBL[Ins]** and MSN‐based systems (Figure S8b,d). This indicates that insulin release is not governed by a constant‐rate process (zero‐order) nor by purely concentration‐dependent kinetics (first‐order) [53]. The absence of sustained zero‐order behavior ruled out dominant surface erosion of the sBL matrix within the early intestinal window, and instead supported a mechanism driven by progressive hydration, swelling, and dissolution of sBL coupled to insulin‐loaded MSN dispersion [54, 55]. In this context, diffusion‐based and semi‐empirical models further provided mechanistic insight into insulin release under intestinal conditions [53]. In particular, **sBL[PEG‐MSN(Ins)]** and **sBL[PO_3_‐MSN(Ins)]** showed the strongest agreement with diffusion/relaxation‐controlled transport, as evidenced by higher Higuchi constants (*K*_H_ = 21.6 and 27.6%·h^−0.5^, respectively) [56, 57] and anomalous (non‐Fickian) Korsmeyer–Peppas exponents (*n*_KP_ = 0.63 and 0.64, respectively) [55, 58], along with randomly distributed residuals across the fitted range (Figure S8f,h). In contrast, control **sBL[Ins]** tablets exhibited depletion‐dominated release (*n*_KP_ = 0.30, Table S4) [59, 60], consistent with limited insulin solubility and rapid exhaustion of the readily releasable fraction upon matrix hydration [61]. Although Higuchi fits yielded an acceptable correlation for **sBL[Ins]** (*R* ^2^ = 0.98, Table S4), the presence of structured residuals (Figure S8f) indicated that the apparent linearity reflects a restricted fitting window rather than true diffusion control. On the other hand, **sBL[MSN(Ins)]** displayed a mixed transport regime (*K*_H_ = 13.6%·h^−0.5^, *n*_KP_ = 0.73), characterized by structured residuals (Figure S8f,h), indicative of heterogeneous diffusion pathways [55, 58], likely arising from less efficient insulin–silica interactions and non‐homogeneous hydration around the particles compared to the functionalized MSN‐based formulations (**sBL[PEG‐MSN(Ins)]** and **sBL[PO_3_‐MSN(Ins)]**). Overall, these results confirmed the pivotal role of sBL as a time‐dependent, hydration‐responsive excipient that governs tablet dissolution and particle dispersion, thereby enabling diffusion‐controlled release [54], while insulin confinement within MSN mesopores and particle surface chemistry modulate insulin desorption and dispersion under intestinal conditions.

Among the systems studied, **sBL[PO_3_‐MSN(Ins)]** provided the most robust and predictable control over intestinal‐phase insulin release, yielding the highest levels of released insulin (Figure 2e), likely due to the increased negative charge distribution of **PO_3_‐MSN**, which exhibited a zeta potential of −24.3 mV, compared to −19.5 mV for **MSN** and −9.5 mV for **PEG‐MSN** (PBS, pH 7.4, Figure S1f). This favored the dispersion of **PO_3_‐MSN** and enhanced drug diffusion from the silica mesopores into the buffer solution. Thus, after 8 h in pH 7.4 buffer, 71% of insulin was released from **sBL[PO_3_‐MSN(Ins)]**, while only 55% and 43% insulin were released from **sBL[PEG‐MSN(Ins)]** and **sBL[MSN(Ins)]**, respectively. Similar performance was observed after 24 h, where most of the insulin was released from **sBL[PO_3_‐MSN(Ins)]** (97%), compared to 70% and 73% from **sBL[PEG‐MSN(Ins)]** and **sBL[MSN(Ins)]**, respectively, additionally confirming the controlled delayed release enabled by time‐dependent dissolution of the sBL excipient.

Maintaining insulin's native 3D conformation is essential for receptor binding and, consequently, for biological activity [62]. In particular, the α‐helical region of the A‐chain is crucial for insulin interaction with the corresponding receptor; therefore, disruption of this structure leads to loss of activity. Moreover, alterations in secondary structure, such as an increase in β‐sheet content, are indicative of aggregation or denaturation, which ultimately reduces insulin potency [62, 63]. To verify that insulin confinement within MSN and its subsequent release did not compromise its secondary structure, CD spectra of insulin released from sBL‐free formulations (**MSN(Ins)**, **PEG‐MSN(Ins)**, or **PO_3_‐MSN(Ins)**) were collected after 2 h at pH 7.4 and 37°C and compared with those of non‐confined insulin (control) at an equivalent protein concentration (200 µg·mL^−1^, 34.4 µM). The released insulin exhibited characteristic minima at 208 and 222 nm, consistent with an α‐helical secondary structure [51, 64] and closely matched the CD spectrum of non‐confined insulin (Figure S6b). Quantitative analysis of the α‐helical content, estimated from the mean residue ellipticity (MRE, [θ]) at 208 nm [65], yielded values of 51%, 55%, and 52% for insulin released from **MSN(Ins)**, **PEG‐MSN(Ins)**, and **PO_3_‐MSN(Ins)**, respectively, comparable to 55% for non‐confined insulin. These results confirmed the conformational integrity of insulin following confinement and release from MSN carriers and indicate the absence of fibrillation or aggregation [64, 65]. CD characterization of insulin released from **MSN(Ins)**, **PEG‐MSN(Ins)**, or **PO_3_‐MSN(Ins)** complemented the high‐performance liquid chromatography–mass spectrometry (HPLC–MS) analyses used for insulin quantification in the release studies (Figure 2e and Figure S7a), which further confirmed the chemical identity of the released insulin relative to the non‐confined insulin control. Together, these results demonstrate that the confinement and release processes preserved both the native fold and molecular integrity of insulin, which are essential for its biological function.

The control‐release experiment performed in the absence of sBL, using **MSN(Ins)**, **PEG‐MSN(Ins)**, and **PO_3_‐MSN(Ins)**, also supported evaluation of MSN integrity in buffers at pH 1.2 and 7.4. Thus, the particle suspensions obtained after 24 h of incubation at 37°C in both media were characterized by TEM (Figure S7b), which confirmed the long‐term stability of the particle carriers under simulated gastrointestinal conditions, as no major changes in morphology or size were observed for any of the materials tested, including calcined **MSN**, **PEG‐MSN**, and **PO_3_‐MSN**.

To further assess the mechanical stability of the MSN carriers upon tablet compression, insulin loaded into non‐functionalized MSN (**MSN(Ins)**) were subjected to single‐punch compression under the same conditions used for tablet preparation (10 MPa, 2 min), but in the absence of sBL. N_2_ physisorption analysis of the compressed **MSN(Ins)** (Figure S2c–d) revealed a pronounced reduction in surface area (77% decrease) and pore volume (81% decrease) relative to the insulin‐loaded sample prior to compression with sBL. On the other hand, mesopore size remained unchanged (6.8 nm, Table S1), which suggested that mechanical compression did not induce pore collapse or framework damage, but rather resulted in partial pore‐mouth shielding, increased interparticle contacts, and local densification of the silica network. The preservation of pore size additionally indicated the retention of insulin confined within the silica network after compression. Moreover, in the tablet formulations containing sBL (**sBL[MSN(Ins)]**, **sBL[PEG‐MSN(Ins)]**, and **sBL[PO_3_‐MSN(Ins)]**), insulin‐loaded MSN were dispersed within the protein excipient matrix, accounting for only 5 wt.% of the tablet composition. Therefore, sBL is expected to substantially attenuate the mechanical stress experienced by the MSN carriers during tableting.

The stability of the insulin in the tablet formulations was additionally tested in simulated gastric fluid (SGF) and simulated intestinal fluid (SIF) containing relevant digestive enzymes (Figure 2f). The gastric environment was mimicked by incorporating acid‐activated porcine pepsin into a pH 1.2 buffer (SGF). Additionally, intestinal conditions were simulated by adding the commercially available pancreatin, a crude porcine mixture of peptidases, amylases, and lipases, to a salt‐containing buffer at pH 6.8 (SIF) [8, 66]. In both SGF and SIF, digestive enzymes rapidly degraded 90% of insulin within 5 min (Figure S9a). However, for the sBL tablets, most of the insulin remained in the MSN‐based formulations in acidic conditions (Figure 2f). Accordingly, after 2 h in SGF, only 13%, 6%, and 8% of the loaded insulin were released from **sBL[MSN(Ins)]**, **sBL[PEG‐MSN(Ins)]**, and **sBL[PO_3_‐MSN(Ins)]**, respectively, compared to 28% released from **sBL[Ins]**. While the sBL tablet formulations ensured the preservation of insulin in acid conditions (buffer at pH 1.2 and SGF, Figure 2g), the tablets dissolved faster in SIF than in water (Figure 2h) due to the additional enzymatic degradation of sBL (Figure S9b) [67], releasing insulin faster. Within this context, the insulin‐loaded MSN formulations retained insulin significantly longer (*p* < 0.001) than control **sBL[Ins]** (Figure 2f), confirming additional protection by the nanoparticle carriers.

### 2.4 Insulin Delivered by MSN Supports the Viability of Intestinal Cells

The biological activity of the insulin‐loaded MSN was initially characterized in human colonic epithelial cells (HCEC‐1CT), a cytogenetically non‐tumorigenic model widely used for studying colon stem cell biology [68]. HCEC‐1CT cells maintain expression of cell‐type‐specific markers and functions under culture conditions without growth limitations [69, 70]. The essential insulin/transferrin/selenium‐G supplement was removed from the cell growth medium to generate starvation conditions and selectively evaluate the contribution of the peptide when delivered with the MSN carriers [32]. Insulin deprivation disrupts key metabolic pathways, including lipogenesis, glycogen synthesis, and glucose metabolism, thereby reducing cellular function and proliferation [71, 72]. Thus, this cell model was used to verify if the insulin delivered by MSN could be functionally used by the intestinal cells to maintain cell viability when no other insulin source is available. Metabolic activity was then evaluated using **MSN(Ins)**, **PEG‐MSN(Ins)**, or **PO_3_‐MSN(Ins)** and compared to non‐confined insulin supplied through the cell culture medium (**Ins^(+)^**, 10 µg·mL^−1^, 1.7 µM).

HCEC‐1CT cell viability was quantified using the WST‐1 cell proliferation reagent (4‐[3‐(4‐iodophenyl)‐2‐(4‐nitrophenyl)‐2H‐5‐tetrazolio]‐1,3‐benzene sulfonate). HCEC‐1CT cells were incubated under insulin‐deprivation (insulin‐free medium, **Ins^(−)^**) or with standard insulin‐containing medium (positive control, **Ins^(+)^**) for 1, 3, or 24 h (Figure S10a), which did not affect cell viability, regardless of incubation time of culture media (**Ins^(−)^** or **Ins^(+)^**). Since a decrease in cell proliferation could not be detected in starvation or standard culture conditions, after such pre‐incubations, cells were treated for 3 h at 37°C with insulin (10 µg mL^−1^, 1.7 µM), provided either by the insulin‐loaded MSN (**MSN(Ins)**, **PEG‐MSN(Ins)**, or **PO_3_‐MSN(Ins)**) or through an insulin‐containing culture medium **Ins^(+)^** (Figure S10b). To isolate any particle‐specific effects, the cells were alternatively incubated with empty MSN (**MSN**, **PEG‐MSN**, or **PO_3_‐MSN**) in the complete culture medium (**Ins^(+)^**, 10 µg·mL^−1^, 1.7 µM) at an equivalent particle concentration as in the insulin‐loaded samples (40 µg·mL^−1^). Overall, the application of both insulin‐loaded MSNs in **Ins^(−)^** and empty MSN in **Ins^(+)^** media triggered an increase in WST‐1 cell metabolism and cell proliferation compared to the controls (non‐treated cells incubated with **Ins^(+)^**), which reflects the stimulation of metabolic activity of the cells in the presence of nanoparticulate materials [32]. This effect, observed after pre‐incubation for 1 and 3 h under starving conditions (**Ins^(−)^**) followed by MSN treatments, was attributed to the calcined or functionalized MSN, regardless of whether insulin was confined into the particles (**MSN(Ins)**, **PEG‐MSN(Ins)**, or **PO_3_‐MSN(Ins)**) or supplemented in the cell culture medium. Instead, longer pre‐incubation of 24 h in control media (**Ins^(−)^** or **Ins^(+)^**) followed by treatments with insulin‐loaded MSN or empty particles rendered cell proliferation similar to the positive control (non‐treated cells incubated with **Ins^(+)^**), probably due to cell adaptation to the cell culture conditions (Figure S10b). These observations correlated with measurements of cell biomass using the Crystal Violet assay (Figure S10c), confirming the absence of cytotoxicity of the calcined and functionalized MSN and suggesting MSN uptake.

**FIGURE 3:**
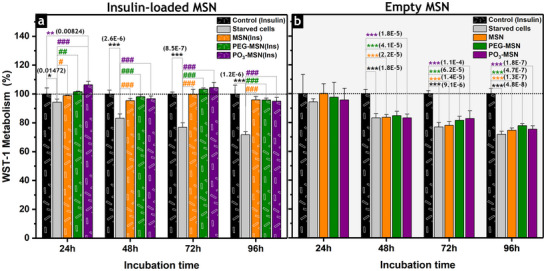
Viability of HCEC‐1CT cells measured by WST‐1 assay upon treatments with insulin‐loaded MSN or empty MSN, either calcined or differently functionalized. The positive control corresponds to HCEC‐1CT cells incubated in a complete medium (**Ins^(+)^**, 10 µg·mL^−1^, 1.7 µM) with no additional treatment (cells under standard culture conditions, black bars). The negative control corresponds to HCEC‐1CT cells incubated in the insulin‐free medium (**Ins^(−)^**) with no additional treatment (cells under starving conditions, gray bars). (a) WST‐1 metabolism/cell viability was measured after cell incubation with insulin (10 µg·mL^−1^, 1.7 µM), provided either through the cell culture medium (control, **Ins^(+)^**) or through the insulin‐loaded MSN (**MSN(Ins)**, **PEG‐MSN(Ins)**, or **PO_3_‐MSN(Ins)**). The negative control (**Ins^(−)^**, gray bars) showed a significant drop in cell viability, while the treatment with insulin‐loaded MSN maintained cell viability, as in the positive control (**Ins^(+)^**, black bars). (b) WST‐1 metabolism upon treatment with empty particles (**MSN**, **PEG‐MSN**, or **PO_3_‐MSN**) in starving conditions. Only the positive control (**Ins^(+)^**, black bars) retained viability over time, ruling out any effects in long‐term cell viability related to empty MSN. Data sets were obtained from three independent cell preparations (biological triplicates; *N* = 3), measured in technical duplicates, and presented as mean ± standard deviation. One‐way ANOVA and Fisher Test, expressing significant differences with respect to the positive control (**Ins^(+)^**, *) or starved cells (**Ins^(−)^**, negative control, #), are indicated with */# (*p* < 0.05), **/## (*p* < 0.01), or ***/### (*p* < 0.001).

To assess long‐term effects of MSN treatments on viability, HCEC‐1CT cells were incubated with insulin‐loaded MSN (**MSN(Ins)**, **PEG‐MSN(Ins)**, or **PO_3_‐MSN(Ins)**) for up to 96 h (Figure 3a) under starving conditions (**Ins^(−)^** medium) and compared to empty **MSN**, **PEG‐MSN**, or **PO_3_‐MSN** in insulin‐free medium (**Ins^(−)^**). This experimental layout enabled the evaluation of potential responses associated with the unloaded MSN (Figure 3b). This experiment also provided information on the long‐term effects of insulin starvation on cell viability, which were not significant after incubation in **Ins^(−)^** up to 24 h (Figure S10a).

Cells treated with insulin‐loaded MSN formulations exhibited WST‐1 metabolism comparable to that of the positive control (non‐treated cells, **Ins^(+)^** media) even after 96 h of incubation, which confirmed that the insulin provided by the particle formulations efficiently supported cell viability. In contrast, the WST‐1 metabolism of the negative control (non‐treated cells, **Ins^(−)^** media) decreased progressively, indicating that cells did not tolerate insulin deprivation for more than 24 h (Figure 3a). Under starvation conditions, incubation with empty **MSN**, **PEG‐MSN**, or **PO_3_‐MSN** did not restore cell viability over 96 h, supporting that the preservation of cell viability can be exclusively attributed to the insulin delivery from the MSN formulations rather than cell proliferation associated with the presence of empty nanoparticles (Figure 3b). Overall, these results demonstrated the retention of insulin's biological activity following nanoparticle‐mediated delivery.

### 2.5 Functionalized MSN Carriers Improve Intestinal Epithelial Particle Uptake and Insulin Availability

Live‐cell imaging experiments were performed to further assess whether the nanoparticles were taken up by cells and whether the insulin cargo could be visualized, supporting intestinal availability and the observed viability of HCEC‐1CT cells (Figure 3). For this purpose, the MSN carriers were labeled with the red fluorophore rhodamine B isothiocyanate (Rhod‐ITC, Scheme S1), and insulin with the green fluorophore fluorescein isothiocyanate (FITC, Scheme S3). The UV–Vis spectra of aqueous dispersions of the rhodamine‐labeled materials (**MSN^Rhod^**, **PEG‐MSN^Rhod^**, and **PO_3_‐MSN^Rhod^**) exhibited the expected absorbance band at 559 nm (Figure S11a), corresponding to grafted rhodamine [73]. The ATR‐FTIR spectra of **MSN^Rhod^**, **PEG‐MSN^Rhod^**, and **PO_3_‐MSN^Rhod^** confirmed the particle labeling (Figure S5b, Table S3), which correlated with the incorporation of 3 wt% of rhodamine determined by TGA (Figure S3b, Table S1). The preservation of the size (Figure S1b) and negative surface charge (Table S1) of the MSN after rhodamine labeling was validated by DLS. Additionally, N_2_ physisorption analysis confirmed that the fluorescent MSN retained their porosity appropriate for insulin loading into the mesoporous structure (Figure S2e,f).

Insulin was labeled with FITC according to a standard protocol (Scheme S3) [32, 74]. Purification and subsequent characterization of the modified insulin (**Ins^Flu^**) by high‐resolution electrospray ionization mass spectrometry (HR‐ESI‐MS) confirmed successful labeling, yielding a distribution of labeled insulin species bearing up to three fluorescein moieties per peptide molecule (Figure S12). **Ins^Flu^** was loaded into the rhodamine‐labeled MSN (**MSN^Rhod^**, **PEG‐ MSN^Rhod^**, or **PO_3_‐MSN^Rhod^**) following the same protocol performed for the non‐fluorescent materials. The intensity of the absorption bands of the UV–Vis spectrum of **Ins^Flu^** was drastically reduced after the interaction of fluorescent insulin with the rhodamine‐labeled particles, which was verified when the supernatants obtained after the isolation of the loaded samples (**MSN^Rhod^(Ins^Flu^)**, **PEG‐MSN^Rhod^(Ins^Flu^)**, **PO_3_‐MSN^Rhod^(Ins^Flu^)**) were analyzed (Figure S11b). This confirmed the incorporation of insulin into the silica particles, in accordance with a mass loss of about 20 wt% detected by TGA, which was attributed to the thermal decomposition of the loaded **Ins^Flu^** (Figure S3f, Table S1). The DSC profiles of the fluorescent insulin‐loaded materials aligned with those obtained for the equivalent non‐fluorescent **MSN(Ins)**, **PEG‐MSN(Ins)**, and **PO_3_‐MSN(Ins)** (Figure S3d), as evidence of insulin confinement within rhodamine‐labeled silica.

**FIGURE 4:**
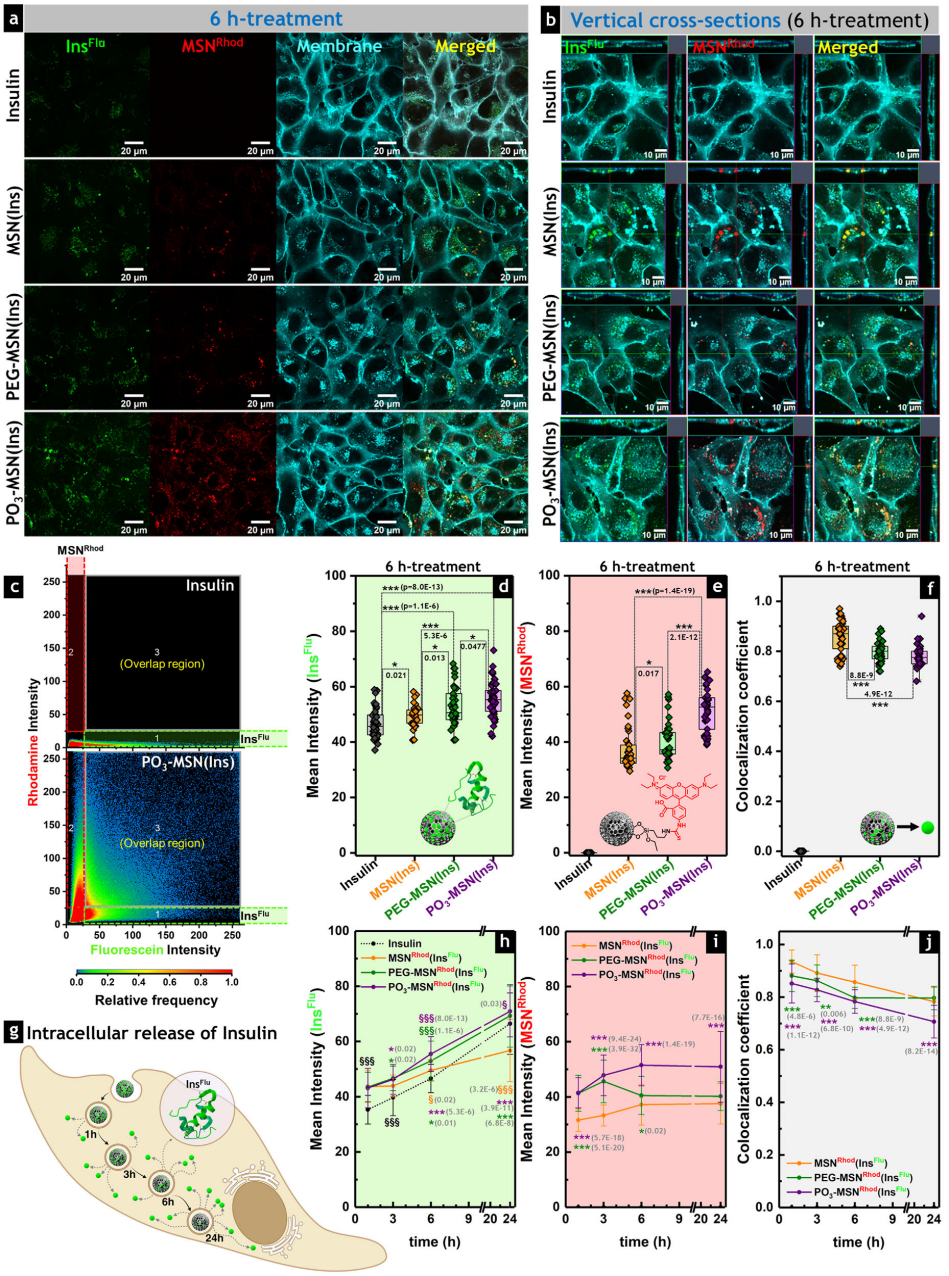
Uptake of insulin‐loaded MSN by intestinal cells and controlled intracellular release of insulin. (a) Representative live cell fluorescence images of HCEC‐1CT cells incubated with insulin‐loaded MSN (MSN^Rhod^(Ins^Flu^)) for 6 h at 37°C. The fluorescence signals from Ins^Flu^ and MSN^Rhod^ were simultaneously detected and represented in green (Ins^Flu^) and red (MSN^Rhod^), respectively. The plasma membrane is represented in cyan (CellMask deep red plasma membrane stain). Scale bars stand for 20 µm. (b) Vertical cross‐sections of 3D cut stacks proved insulin internalization and MSN uptake. Scale bars stand for 10 µm (c) Representative colocalization graphs of cells treated with non‐confined insulin (**Ins^Flu^**, control) and insulin loaded on phosphonated particles (**PO_3_‐MSN^Rhod^(Ins)^Flu^**), showing the signals of fluorescein (**1**: Ins^Flu^), rhodamine (**2**: MSN^Rhod^), and simple spatial overlap of both signals (**3**: Overlap region). Quantification of the fluorescence intensities of (d) Ins^Flu^ and (e) MSN^Rhod^, as well as (f) colocalization coefficient after 6 h‐incubation with MSN^Rhod^(Ins^Flu^), confirmed enhanced intracellular delivery and release of insulin when confined into MSN. Data sets were obtained from three independent cell preparations (biological triplicates; *N* = 3) and quantified from more than 25 regions of interest (*n* ≥ 25). One‐way ANOVA and Fisher Test expressed significant differences by *(*p* < 0.05), **(*p* < 0.01) and ***(*p* < 0.001). (g) Schematic representation of the intracellular release of insulin after the uptake of insulin‐loaded MSN (created with BioRender.com). Evolution of the fluorescence intensities of (h) **Ins^Flu^** and (i) rhodamine‐labeled MSN (**MSN^Rhod^**, **PEG‐MSN^Rhod^**, or **PO_3_‐MSN^Rhod^**), as well as (j) the colocalization coefficient over time, correlating with the intracellular release of insulin. Data is shown as mean ± standard deviation. Statistically significant differences according to One‐way ANOVA and Fisher Test when treatments are compared with respect to the non‐confined insulin (**Ins^Flu^**, §), or insulin‐loaded into calcined particles (**MSN^Rhod^(Ins^Flu^)**, *) are shown with §/* (*p* < 0.05), §§/** (*p* < 0.01), or §§§/*** (*p* < 0.001).

The ability of the **MSN^Rhod^(Ins^Flu^)**, **PEG‐MSN^Rhod^(Ins^Flu^)**, and **PO_3_‐MSN^Rhod^(Ins^Flu^)** to be internalized by intestinal cells was tested by confocal microscopy. Live cell imaging was performed after incubation of the HCEC‐1CT cells for 1 h (Figure S13a), 3 h (Figure S13c), 6 h (Figure 4a), and 24 h (Figure S13e) with the insulin‐loaded particles. For such experiments, both calcined and functionalized rhodamine‐labeled MSN (**MSN^Rhod^**, **PEG‐MSN^Rhod^**, or **PO_3_‐MSN^Rhod^**) were used, containing **Ins^Flu^** loaded into the mesoporous silica. This experiment was specifically designed to simultaneously observe the distribution of insulin (green) and nanoparticles (red). For all treatments, the cell monolayer retained integrity, with no apparent detachment or change in cell morphology.

Vertical cross‐section images acquired at shorter incubations of 1 and 3 h revealed that non‐confined **Ins^Flu^** (control) was scarcely internalized (Figure S13). After incubation for 1 h (Figure S13b) and 3 h (Figure S13d), fluorescence intensities observed for the free **Ins^Flu^** (control) were lower in comparison to particle treatments, and the peptide drug was mainly distributed in the paracellular space and the proximity of the plasma membrane. By contrast, rhodamine‐labeled particles (**MSN^Rhod^**, **PEG‐MSN^Rhod^**, and **PO_3_‐MSN^Rhod^**) seemed to favor **Ins^Flu^** accumulation in discrete areas within the cytoplasm, which allowed stronger detection of the fluorescence signal of the confined **Ins^Flu^** (**MSN^Rhod^(Ins^Flu^)**, **PEG‐MSN^Rhod^(Ins^Flu^)**, and **PO_3_‐MSN^Rhod^(Ins^Flu^)**), supporting the idea that insulin‐cargo availability could be assisted by particle formulation.

After 6 h of incubation (Figure 4b), intestinal cells internalized both the control (non‐confined **Ins^Flu^**) and **Ins^Flu^** loaded into rhodamine‐labeled MSN, which aligned with the cytotoxicity experiments and implied that intestinal cells processed free insulin and insulin delivered by the particles similarly. As this timescale aligns with the maximum transit time through the small intestine [75], 6 h was selected as the main time point for further analysis. In such conditions, the highest fluorescence intensities were observed when insulin was delivered *via* particle formulations (**MSN^Rhod^(Ins^Flu^)**, **PEG‐MSN^Rhod^(Ins^Flu^)**, and **PO_3_‐MSN^Rhod^(Ins^Flu^)**), supporting the interpretation that particles shuttled **Ins^Flu^** transport into intestinal cells—likely through efficient interaction with the cell membrane, not necessarily dependent on insulin receptors, making it a valuable model also for the delivery of other cargo peptides. Additionally, 2.5D views from live‐cell imaging confirmed that fluorescence intensity in both the fluorescein and rhodamine channels increased progressively over time (Figure S14), indicating time‐dependent internalization of insulin‐loaded MSNs.

After 24 h of incubation, the fluorescein signals of the non‐confined **Ins^Flu^** (control) and the insulin‐loaded functionalized MSN (**PEG‐MSN^Rhod^(Ins^Flu^)** and **PO_3_‐MSN^Rhod^(Ins^Flu^)**) were similar, but higher than for the **MSN^Rhod^(Ins^Flu^)** treatment (Figure S13e,f), confirming the impact of the different MSN surface functionalization on insulin transport and intracellular drug delivery. To quantify these observations and provide a more detailed overview of the release of the labeled **Ins^Flu^** from the rhodamine‐labeled carriers (**MSN^Rhod^**, **PEG‐MSN^Rhod^**, and **PO_3_‐MSN^Rhod^**), as well as of the crucial role of particle functionalization, colocalization graphs were analyzed (Figure S15). Mean fluorescence intensities of fluorescein (region 1) and rhodamine (region 2) were independently acquired from such colocalization graphs (Figure 4c). This analysis also provided the colocalization coefficient (region 3), which expresses the degree of linear correlation in pixel‐by‐pixel intensity between the two fluorescence distributions. The lower the value of the colocalization coefficient, the more spatially separated the fluorescent signals were considered to be within the cell. Therefore, this parameter was taken as an indication of intracellular insulin release from the loaded **MSN^Rhod^(Ins^Flu^)**, **PEG‐MSN^Rhod^(Ins^Flu^)**, and **PO_3_‐MSN^Rhod^(Ins^Flu^)** (Figure 4g).

Quantification was performed for all treatments at different incubation times (1, 3, 6, or 24 h) tested (Figure S16, Figure 4d–f). Overall, insulin loaded into functionalized **PEG‐MSN^Rhod^(Ins^Flu^)** and **PO_3_‐MSN^Rhod^(Ins^Flu^**) returned higher fluorescein signal intensities in comparison to both control (**Ins^Flu^**) and **MSN^Rhod^(Ins^Flu^)**, which were correlated with increased particle uptake (quantified from rhodamine fluorescence), considering that the amount of insulin loaded on all materials was equivalent and administered at the same concentration as the non‐confined **Ins^Flu^**. This suggests that surface functionalization not only enhances the colloidal stability of the nanoparticles and controlled insulin release but also affects particle interactions with intestinal cells and insulin transport. For example, after a 6 h incubation, the highest fluorescein intensity was observed when the cells were treated with the phosphonated, functionalized **PO_3_‐MSN^Rhod^(Ins^Flu^**) (Figure 4d). Furthermore, the increased rhodamine intensity of **PO_3_‐MSN^Rhod^** confirmed the most efficient internalization of the phosphonated nanoparticles (Figure 4e), homogeneously distributed in the cytoplasm (Figure 4a). The intracellular distribution of **PO_3_‐MSN^Rhod^** also favored insulin release, consistent with a lower colocalization coefficient of **PO_3_‐MSN^Rhod^(Ins^Flu^**) compared with the other formulations tested (Figure 4f).

Time‐resolved fluorescence intensity profiles of both fluorescein (Figure 4h) and rhodamine (Figure 4i), together with the corresponding colocalization coefficients for each treatment (Figure 4j), were obtained. Although the intensity of **Ins^Flu^** was higher when the cells were treated with functionalized MSN with respect to the control (non‐confined **Ins^Flu^**, Figure 4h), the insulin release from the PEGylated formulation (**PEG‐MSN^Rhod^(Ins^Flu^)**) did not increase further after 6 h‐incubation (Figure 4j). This was attributed to limited internalization of **PEG‐MSN^Rhod^** due to particle aggregation and a lack of colloidal stability, in agreement with the reduction in rhodamine fluorescence intensity of **PEG‐MSN^Rhod^** after 6 h relative to shorter incubation times (Figure 4i). The decrease observed in the colocalization coefficient (Figure 4j) for all particle treatments (**MSN^Rhod^(Ins^Flu^)**, **PEG‐MSN^Rhod^(Ins^Flu^)**, **PO_3_‐MSN^Rhod^(Ins^Flu^)**) correlated with the release profile of the tablet formulations in buffered media (pH 7.4, Figure 2e), indicating reproducible release kinetics in the intestinal cell model.

To demonstrate that the internalization of insulin‐loaded MSN was not probe‐dependent and to rule out potential interferences arising from the labeling procedures, the experiments were repeated using a different combination of grafted fluorophores. Thus, insulin was labeled with rhodamine (**Ins^Rhod^**), as shown in Figure S17f. Furthermore, MSN carriers, both calcined and functionalized, were labeled with fluorescein (**MSN^Flu^**, **PEG‐MSN^Flu^**, **PO_3_‐MSN^Flu^**), as represented in Scheme S2, each containing 3 wt.% of the fluorophore (Figure S3c, Table S1). **Ins^Rhod^** was then loaded into the fluorescein‐labeled MSN, and the cells were incubated with the formulations obtained (**MSN^Flu^(Ins^Rhod^)**, **PEG‐MSN^Flu^(Ins^Rhod^)**, or **PO_3_‐MSN^Flu^(Ins^Rhod^)**). The fluorescein and rhodamine signals were quantified upon treatment for 1, 3, 6, and 24 h (Figure S17a–d), and cell uptake was verified via vertical cross‐section images (Figure S17e). As for **MSN^Rhod^(Ins^Flu^)**, **PEG‐MSN^Rhod^(Ins^Flu^)**, and **PO_3_‐MSN^Rhod^(Ins^Flu^)** treatments, the signal of the **Ins^Rhod^** (Figure S17f) was boosted by the fluorescein‐labeled particles (**MSN^Flu^**, **PEG‐MSN^Flu^**, and **PO_3_‐MSN^Flu^**), rendering higher rhodamine fluorescence intensities when insulin was confined into the functionalized materials (Figure S17g). Furthermore, the change in fluorescein intensity over time correlated well with particle uptake (Figure S17h) and followed the same trend as that observed for the **MSN^Rhod^**‐, **PEG‐MSN^Rhod^‐**, and **PO_3_‐MSN^Rhod^**‐based formulations (Figure 4i). Likewise, the colocalization coefficient obtained from colocalization analysis correlated with insulin release, regardless of the fluorophore combinations used to label the MSN and insulin (Figures 4j and S17i). These results supported the conclusion that the differences between treatments were attributable to the biological performance of the insulin‐loaded MSN rather than to quantification artifacts, bias from the selection of optical fields, or noise from “free” fluorophores not attached to insulin or MSN. Therefore, the reproducibility of the observed responses and the robustness of the quantification method were confirmed.

### 2.6 Insulin‐Loaded MSN Uptake is Mediated by Energy‐Dependent Endocytosis

**FIGURE 5:**
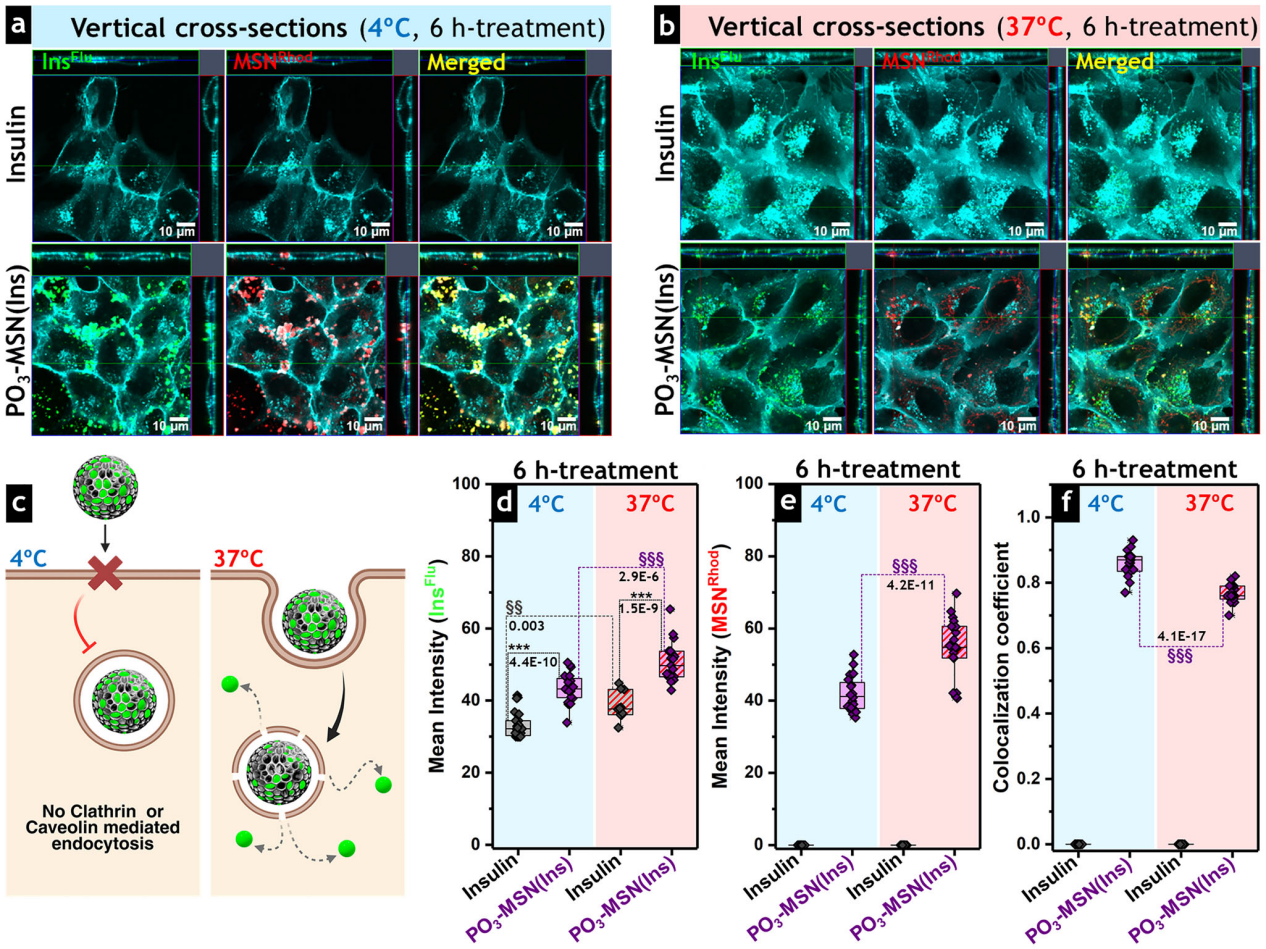
Energy‐dependent endocytosis of insulin‐loaded MSN. Cross‐sections of the 3D cut stacks obtained from Z‐stack imaging after 6 h‐incubation of HCEC‐1CT cells with **Ins^Flu^** (control) and **PO_3_‐MSN^Rhod^(Ins^Flu^)** at (a) 4°C and (b) 37°C. The fluorescence signals corresponding to **Ins^Flu^** and **PO_3_‐MSN^Rhod^** are represented in green and red, respectively, and show limited internalization of both insulin and MSN at 4°C. The plasma membrane is depicted in cyan (CellMask deep red plasma membrane stain). Scale bars stand for 10 µm. (c) Schematic representation of the temperature‐mediated particle uptake and subsequent intracellular release of insulin (created with BioRender.com). Quantification of the fluorescence intensity of (d) **Ins^Flu^**, (e) **PO_3_‐MSN^Rhod^**, as well as (f) the colocalization coefficient of both signals after 6 h‐incubation with **PO_3_‐MSN^Rhod^(Ins^Flu^)** at 4°C or 37°C confirmed enhanced particle uptake and intracellular insulin release when the cells were incubated at 37°C. Data sets were obtained in three independent cell preparations (biological triplicates; *N* = 3) by quantifying more than 20 regions of interest (*n* ≥ 20). The statistically significant differences according to One‐way ANOVA and Fisher Test, when treatments were compared at the same temperature (*) or when the effect of temperature was analyzed (§), are indicated with **/§§ (*p* < 0.01) and ***/§§§ (*p* < 0.001).

To analyze the internalization mechanisms of insulin‐loaded MSN, cells were imaged after 6 h of incubation with **PO_3_‐MSN^Rhod^(Ins^Flu^)** at 37°C and 4°C (Figure S18). Endocytosis, one of the most common cellular uptake mechanisms, is an energy‐dependent process that can be reduced by decreasing the temperature to 4°C [76]. Such dependence on temperature is closely related to alterations in the fluidity of the lipid bilayer and the dynamics of the cytoskeleton [77]. Low temperatures reduce ATP production, limiting the energy required for vesicle formation, motor protein function, and membrane fusion, thereby slowing or inhibiting endocytosis [78]. Thus, at 4°C and 6 h of incubation, **PO_3_**‐**MSN^Rhod^(Ins^Flu^)** was scarcely observed inside the cells but formed aggregates outside the membrane (Figure 5a). Similarly, the fluorescence signal of non‐confined **Ins^Flu^** (positive control) was predominantly detected in the extracellular space but exhibited significantly lower overall fluorescence intensity (Figure 5d, *p* = 4.4 × 10^−10^) than **PO_3_**‐**MSN^Rhod^(Ins^Flu^)**. Furthermore, the fluorescein signal corresponding to confined insulin **Ins^Flu^** in **PO_3_**‐**MSN^Rhod^(Ins^Flu^)** largely overlapped with the rhodamine fluorescence from particle carriers (**PO_3_**‐**MSN^Rhod^**), indicating limited insulin release/availability at low temperatures (Figure 5f). By contrast, 6 h incubation at 37°C resulted in strong fluorescence signals of both fluorescein and rhodamine, homogeneously distributed in the cytoplasmic compartment (Figure 5b).

The change in the intensity and distribution of fluorescence signals with temperature supports the involvement of endocytic pathways in internalizing the non‐confined **Ins^Flu^** and insulin‐loaded **PO_3_**‐**MSN^Rhod^(Ins^Flu^)** via interaction with insulin‐ [79] or clathrin/caveolin‐receptors (Figure 5c) [80], respectively. Colocalization analysis (Figure S18e,f) confirmed that fluorescein intensity from labeled insulin was higher when the cells were incubated with both non‐confined **Ins^Flu^** and **PO_3_**‐**MSN^Rhod^(Ins^Flu^)** at 37°C compared to fluorescein intensity obtained upon cell treatments at 4°C (Figure 5d). Likewise, the uptake of the phosphonated particles was triggered by incubation at physiological temperature, as evidenced by increased rhodamine fluorescence (Figure 5e). In contrast, incubation at 4°C impeded insulin release, thereby limiting its intracellular diffusion. This was associated with a significantly higher colocalization coefficient at 4°C (*p* = 4.1 × 10^−17^) than at 37°C (Figure 5f). Energy‐dependent endocytosis was also confirmed when intestinal cells were incubated for 3 h with **Ins^Flu^** and **PO_3_**‐**MSN^Rhod^(Ins^Flu^)**, showing the same responses, albeit with lower mean fluorescence intensities due to the shorter incubation time (Figure S18g–i).

The presence of insulin in HCEC‐1CT cells following peptide delivery via MSN was further supported by proteomics analysis (Figure S19a). Cells were incubated for 6 h at 37°C with non‐confined insulin (positive control, **Ins^(+)^**) or with insulin‐loaded MSN (**MSNs(Ins)**, **PEG‐MSNs(Ins)**, or **PO_3_‐MSNs(Ins)**). Both the positive control and the particle formulations were dispersed in insulin‐free cell culture medium, ensuring an equivalent insulin concentration (10 µg·mL^−1^, 17 µM). Starved cells incubated in insulin‐free medium were included as a negative control (**Ins^(−)^**). With this untargeted approach, insulin was detected in both the positive control **Ins^(+)^** and cells treated with insulin‐loaded particles, whereas no insulin signal was detected in starved cells (Figure S19b). Together, these results align with the other datasets, supporting intestinal insulin availability upon MSN‐mediated delivery, and are consistent with the preserved cell viability observed under insulin‐supplemented conditions (Figure 3), as well as with peptide detection by fluorescence microscopy (Figures 4 and S13).

Overall, **PO_3_‐MSN(Ins)** exhibited enhanced particle uptake, supporting increased intestinal availability of the delivered insulin, as well as improved colloidal stability and controlled release under gastrointestinal‐mimicking conditions. These findings highlight the technological potential of **PO_3_‐MSN(Ins)**‐based formulations; however, key aspects of insulin bioactivity following cellular uptake and its capacity to traverse the intestinal barrier remain to be evaluated. These questions prompted further investigation of paracellular pathways that may mediate insulin transport upon delivery by phosphonated MSN.

### 2.7 MSN Carriers Facilitate the Transport of Bioactive Insulin Across the Intestinal Epithelium

**FIGURE 6:**
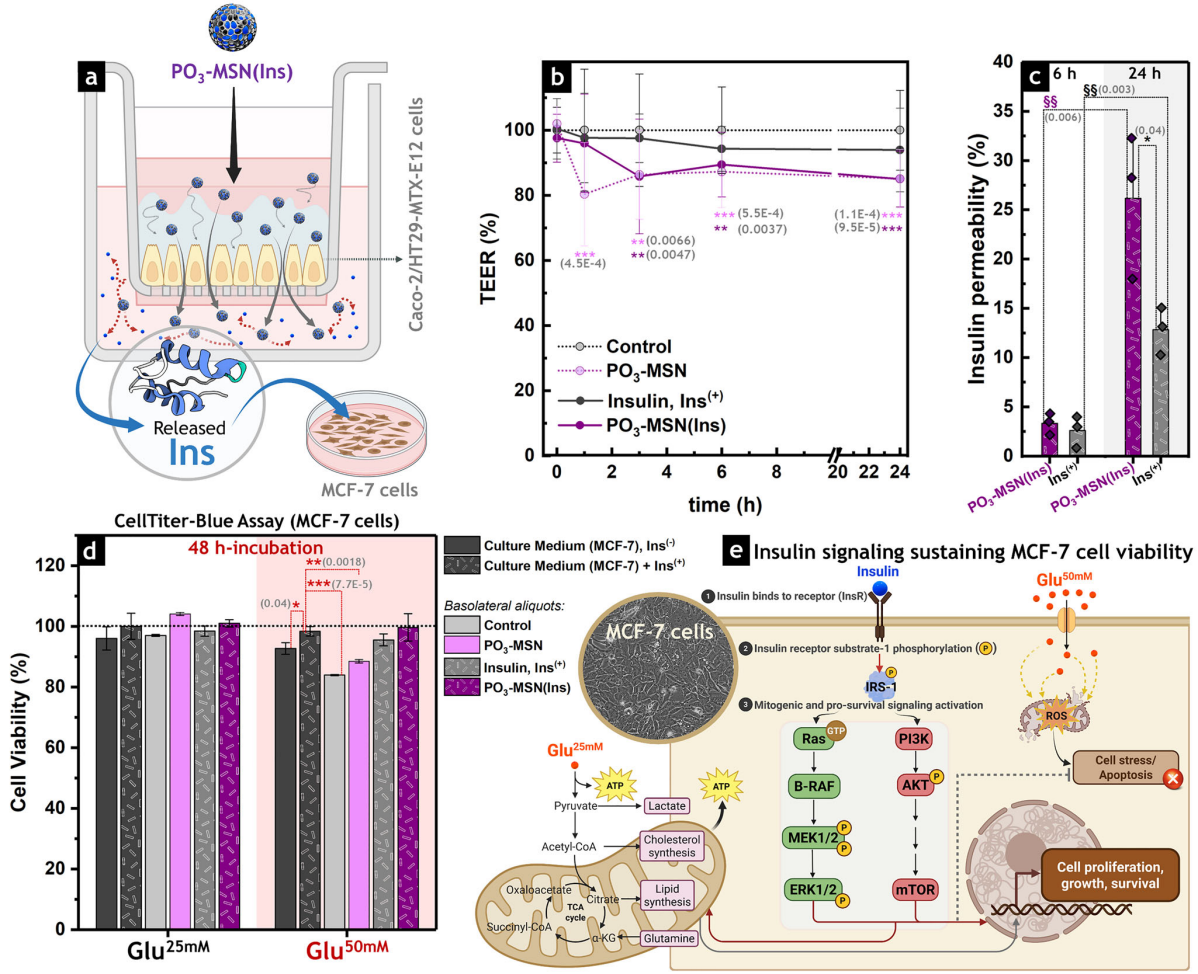
Paracellular transport of insulin‐loaded MSN across intestinal epithelial co‐cultures and effects of delivered insulin on cell viability. (a) Schematic representation of the cell culture insert system used for permeability studies (created with BioRender.com). Insulin‐loaded phosphonated MSN (**PO_3_‐MSN(Ins)**) were applied to the apical compartment, enabling their transport across the mucus‐covered intestinal Caco‐2/HT29‐MTX‐E12 cell monolayer to the basolateral side, where insulin was released. Aliquots collected from the basolateral compartment were subsequently applied to an established MCF‐7 breast cancer cell model to evaluate insulin bioactivity. (b) Transepithelial electrical resistance (TEER) of Caco‐2/HT29‐MTX‐E12 cells treated with **PO_3_‐MSN(Ins)**, empty **PO_3_‐MSN**, or non‐confined insulin (**Ins^(+)^**). TEER values were expressed as a percentage relative to the control (non‐treated cells) at each time point. The control consistently exhibited TEER values ranging from 200 to 250 Ω·cm^2^. Data sets are presented as mean ± standard deviation (*N* = 3; biological triplicates). One‐way ANOVA with Fisher's test confirmed significant differences between MSN‐treated groups (**PO_3_‐MSN(Ins)** or **PO_3_‐MSN**) and non‐treated control cells (** *p* < 0.01, *** *p* < 0.001). (c) Insulin permeability (%) after incubation of non‐confined insulin (**Ins^(+)^**) or **PO_3_‐MSN(Ins)** in the apical compartment of permeable cell culture inserts for 6 or 24 h, expressed as the relative concentration of insulin detected in the basolateral compartment compared with the total insulin applied to the apical suspension (100 µg·mL^−1^, 17 µM). Statistical significance was determined by one‐way ANOVA and Fisher's test, either between treatments at the same time point (*) or between time points for the same treatment (§), indicated as */§ (*p* < 0.05) or **/§§ (*p* < 0.01). Data sets are presented as mean ± standard deviation (*N* = 3; biological triplicates). (d) Cell viability (%) of MCF‐7 cells measured by CellTiter‐Blue (CTB) assay after 48 h‐incubation with aliquots collected from the basolateral compartment of permeable inserts previously incubated for 24 h with **PO_3_‐MSN(Ins)**, empty **PO_3_‐MSN**, or non‐confined insulin (**Ins^(+)^**), or from untreated Caco‐2/HT29‐MTX‐E12 cells (control). Treatments were combined with glucose at standard (25 mM, Glu^25mM^) or high (50 mM, Glu^50mM^) concentrations to assess the effects of glucose levels on cell viability and the ability of the delivered insulin to sustain metabolic activity and proliferation in MCF‐7 cells. Additional controls included MCF‐7 cells cultured in standard medium supplemented with insulin (MCF‐7 + **Ins^(+)^**, 0.1 µg·mL^−1^, 17 nM) or under starvation conditions (MCF‐7, **Ins^(−)^**). Data sets are shown as mean ± standard deviation (*N* = 3). Statistical significance was determined by one‐way ANOVA and Fisher's test (*p < 0.05, ** *p* < 0.01, *** *p* < 0.001). (e) Schematic representation of key steps in insulin‐signaling pathways (created with BioRender.com). Upon binding to the insulin receptor (InsR), insulin triggers phosphorylation of the insulin receptor substrate‐1 (IRS‐1), leading to activation of the phosphoinositide 3‐kinase/protein kinase B (PI3K/Akt) and extracellular signal‐regulated kinase 1/2 (ERK1/2) cascades. These pathways regulate cell survival, metabolism, and proliferation, stimulating mitochondrial activity and supporting cell cycle progression. Under normal glucose conditions (25 mM, Glu^25mM^), these mechanisms maintain cellular homeostasis and viability. Under high‐glucose conditions (50 mM, Glu^50mM^), insulin signaling counteracts oxidative and metabolic stress, thereby preserving mitochondrial function and preventing cell death [85].

To further investigate the transport of insulin across an intestinal cell monolayer, the interactions of **PO_3_‐MSN(Ins)** with a Caco‐2/HT29‐MTX‐E12 co‐culture were evaluated in permeable cell culture inserts (Figure 6a). This co‐culture model differentiates into functional monolayers that closely mimic key features of the gut barrier, including an apical brush border with microvilli, active electrolyte transport, and expression of TJ proteins [25, 81]. Compared with Caco‐2 monocultures, the addition of HT29‐MTX‐E12 cells creates a more physiologically relevant environment by secreting mucus, which establishes a protective diffusion barrier while maintaining a tight epithelial monolayer characterized by high transepithelial electrical resistance (TEER). This makes the model well‐suited to evaluating paracellular diffusion/uptake of bioactive molecules and assessing the barrier‐disrupting effects induced by food constituents, contaminants, or pathogens [82, 83, 84]. To ensure consistent mucus production and expression of TJ proteins, cells were differentiated for 14 days according to established protocols [25, 31, 84].

Among the MSN‐based formulations, **PO_3_‐MSN(Ins)** was selected for permeability studies due to its enhanced colloidal stability, improved insulin solubility, and controlled release profile in simulated gastrointestinal fluids (Figures 2e and S7a), as well as increased intestinal insulin availability in the HCEC‐1CT model when delivered via phosphonated MSN carriers (Figure 4d). The concentration of **PO_3_‐MSN(Ins)** used to treat the cell‐cultured inserts was set at 440 µg·mL^−1^ (corresponding to 100 µg·mL^−1^ of loaded insulin, 17 µM). Empty **PO_3_‐MSN** were included in the experimental layout at the same particle concentration as in the **PO_3_‐MSN(Ins)** treatment (340 µg·mL^−1^) to isolate the effects of the carrier on barrier modulation. This concentration was chosen based on previous reports indicating that MSN levels up to 600 µg·mL^−1^ preserve monolayer integrity without inducing cell detachment or loss of viability [25, 31]. Furthermore, free insulin (**Ins^(+)^**) was tested at the same concentration (100 µg·mL^−1^, 17 µM) as in the **PO_3_‐MSN(Ins)** treatment, and untreated Caco‐2/HT29‐MTX‐E12 cells served as controls.

Barrier integrity was initially evaluated by TEER measurements (Figure 6b). After 3 h of treatment with **PO_3_‐MSN(Ins)**, TEER decreased significantly by 14% (*p* = 0.0047) relative to the control (non‐treated cells), which maintained values between 200 and 250 Ω·cm^2^ throughout the assay, consistent with tight intestinal monolayers [82]. A similar effect was observed with **PO_3_‐MSN** treatment, triggering the steepest reduction within the first hour (to 80% of control) but subsequently stabilizing the TEER values at levels comparable to those measured in **PO_3_‐MSN(Ins)**‐treated cells, and showing a 14% decrease in TEER after 3, 6, and 24 h. In contrast, non‐confined **Ins^(+)^** produced only minor, non‐significant changes in TEER values of treated cells, suggesting that barrier modulation was driven by the MSN carriers rather than the insulin cargo.

To determine whether the decrease in TEER reflected barrier disruption, a Lucifer Yellow permeability assay was performed after 24 h of incubation with **PO_3_‐MSN(Ins)**, **PO_3_‐MSN**, or **Ins^(+)^** (Figure S20a). No significant increase in Lucifer Yellow flux was detected for any treatment compared with controls (non‐treated Caco‐2/HT29‐MTX‐E12 cells), which supported that MSN formulations induced transient barrier modulation without compromising overall monolayer integrity. These results align with previous studies, demonstrating that the mucus layer limits the extensive disruption of epithelial monolayers during nanoparticle exposure [25, 31]. A Lucifer Yellow assay in cell‐free inserts further validated this conclusion (Figure S20a, yellow background), as in the absence of the intestinal model, significantly higher fluorescence (*p* < 0.001) was detected in the basolateral compartment of permeable inserts compared with cell‐seeded systems. Lucifer Yellow quantification in this cell‐free setup (95% apical vs. 5% basolateral fluorescence relative to the applied stock solution) confirmed the maximal diffusion capacity of the dye across the permeable supports, thereby excluding membrane retention artifacts that could otherwise compromise accurate analysis in cell‐containing inserts exposed to the treatments (**PO_3_‐MSN(Ins)**, **PO_3_‐MSN**, or **Ins^(+)^**).

To directly assess insulin transport across the Caco‐2/HT29‐MTX‐E12 co‐culture, aliquots from the basolateral compartment of the permeable cell culture inserts were collected after 6 h and 24 h of incubation with **PO_3_‐MSN(Ins)** or free insulin (**Ins^(+)^**) and analyzed by HPLC–MS (Figure 6c). These time points were selected to capture both early and prolonged transport phases relevant to oral delivery applications [86, 87], and to align with the TEER measurements (Figure 6b). After 6 h, the insulin levels detected in the basolateral compartment were slightly higher in **PO_3_‐MSN(Ins)**‐treated inserts than in those incubated with free insulin (**Ins^(+)^**), corresponding to 3% of the total insulin applied to the apical side (Figure 6c), which correlated with the reduction in TEER observed at the same time point compared with controls (Figure 6b). Notably, after 24 h, a significantly higher insulin concentration (*p* = 0.04) was detected in the basolateral compartment of **PO_3_‐MSN(Ins)**‐treated inserts (26% of the initial dose) compared with **Ins^(+)^**, which reached only 13% of the initial concentration used for cell treatment (100 µg·mL^−1^, 17 µM). These findings strongly support that MSN carriers enhance the paracellular transport of insulin across the intestinal barrier (Figure 6c).

A similar trend was observed in cell‐free inserts (Figure S20b), where insulin levels in the basolateral compartment after 6 and 24 h were higher for **PO_3_‐MSN(Ins)** (14% and 40% of the initial dose, respectively) than for **Ins^(+)^** (9% and 35%, respectively), which supported the increased solubility and permeability of insulin when delivered by **PO_3_‐MSN**. These cell‐free controls distinguished the intrinsic contribution of the insert membrane from the barrier provided by the intestinal cell monolayers, thereby indicating the maximum fraction of insulin able to diffuse across the membrane. In contrast, the cell‐based experiments incorporated the additional physiological constraints imposed by the intestinal co‐culture and confirmed that the enhanced permeability observed for **PO_3_‐MSN(Ins)** was not attributable to passive insulin diffusion across the support, but to the ability of the **PO_3_‐MSN** carriers to promote controlled release and sustained paracellular transport across the intestinal epithelium, highlighting their potential for oral delivery applications.

The detection of insulin in the basolateral compartments of cell‐treated inserts by HPLC‐MS additionally confirmed the structural integrity of the peptide drug after contact with the intestinal monolayer. To further evaluate the bioactivity of insulin delivered by **PO_3_‐MSN(Ins)**, aliquots from the basolateral compartment of cell‐seeded inserts were applied to MCF‐7 cells (Figure 6a). This estrogen‐dependent breast cancer cell model was selected because its metabolic activity is tightly regulated by insulin, which supports cell growth and proliferation [85, 88]. Thus, the viability of MCF‐7 cells exposed to the basolateral media containing insulin delivered from **PO_3_‐MSN(Ins)** or from non‐confined insulin (**Ins^(+)^**) was assessed. Cell viability was quantified using the CellTiter‐Blue (CTB) assay (Figure 6d), which measures the metabolic activity of living cells by reducing resazurin to the fluorescent product resorufin [89]. Additional control treatments included basolateral medium from inserts treated with empty **PO_3_‐MSN**, non‐treated Caco‐2/HT29‐MTX‐E12 cells, as well as MCF‐7 cells maintained in standard culture medium supplemented with insulin (positive control, **Ins^(+)^** supplement, 0.1 µg·mL^−1^, 17 nM), and MCF‐7 cells incubated under insulin‐starvation conditions (negative control, **Ins^(−)^**). This layout ensured that the potential effects of the basolateral medium composition and contributions from empty nanoparticles could be distinguished from insulin‐specific responses.

All treatments were tested at a standard glucose concentration for cell culture (25 mM, Glu^25mM^) and at elevated levels (50 mM, Glu^50mM^). The latter simulates severe pathological hyperglycemia, which, depending on exposure time, increases glycolytic flux and activates growth‐promoting signaling cascades by phosphorylating protein kinase B (PKB/Akt), extracellular signal‐regulated kinase 1/2 (ERK1/2), and signal transducer and activator of transcription 3 (STAT3). These effects create a favorable environment for the aggressive migration and proliferation of MCF‐7 cells [85, 90, 91]. In addition, high glucose promotes breast cancer progression and metastasis by downregulating angiotensinogen expression [92] and can synergize with insulin to further amplify pro‐tumorigenic signaling [85]. Nevertheless, prolonged exposure to high‐glucose conditions can also induce cell death in MCF‐7 cells, in a dose‐ and time‐dependent manner that varies with the insulin concentration [85, 93]. This provided a pathophysiologically relevant stress model to evaluate the biological activity of delivered insulin.

Thus, after 48 h of incubation of the cells with the basolateral aliquots at Glu^25mM^, no significant differences in MCF‐7 viability were observed across the treatments (Figure 6d), including those lacking insulin supplementation (basolateral control medium, empty **PO_3_‐MSN**, or insulin‐starved MCF‐7 cells incubated in standard medium). These findings ruled out adverse effects of the altered medium composition and were consistent with the high resilience of MCF‐7 cells to culture stress [94], aligning with previous starvation studies in HCEC‐1CT cells (**Ins^(−)^**), which showed limited cell sensitivity to insulin deprivation under standard glucose concentrations for cell culture (Figure S10a). In contrast, under Glu^50mM^ conditions, only the treatments containing insulin, **PO_3_‐MSN(Ins)** or **Ins^(+)^**, maintained MCF‐7 viability at levels comparable to the controls (non‐treated MCF‐7 cells in standard medium, **Ins^(+)^**). In the absence of insulin, significant reductions in viability were observed in MCF‐7 cells exposed to basolateral control medium of Caco‐2/HT29‐MTX‐E12 cells (*p* = 7.7×10^−5^), empty **PO_3_‐MSN** (*p* = 0.0018), or insulin‐deprived medium (negative control, MCF‐7 cells, **Ins^(−)^**, *p* = 0.04), showing 15%, 11%, and 7% decreases, respectively, compared with the positive control (non‐treated MCF‐7 cells in standard medium, **Ins^(+)^**). These results confirmed that insulin remained biologically active after transport across the intestinal Caco‐2/HT29‐MTX‐E12 co‐culture, thereby sustaining MCF‐7 cell viability when delivered by **PO_3_‐MSN(Ins)**. At high glucose concentrations (Glu^50mM^), excessive glucose availability can induce oxidative and metabolic stress, leading to mitochondrial dysfunction and apoptotic signaling in MCF‐7 cells [95, 96]. The presence of insulin can counteract these effects by activating pro‐survival signaling such as the phosphoinositide 3‐kinase/protein kinase B (PI3K/Akt) and the ERK1/2 pathways [85, 97, 98]. Consequently, insulin acts as a key pro‐survival factor, enabling MCF‐7 cells to tolerate hyperglycemic stress and possibly sustain energy production required for proliferation (Figure 6e).

**FIGURE 7:**
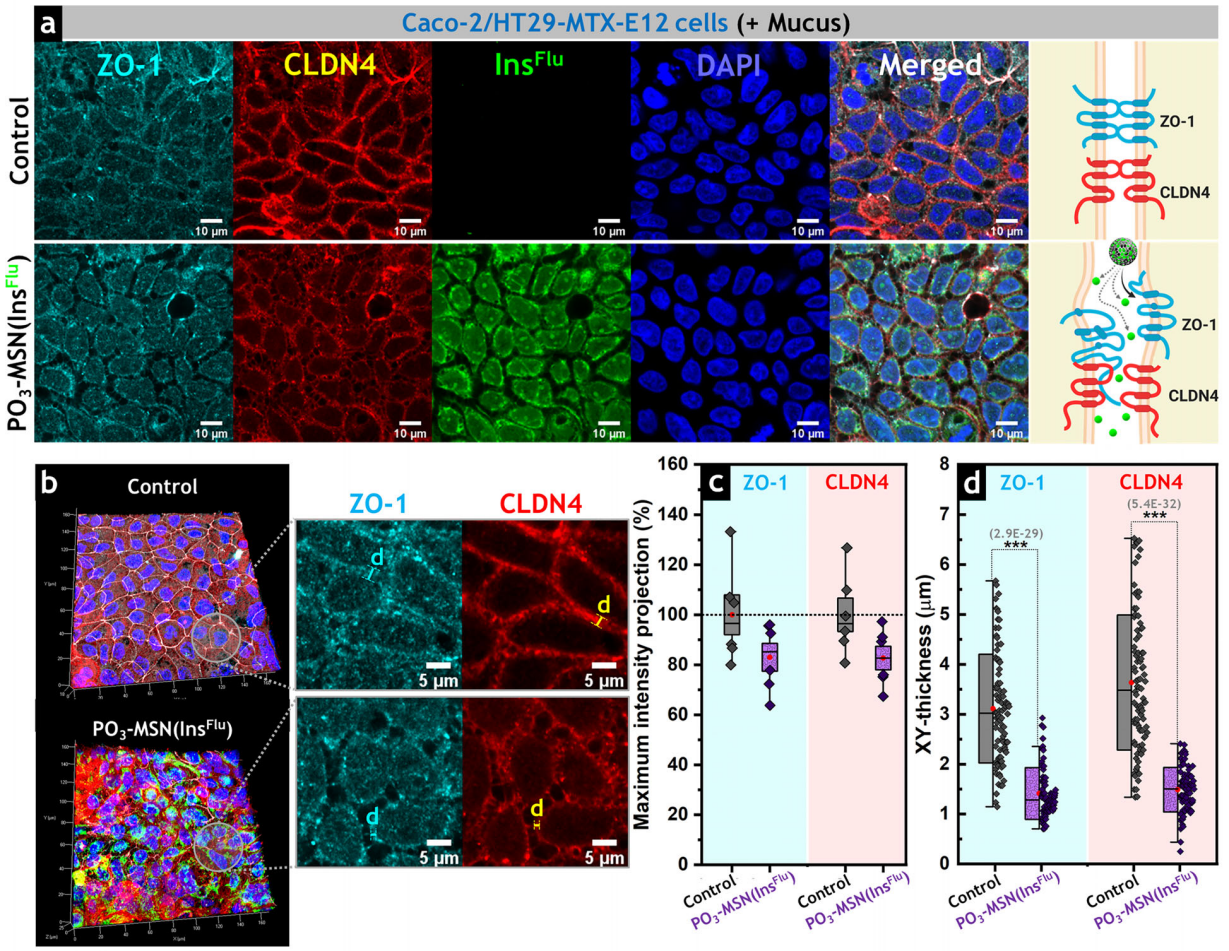
Modulation of tight junctions (TJs) by phosphonated MSN and distribution of insulin delivered *via* particle formulations. (a) Representative images of the immunofluorescence staining of TJ proteins in Caco‐2/HT29‐MTX‐E12 cells after 6 h treatment with **PO_3_‐MSN(Ins^Flu^)**. Control corresponds to non‐treated cells incubated in complete culture medium. ZO‐1 is shown in cyan, CLDN4 in red, **Ins^Flu^** in green, and nuclei (stained with DAPI) in blue. Scale bars stand for 10 µm. Schematic representations of ZO‐1 and CLDN4 distributions were created with BioRender.com. (b) Representative 3D reconstructions (63× magnification) showing ZO‐1 (cyan), CLDN4 (red), and **Ins^Flu^** (green), obtained by z‐stack imaging. Nuclei are stained with DAPI (blue). The scale bar segmentation is 10 µm. XY‐thickness (d, µm) of ZO‐1 and CLDN4 were derived from zoomed‐in stacks aligned to the nuclear layer (reference). The scale bars stand for 5 µm. (c) Quantification of the mean fluorescence intensity of ZO‐1 and CLDN4 from maximum‐intensity projection images obtained from 3D reconstructions (*n* = 6 images derived from independent biological triplicates, including technical duplicates). (d) Quantification of XY‐thickness (d, µm) of ZO‐1 and CLDN4 based on the analysis of more than 90 cells (*n* ≥ 90) from three independent biological replicates (*N* = 3), including technical duplicates. Data sets are presented as box plots, with the mean (red dot) and standard deviation shown. Statistically significant differences, determined by one‐way ANOVA and Fisher's test, between **PO_3_‐MSN(Ins^Flu^)**‐treated and control (non‐treated) cells are indicated as *** *p* < 0.001.

The plausibility of the transport across intestinal cells for the **PO_3_‐MSN** carrier and respective modulation of barrier function was further assessed by monitoring the distribution of Zonula Occludens‐1 (ZO‐1) and Claudin‐4 (CLDN4) in Caco‐2/HT29‐MTX‐E12 cells (Figure 7). ZO‐1 is a scaffolding protein that links transmembrane and cytoskeletal components to the actin cytoskeleton and is essential for maintaining barrier integrity [99]. On the other hand, CLDN4 is a transmembrane protein of the claudin family that contributes to the formation of TJ strands and the regulation of paracellular permeability [99, 100]. Both proteins are widely recognized markers of intestinal epithelial integrity, as their expression and distribution reliably indicate barrier modulation [31, 84].

The experimental setup was adapted from a previous study, in which exposure to phosphonated MSN induced the redistribution of both ZO‐1 and CLDN4 after 6 h of treatment; the hydrophilic and negatively charged THMP functions grafted on the MSN surface further minimized particle–mucin interactions, thereby facilitating transit through the mucus layer and promoting enhanced interactions with intestinal cells [31]. Building on this evidence, Caco‐2/HT29‐MTX‐E12 cells were treated with insulin‐loaded MSN at the same concentration used in the permeability studies (100 µg·mL^−1^ of insulin, 17 µM). However, for these experiments, FITC‐labeled insulin (**Ins^Flu^**, Figure S12) was loaded into **PO_3_‐MSN** to enable visualization of insulin distribution across the cell monolayer upon delivery from the labeled material (**PO_3_‐MSN(Ins^Flu^)**, Figure 7).

**Ins^Flu^** was loaded into the phosphonated MSN (**PO_3_‐MSN(Ins^Flu^)**) using the same protocol performed for the non‐fluorescent materials. The incorporation of 27.8 wt% of insulin into the silica pores was confirmed from the mass loss detected by TGA, which was attributed to the thermal decomposition of the loaded **Ins^Flu^** (Figure S3f, Table S1).

After 14 days of differentiation, Caco‐2/HT29‐MTX‐E12 cells were incubated with **PO_3_‐MSN(Ins^Flu^)** for 6 h and subsequently stained to visualize ZO‐1 and CLDN4 expression [31, 84]. In control (non‐treated) cells, both TJ proteins displayed a well‐organized network typical of a tight epithelial monolayer (Figure 7a,b) [84]. Fluorescence signals of ZO‐1 and CLDN4 were quantified from maximum intensity projections of 3D reconstructions (Figure S21a), revealing only a slight, non‐significant decrease after **PO_3_‐MSN(Ins^Flu^)** treatment compared with controls (Figure 7c). These results indicated that overall barrier integrity was preserved in the presence of MSN, consistent with previous reports [25, 31].

To further assess TJ modulation induced by particle treatments, the XY‐thickness of the fluorescence signals in the 2D optical fields was quantified, using the nuclei as a reference for focal‐plane selection (Figure 7d) [31]. This analysis confirmed a significant decrease in ZO‐1 thickness (from 3.1 to 1.4 µm, *p* = 2.9×10^−29^) and in CLDN4 thickness (from 3.6 to 1.5 µm, *p* = 5.4×10^−32^) in cells treated with **PO_3_‐MSN(Ins^Flu^)**, compared with controls. The reduction in ZO‐1 and CLDN4 staining thickness was also visualized in magnified stacks obtained from 3D reconstructions at the nuclear plane (Figure 7b, zoom‐in images). Such redistribution of TJ proteins suggested a loosening of the cell‐cell contacts, thereby easing **PO_3_‐MSN(Ins^Flu^)** transport (Figure 6a), which correlated with a decrease in TEER values in cells treated with **PO_3_‐MSN(Ins)** after 6 h, compared with non‐treated control cells (Figure 6b).

The 3D reconstructions obtained from *z*‐stack imaging further confirmed the distribution of **Ins^Flu^** throughout the cell monolayer (Figure S21a). Higher‐magnification 2D images revealed insulin accumulation not only at the cell membrane but also within the cytoplasm (Figure 7a), indicating cellular uptake. This aligns with the lower insulin concentration detected on the basolateral side after 6 h of incubation with **PO_3_‐MSN(Ins)**, compared to the higher levels observed after 24 h (Figure 6c), as part of the internalized insulin initially might have remained within the cells. After 24 h of incubation, basolateral insulin concentrations increased, consistent with gradual release following intracellular trafficking [101]. Alternatively, the lower insulin levels observed after 6 h of incubation could be attributed to the incomplete release of **Ins^Flu^** from the **PO_3_‐MSN** carriers in the basolateral compartment, supporting the interpretation that **PO_3_‐MSN(Ins^Flu^)** are transported through the paracellular space and exhibit a time‐dependent controlled release. This is consistent with the increased insulin levels in the basolateral compartment in cell‐free permeable inserts after 24 h of incubation with **PO_3_‐MSN(Ins)** compared to 6 h (Figure S20b).

To further explore MSN distribution across the cell monolayer, cells were incubated with empty **PO_3_‐MSN** as an additional control experiment. For this purpose, the phosphonated MSN were labeled with FITC following a previously reported protocol (Scheme S2) [31], and the fluorescent particles (**PO_3_‐MSN^Flu^**) were applied to the Caco‐2/HT29‐MTX‐E12 co‐culture at the same particle concentration as in the **PO_3_‐MSN(Ins^Flu^)** treatment (260 µg·mL^−1^). This approach enabled the isolation of particle‐specific effects on TJ protein redistribution.

The hydrodynamic diameter (161 ± 1 nm) and zeta potential (−37 mV) of **PO_3_‐MSN^Flu^** did not significantly change compared with the non‐fluorescent **PO_3_‐MSN** used as insulin carriers (Figure S1c, Table S1), which was essential to maintain particle diffusion through the mucus layer and subsequent interactions with intestinal cells. Furthermore, TGA confirmed the conjugation of 3.1 wt% fluorescein after MSN labeling (Figure S3c, Table S1), which was suitable for the detection of the fluorescence signal from **PO_3_‐MSN^Flu^** after 6 h of incubation with the Caco‐2/HT29‐MTX‐E12 co‐culture. Although 3D reconstructions obtained from Z‐stack imaging confirmed the interaction of **PO_3_‐MSN^Flu^** with intestinal cells, supporting their ability to diffuse through the mucus layer (Figure S21a), analysis of orthogonal views revealed that the MSN were not internalized by the cells but were instead localized at the cell surface, in association with TJ proteins (Figure S21b). This observation rather ruled out particle uptake and supported the potential transport of **PO_3_‐MSN(Ins)** through the paracellular pathway to the basolateral compartment of cell‐seeded permeable inserts, allowing for controlled insulin release (Figure 6a).

Quantification of the fluorescent signals of CLDN4 and ZO‐1 from maximum‐intensity projections of the 3D reconstructions (Figure S21c) showed no significant differences compared with the control (non‐treated cells), indicating that monolayer integrity was preserved upon **PO_3_‐MSN^Flu^** treatment, as observed for **PO_3_‐MSN(Ins^Flu^)** (Figure 7c). Furthermore, analysis of the XY‐thickness of TJ stainings in the 2D optical fields (Figure S21d) revealed that treatment with empty **PO_3_‐MSN^Flu^** induced the same response as **PO_3_‐MSN(Ins^Flu^)** (Figure 7d), leading to a significant reduction in the XY‐thickness of both ZO‐1 and CLDN4 (1.5 µm each, *p* < 0.001) compared to the control (non‐treated cells showing values of 3.1 and 3.6 µm for ZO‐1 and CLDN‐4, respectively). This is consistent with a previous report showing that phosphonated MSN increase cell–cell distances and loosen TJs without compromising cell viability [31]. Overall, these results corroborated that the redistribution of TJ proteins observed upon **PO_3_‐MSN(Ins^Flu^)** treatment (Figure 7) was due to the **PO_3_‐MSN** carriers, supporting their potential to modulate intestinal barrier function. These findings also align with the observed reduction in TEER (Figure 6b) and enhanced insulin permeability (Figure 6c), supporting that **PO_3_‐MSN(Ins)** can transiently reorganize TJs and promote insulin transport across intestinal cell monolayers while retaining bioactivity upon release (Figure 6d).

### 2.8 MSN‐Based Formulations Decrease Blood Glucose via Insulin Delivery

**FIGURE 8:**
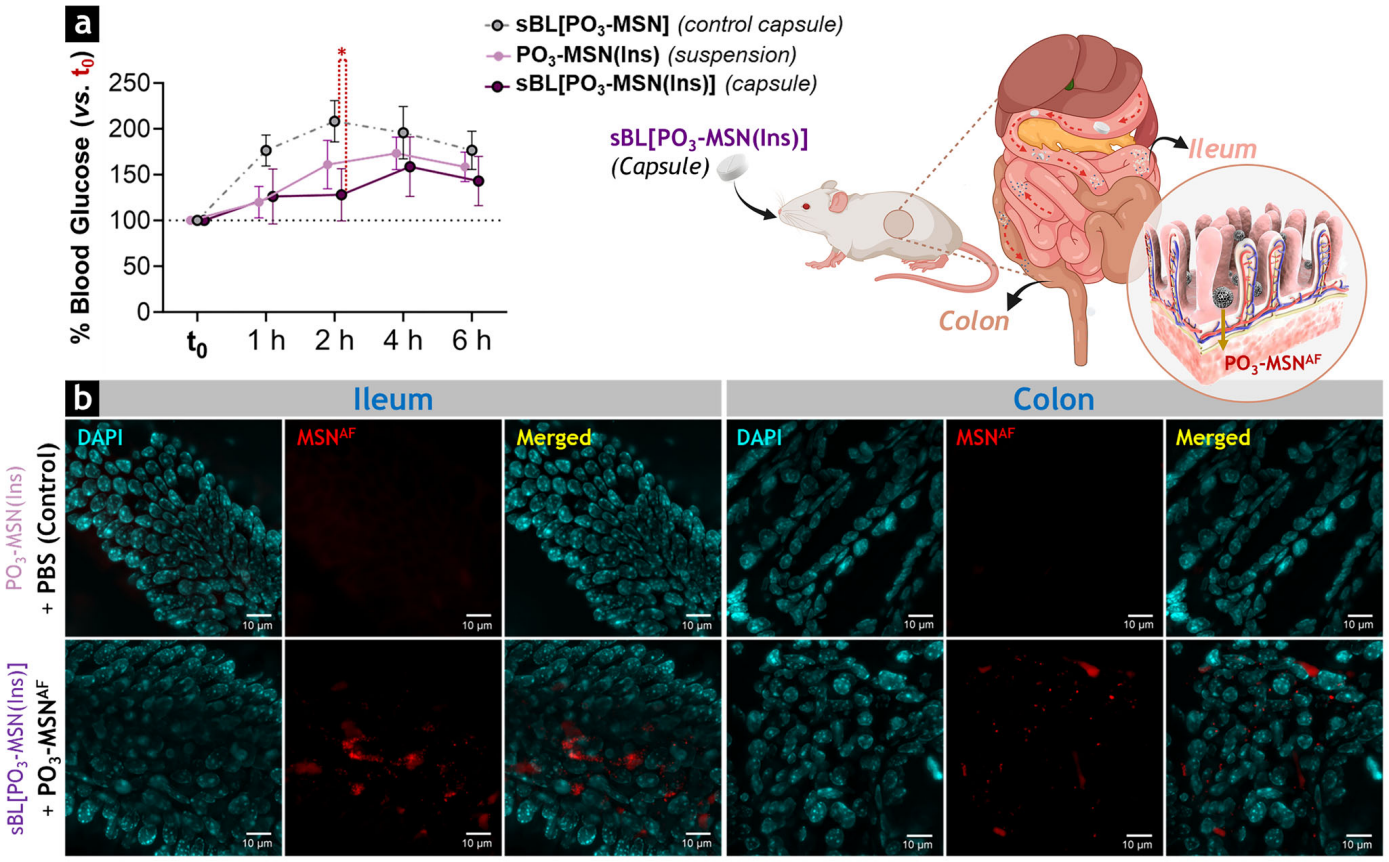
In vivo insulin tolerance and distribution of MSN in the intestinal tract of diabetic mice. (a) Blood glucose levels in fasted streptozotocin (STZ)‐induced diabetic mice following oral gavage with empty phosphonated particles (**sBL[PO_3_‐MSN**], control capsule; dashed grey line), insulin‐loaded MSN in capsule format (**sBL[PO_3_‐MSN(Ins)**], dark purple), or insulin‐loaded MSN administered as a PBS suspension (**PO_3_‐MSN(Ins)**, light purple). Data are presented as mean ± standard deviation (*n* = 5 mice per group). One‐way ANOVA with Fisher's test indicated significant differences between **sBL[PO_3_‐MSN(Ins)**] and the control **sBL[PO_3_‐MSN**] at 2 h post‐gavage (*p = 0.04101). (b) Representative epifluorescence microscopy images of ileum and colon sections from mice administered **sBL[PO_3_‐MSN(Ins)**], followed by oral gavage with Alexa Fluor 647–labeled MSN suspension (**PO_3_‐MSN^AF^**). The control group consisted of mice receiving a **PO_3_‐MSN(Ins)** suspension in PBS containing dispersed sBL, followed by an additional PBS gavage. The graphical representation of capsule administration in mice, including its transit through the gastrointestinal tract, as well as the interaction of **PO_3_‐MSN^AF^** with intestinal cells, was created with BioRender.com.

After confirming that **PO_3_‐MSN** carriers enabled insulin to cross the intestinal epithelial barrier with preserved bioactivity in vitro, a validation experiment was performed in vivo. **PO_3_‐MSN(Ins)** were combined with sBL into mouse‐specific gel capsules (Size M) containing an insulin dose of 400 U·kg^−1^ (2.39 µM·kg^−1^), within a working concentration range previously reported [24]. In addition to the capsule formulation, the **PO_3_‐MSN(Ins)** were suspended in PBS to be administered by oral gavage (Figure 8). For control purposes, capsules containing empty phosphonated nanoparticles (**PO_3_‐MSN**) were additionally prepared. An equivalent amount of the sBL excipient was used in all three tested groups (**sBL[PO_3_‐MSN(Ins)]**‐capsule, **PO_3_‐MSN(Ins)**‐suspension in PBS containing sBL, or **sBL[PO_3_‐MSN]**‐control capsule). Hyperglycemia was induced by administering streptozotocin to C57BL/6J mice to ensure non‐fasted blood glucose levels higher than 250 mg·dL^−1^ (13.9 mmol·L^−1^) prior to insulin tolerance testing. The diabetic mice were fasted overnight for 12 h, an extended period to ensure an empty gastrointestinal tract, and blood glucose levels in treated mice were monitored hourly after oral gavage [24].

Glucose increased in all groups, despite insulin typically lowering glucose levels below baseline (Figure 8a) [24]. This response likely reflects stress associated with fasting and repeated gavage, which are known to stimulate hepatic glucose mobilization [102]. Nevertheless, a significant reduction in blood glucose levels was observed in mice treated with **sBL[PO_3_‐MSN(Ins)]** capsules compared with the control group (**sBL[PO_3_‐MSN]**, *p* = 0.04101) after 2 h of oral gavage. Although preliminary, these results are promising and support a therapeutic scope of the developed oral formulations.

Following administration of the **sBL[PO_3_‐MSN(Ins)]** capsules, mice received a second oral gavage of Alexa Fluor^TM^ 647 (AF)–labeled MSN (**PO_3_‐MSN^AF^**, 100 mg·kg^−1^ in PBS), while the other groups (**PO_3_‐MSN(Ins)‐**suspension or **sBL[PO_3_‐MSN]**‐control capsule) received a PBS gavage only. This procedure enabled the assessment of **PO_3_‐MSN** distribution in the gastrointestinal tract and eventually the observation of histopathological effects. For this purpose, **PO_3_‐MSN** were previously labeled with the succinimidyl ester of the AF fluorophore (Scheme S4, **PO_3_‐MSN^AF^**). The hydrodynamic diameter (165 ± 1 nm) and zeta potential (−38 mV) of **PO_3_‐MSN^AF^** did not change significantly compared to the precursor sample **PO_3_‐MSN** (164 ± 1 nm and −38 mV, respectively), confirming minimal alterations in the particle size and surface chemistry of the labeled material (Figure S1c, Table S1). This was consistent with the incorporation of only 2 wt.% AF after MSN labeling, as determined by TGA (Figure S3c), which was still sufficient to enable efficient visualization of the fluorescence signal of **PO_3_‐MSN^AF^** (Figure 8b). Thus, after glucose measurements, the mice were euthanized, and tissue samples were collected for further analysis. Epifluorescence microscopy of intestinal cryosections confirmed the presence of **PO_3_‐MSN^AF^** throughout the colonic and ileal epithelium of mice administered insulin‐loaded capsules (**sBL[PO_3_‐MSN(Ins)]**) followed by **PO_3_‐MSN^AF^** gavage in PBS. In contrast, no fluorescence was detected in colon or ileum tissues of control mice that received **PO_3_‐MSN(Ins)** and sBL suspended in PBS, followed by additional PBS gavage (Figure 8b). Although further optimization of the experimental setup is required to normalize particle concentrations across treatment groups, these results provide valuable preliminary insights into the MSN biodistribution and meaningfully support the predictive value of the in vitro test batteries. Notably, the close agreement between in vitro and in vivo observations underscores the robustness of the cell culture models and their potential to guide experimental design and limit the need for extensive animal testing.

Aligning with the absence of cytotoxicity observed during the in vitro experiments, histopathological examination of the ileum and colon from mice administered **sBL[PO_3_‐MSN(Ins)]** capsules and additional **PO_3_‐MSN^AF^** gavage revealed no pathological alterations (Figure S22). Although further studies are needed to validate the safety and efficacy of the formulations, data collected so far support reduced toxicological potential, which is a fundamental prerequisite for further translational development of MSN‐based formulations, including the potential application for other peptide‐based therapeutics.

## 3 Conclusions

Recent advances in materials science have led to nanoparticle formulations capable of overcoming key gastrointestinal barriers, creating new opportunities for the oral delivery of peptide drugs and biologics. However, their application still requires improvements in safety, efficacy, and bioavailability before clinical translation can be achieved. In this study, we used insulin as a model peptide and loaded it into MSN integrated into pH‐responsive tablet formulations as a promising noninvasive strategy for oral delivery.

The modification of MSN with PEG and phosphonate functions enhanced their colloidal stability across a wide pH range while preserving sufficient porosity for insulin loading *via* physical adsorption. Insulin confinement within the MSN mesopores induced a crystalline‐to‐amorphous transition of insulin's physical state, increasing its solubility by ∼2.5‐fold. Insulin encapsulation within MSN, together with the inclusion of sBL as a protective protein excipient in the tablet formulations, was crucial for preventing premature insulin release under gastric conditions, minimizing proteolytic degradation, and enabling controlled intestinal delivery consistent with diffusion‐governed kinetics, while preserving the native insulin conformation required for its biological activity. These engineered formulations prolonged insulin stability in simulated digestive fluids, extending its half‐life from 2 min for non‐confined insulin to 3.5 h for the MSN‐containing tablets, which exhibited a pH‐responsive release profile aligning with the physiological conditions of gastrointestinal transit.

MSN functionalization with phosphonate moieties further enhanced particle uptake by intestinal epithelial cells and efficient insulin availability and overall low cytotoxicity. These results highlight a novel strategy for the transcellular delivery of peptide therapeutics. Internalization and bioactivity of insulin, used here as a case study, were validated using complementary analytical approaches, which collectively demonstrated robust activation of insulin signaling pathways and sustained cellular viability under metabolic stress, such as hyperglycemic or insulin‐deprived conditions.

In addition to enhanced transcellular delivery, phosphonated MSN enabled particle diffusion through the mucus layer and transiently modulated epithelial barrier function via redistribution of TJ proteins, thereby facilitating paracellular permeability of insulin‐loaded MSN. Together, these results highlight the versatility of MSN carriers to engage both transcellular and paracellular transport pathways and underscore the possibility of tailoring oral formulations to the specific peptide cargo and therapeutic application.

Preliminary in vivo studies with insulin‐loaded MSN‐based tablets revealed a trend toward reduced blood glucose levels without causing intestinal damage. Although further optimization of administration regimens and detailed evaluation of particle transit, uptake, and biodistribution are still required, these findings closely align with the in vitro profiling results, underscoring the robustness and predictive value of the advanced cell culture models applied. These results highlight the utility of in vitro platforms for systematic screening and optimization of engineered oral formulations, enabling a more targeted use of in vivo studies while minimizing animal experimentation. Altogether, this MSN‐based delivery approach provides a transferable framework that may be extended to other peptides and biologics, with the potential to improve the oral bioavailability of gastro‐sensitive biomolecules.

## 4 Methods

Reagents and detailed descriptions of the techniques implemented for materials characterization are available in the Supporting Information.

### 4.1 Synthesis and Functionalization of Mesoporous Silica Nanoparticles

**MSN** synthesis was performed by adapting a published procedure [32]. Further experimental details can be found in the Supporting Information. **MSN** were functionalized with a PEG‐silane (2 kDa) via a general post‐grafting protocol [31, 40]. After overnight degassing at 150°C, 200 mg of calcined **MSN** were dispersed in 80 mL of anhydrous toluene at 110°C under stirring (600 rpm) and argon atmosphere. The PEG‐silane (25 mg) was then dissolved in anhydrous toluene under an argon atmosphere and added to the silica dispersion. The reaction medium was stirred overnight (600 rpm) at 110°C. The PEGylated‐MSN (**PEG‐MSN**) were isolated by centrifugation (9000 rpm, 11 770 × *g*, 20 min), washed once with toluene, twice with ethanol, and dried at 40°C overnight. The calcined **MSN** were also functionalized with phosphonate groups (**PO_3_‐MSN**) [31, 33]. First, THMP (3.3 mL, 16 mmol·g^−1^ of silica) was dissolved in water (100 mL), and the pH of the solution (pH = 10.9) was adjusted to pH 5 with 0.1 M HCl to avoid silica hydroxylation and dissolution during the grafting reaction. The THMP solution was added to the calcined **MSN** (800 mg), which was previously dispersed in 100 mL of distilled water. The reaction was kept under reflux at 100°C overnight. **PO_3_‐MSN** were isolated by centrifugation (9000 rpm, 11 770 ×* g*, 20 min) and washed once with water and three times with ethanol before being dried overnight at 40°C.

### 4.2 Insulin Loading

Insulin was introduced into the pores of MSN through physical adsorption [32]. **MSN**, **PEG‐MSN,** or **PO_3_‐MSN** were dispersed in 5 mL of nanopure H_2_O at pH 4. In parallel, insulin (40 mg) was dispersed in nanopure H_2_O (20 mL), and the pH was adjusted to 3 with 0.01 M HCl until it was completely dissolved. Afterward, the pH was raised to 4 using 0.2 M NaOH, and the insulin solution was added to the particle suspensions. The same procedure was used for the introduction of **Ins^Flu^** into the pores of rhodamine‐labeled particles (**MSN^Rhod^(Ins^Flu^)**, **PEG‐MSN^Rhod^(Ins^Flu^)**, or **PO_3_‐MSN^Rhod^(Ins^Flu^)**), **Ins^Rhod^** into the pores of FITC‐labeled particles (**MSN^Flu^(Ins^Rhod^)**, **PEG‐MSN^Flu^(Ins^Rhod^)**, or **PO_3_‐MSN^Flu^(Ins^Rhod^)**) or **Ins^Flu^** into the pores of phosphonated particles (**PO_3_‐MSN(Ins^Flu^)**). For each combination, 2 mg of labeled insulin (**Ins^Flu^** or **Ins^Rhod^**) were dispersed in 1 mL of nanopure H_2_O and mixed with 4 mg of **MSN**, **PEG‐MSN**, or **PO_3_‐MSN** (correspondingly labeled with Rhod‐ITC or FITC), previously dispersed in 250 µL of H_2_O at pH 4. The mixtures were placed in a mechanical rocker (80 rpm) for 2 h at room temperature (25°C). The resulting insulin‐loaded samples (**MSN(Ins)**, **PEG‐MSN(Ins)**, **PO_3_‐MSN(Ins)**, and fluorescent‐loaded samples) were recovered by centrifugation (9000 rpm, 11 770 × *g*, 10 min), frozen overnight (−20°C), and lyophilized for 2 days. Details on the labeling of MSN and insulin are provided in the Supporting Information.

### 4.3 Tablet Formulations

Tablets of sBL combined with either non‐confined insulin (**sBL[Ins]**, control) or insulin‐loaded MSN (**sBL[MSN(Ins)]**, **sBL[PEG‐MSN(Ins)]**, or **sBL[PO_3_‐MSN(Ins)]**) were prepared using a single punch press (PerkinElmer, UK). Prior to compression, 1.2 mg of insulin (equivalent to 6 mg of MSN containing 20 wt% of loaded insulin) was combined with 114 mg of sBL. The mixtures were placed in the press holder (6 mm diameter) and further compressed at 10 MPa for 2 min.

### 4.4 Insulin Release Experiments

The release of insulin from tablet formulations was tested in two buffers at pH 1.2 or 7.4 (control conditions) [32]. For release tests, 100 mL of these buffers was placed inside an incubator at 37°C and stirred at 150 rpm. The tablets were first soaked for 2 h at pH 1.2 and then were directly immersed in 5 mL of PBS for 3 min before being transferred to the pH 7.4 buffer to continue the test for 24 h. Aliquots of 100 µL were withdrawn at predetermined intervals and replaced with an equal volume of fresh solution to maintain a constant volume. The insulin concentration in these aliquots was quantified using an ultrahigh‐performance liquid chromatography‐mass spectrometry (UHPLC–MS) system. Release studies were also performed with insulin‐loaded MSN in the absence of sBL. For these experiments, no tablet preparation was required. Instead, 2 mg of insulin‐loaded MSN powders (**MSN(Ins)**, **PEG‐MSN(Ins)**, or **PO_3_‐MSN(Ins)**) or non‐encapsulated insulin (control) were suspended in 20 mL of buffer solutions at pH 1.2 or 7.4, ensuring an equivalent total insulin concentration of 20 µg·mL^−1^ (3.4 µM). The suspensions were incubated at 37°C under gentle stirring (80 rpm) in a mechanical shaker. Aliquots of 100 µL were collected at predetermined time points over 24 h and replaced with fresh buffer at the corresponding pH (100 µL), following the same procedure as for the tablet experiments. These samples were filtered using Nalgene Syringe Filters (0.2 µm pore size, 13 mm diameter) to remove residual particles and were subsequently analyzed by HPLC–MS. After 24 h of incubation in the buffer at pH 1.2 or 7.4, the resulting particle suspensions were characterized by TEM to assess the long‐term stability of the MSN carriers under simulated gastrointestinal conditions. Additionally, the experiments were repeated with an increased initial insulin concentration (200 µg·mL^−1^, 34 µM), and aliquots obtained after 2 h incubation at pH 7.4 were analyzed by CD spectroscopy to evaluate the integrity, solution‐state conformation, and secondary structure of the released insulin. The CD spectra of sBL (50% succinylation) used for tablet preparation, as well as those of the native protein (BL, 0% succinylation), were additionally recorded in buffers at pH 1.2 and pH 7.4, with all samples adjusted to a protein concentration of 200 µg·mL^−1^. Details on buffer preparation and UHPLC/HPLC–MS, TEM, and CD measurements are provided in the Supporting Information.

### 4.5 Insulin Stability in the Presence of Digestive Enzymes

The stability of insulin in tablet formulations was tested in SGF and SIF, which contained digestive enzymes [47]. First, the tablets were soaked in SGF (100 mL) for 1 or 2 h, then removed and placed in 5 mL of MeOH for 3 min before being transferred to 100 mL of buffer at pH 7.4 (same buffer used for release tests under control conditions). For stability tests in SIF, the tablets were soaked in SGF (100 mL) for 2 h, and then the pepsin was quenched by placing the tablets in MeOH (5 mL) for 3 min, as described above. After that, the tablets were transferred to SIF (100 mL). At predetermined time intervals (1, 2, or 3 h), the tablets were removed, placed in a quenching solution of trifluoroacetic acid (5% v/v in H_2_O) for 5 min, and transferred into 100 mL of the buffer at pH 7.4. After complete dissolution at pH 7.4, 1 mL aliquots were collected to quantify the remaining insulin by UHPLC‐MS. The stability of the non‐confined insulin (control) was additionally evaluated following the same procedure. Furthermore, dissolution testing of sBL‐tablets (without insulin and particles) was performed. See the Supporting Information for more details.

### 4.6 Cell Culture

Non‐tumorigenic human colonic epithelial cells (HCEC‐1CT) were kindly provided by Prof. Jerry W. Shay (UT Southwestern Medical Center, Dallas, TX, USA). Cell culture was performed according to a previously described protocol [70, 103] using Dulbecco's Modified Eagle Medium 92.8% (DMEM, Gibco, REF 21063‐029), supplemented with 2% medium 199 (10X), 2% cosmic calf serum, HEPES (20 mM), gentamycin (50 µg mL^−1^), insulin/transferrin/selenium‐G (10 µg mL^−1^), recombinant human EGF (20 ng mL^−1^), and hydrocortisone (1 µg mL^−1^). Caco‐2 cells, HT29‐MTX‐E12 cells, and MCF‐7 breast cancer cells were purchased from ATCC (Manassas, Virginia, USA) and cultivated in DMEM (high glucose, 4.5 g·L^−1^) supplemented with fetal bovine serum (FBS, 10%) and penicillin‐streptomycin (1%). For Caco‐2 and HT29‐MTX‐E12 cells, the cell culture medium additionally contained L‐glutamine (1%, 2 mM) and non‐essential amino acids (1%) [25, 31]. For MCF‐7 cells culture, bovine insulin supplement (0.1 µg·mL^−1^, 17 nM) was added to the culture medium [104]. The cells were incubated in a humidified incubator with 5% CO_2_ at 37°C.

### 4.7 Cell Treatment and Starvation Experiments in HCEC‐1CT Cells

Cell viability of the HCEC‐1CT model was assessed after incubation in insulin‐free medium (starvation conditions, **Ins^(−)^**). With this purpose, 10 000 cells (200 µL/well) were seeded in a 96‐well black‐sided plate (Sarstedt) at 37°C. After 48 h, cell confluency was observed, and the culture medium was removed and replaced by 200 µL of medium lacking insulin/transferrin/selenium‐G (insulin‐free medium, **Ins^(−)^**). The cells were further incubated under such insulin‐starvation conditions (**Ins^(−)^**) at 37°C for 1, 3, 6, and 24 h. For comparison, the experiments were also performed under standard culturing conditions (incubation with complete cell culture medium containing insulin, **Ins^(+)^**, 10 µg·mL^−1^, 1.7 µM). The viability of HCEC‐1CT cells incubated under starvation or standard culture conditions at each time point was correlated with cell proliferation using the WST‐1 assay. This first WST‐1 assay was followed by the treatment with insulin, both with the non‐confined commercial insulin (positive control, **Ins^(+)^**) or with the insulin‐loaded MSN (**MSN(Ins)**, **PEG‐MSN(Ins)**, or **PO_3_‐MSN(Ins)**) at an equivalent insulin concentration of 10 µg mL^−1^ (1.7 µM). The cells were additionally treated with empty particles (**MSN**, **PEG‐MSN**, or **PO_3_‐MSN**), which were dispersed in a complete cell culture medium (**Ins^(+)^**, 10 µg mL^−1^, 1.7 µM). Before the treatments, the samples were vortexed for 30 s and sonicated for 5 min to ensure dispersion in the corresponding media. Particle suspensions, complete medium (**Ins^(+)^**), or insulin‐free medium (**Ins^(‐)^**) were added to the cells (200 µL/well) and incubated for 3 h at 37°C. Cell viability after particle treatments was measured by a second WST‐1 assay, followed by a single washing step with PBS (150 µL/well) and fixation with EtOH (99%, 100 µL/well, 10 min) for the subsequent Crystal Violet assay [105, 106]. The experiments were performed using three independent biological replicates (*N* = 3), with technical duplicates for each experimental condition. The detailed protocols for the WST‐1 and Crystal Violet assays are described in the Supporting Information.

Cell viability was evaluated in a different workflow via WST‐1 assay after incubation (1, 3, 6, 24, 48, 72, and 96 h) with insulin‐loaded MSN (**MSN(Ins)**, **PEG‐MSN(Ins)**, or **PO_3_‐MSN(Ins)**), which were dispersed in an insulin‐free medium at equivalent insulin concentration (10 µg mL^−1^, 1.7 µM, 200 µL/well). Cell viability was also tested in starvation conditions (**Ins^(−)^**) after cell treatment with empty MSN (**MSN**, **PEG‐MSN**, or **PO_3_‐MSN**), which were dispersed in an insulin‐free medium at particle concentrations equivalent to the insulin‐loaded MSN (40 µg·mL^−1^, 200 µL/well). The positive (cells incubated with complete cell culture medium, **Ins^(+)^**, 10 µg mL^−1^, 1.7 µM) and negative control (non‐treated cells incubated in an insulin‐free medium, **Ins^(−)^**) were included in the analysis. Sample preparation and applications of the treatments were performed as described above. The experiments were performed with three independent cell preparations (biological triplicates; *N* = 3), and technical duplicates were included for each experimental condition.

### 4.8 Fluorescence Microscopy Experiments

For the live cell imaging experiments, the MSN and commercial insulin were previously labeled with Rhod‐ITC or FITC [32, 74]. Details are provided in the Supporting Information. HCEC‐1CT cells were seeded in Greiner Bio‐One CELLview four‐compartment tissue culture dishes (growth area: 1.9 cm^2^/compartment; 20 000 cells/compartment) and incubated at 37°C for 48 h prior to live‐cell imaging. Rhodamine‐labeled MSN (**MSN^Rhod^**, **PEG‐MSN^Rhod^**, and **PO_3_‐MSN^Rhod^**) containing **Ins^Flu^** loaded within the mesopores (**MSN^Rhod^(Ins^Flu^)**, **PEG‐MSN^Rhod^(Ins^Flu^)**, or **PO_3_‐MSN^Rhod^(Ins^Flu^)**) were dispersed in an insulin‐free cell culture medium, vortexed for 15 s, and sonicated for 5 min. Thereafter, the particle suspensions (500 µL) were added to the cells (equivalent to 10 µg·mL^−1^ of loaded **Ins^Flu^**). **Ins^Flu^** was dissolved in the insulin‐free cell culture medium (10 µg·mL^−1^, 1.7 µM), and 500 µL of the dispersion was added to the cells (positive control, **Ins^(+)^**) before incubation for 1, 3, 6, or 24 h at 37°C. Additional treatments with non‐confined **Ins^Rhod^** (control) or **Ins^Rhod^** loaded into FITC‐labeled particles (**MSN^Flu^(Ins^Rhod^)**, **PEG‐MSN^Flu^(Ins^Rhod^)**, or **PO_3_‐MSN^Flu^(Ins^Rhod^)**) were performed for 1, 3, 6, or 24 h at 37°C as described above. For testing energy‐dependent endocytosis, the cells were treated with **PO_3_‐MSN^Rhod^(Ins^Flu^)** (10 µg·mL^−1^ equivalent to loaded **Ins^Flu^**) and with non‐confined insulin (**Ins^Flu^**, 10 µg·mL^−1^, control) as described above, followed by incubation for 3 h or 6 h at 4°C and 37°C.

At the end of the treatments, cells were washed twice with 550 µL of Live Cell Imaging solution (LCI, Molecular Probes, Life Technologies, Thermo Fisher Scientific, USA) and incubated with CellMask Deep Red Plasma Membrane Stain (1:1000 dilution, 250 µL/well) for 15 min at 37°C [25, 107]. Cells were then washed twice with Dulbecco's Phosphate‐Buffered Saline (DPBS, 500 µL/well), and 400 µL of LCI was added to each compartment. Imaging was performed using a confocal LSM microscope Zeiss 710, equipped with ELYRA PS.1 and a Water Plan Apochromat 63x/1.2 objective. The ZEN 2012 Black Edition software (Zeiss Microscopy GmbH, Germany) was used for the analysis of the images, which were composed of the fluorescein (green), rhodamine (red), and Deep Red plasma membrane (cyan) channels, whose intensity distribution was evaluated from the corresponding 2.5D views. Fluorescein and rhodamine mean intensities were quantified from colocalization analysis by selecting representative regions of interest (ROI). Manders’ colocalization coefficient (values between 0 and 1) was additionally obtained from individual ROIs and used as an indicator of the fractional spatial overlap between fluorescein and rhodamine fluorescence signals. For all experiments, measurements were performed in technical duplicates on at least three independent cell preparations (biological triplicates, *N* = 3), and more than 25 ROI/cell (*n* ≥ 25) were quantified from a minimum of 12 randomly selected optical fields. Z‐stack imaging was additionally performed, yielding two 3D reconstructions per replicate (*n* = 6 images per condition), from which the internalization of insulin‐loaded MSN or non‐confined insulin (control, **Ins^(+)^**) was evaluated.

The description of the proteomic analysis used to further identify insulin in cell lysates is provided in the Supporting Information. Data are available via ProteomeXchange with identifier PXD074176.

### 4.9 Barrier Integrity and Permeability of Insulin and Lucifer Yellow Across Intestinal Cell Monolayers

TEER measurements were performed with the intestinal Caco‐2/HT29‐MTX‐E12 co‐culture model to assess the integrity of the cell monolayer, as described in previous protocols [108, 109]. A total cell density of 85 000 cells·cm^−2^ was seeded in Sarstedt TC inserts for 12 well‐plates (Ref. 833931041, PET membrane, pore size 0.4 µm, 1.1 cm^2^ growth area). Complete cell culture medium (1.5 mL) was placed on the basolateral compartment, while 500 µL of the cell suspension in complete cell culture medium was added to the apical side. This rendered 93 500 cells/well (84 150 Caco‐2 cells and 9 350 HT29‐MTX‐E12 cells). The Caco‐2/HT29‐MTX‐E12 co‐culture was incubated at 37°C for 14 days, with the cell culture medium replaced with fresh medium every 2 days. At the end of the differentiation time, a homogenous layer of mucus was visible on the cell co‐culture. The culture medium was removed, and the cells were treated with 500 µL of freshly prepared dispersions containing either insulin‐loaded phosphonated MSN (**PO_3_‐MSN(Ins)**, 100 µg·mL^−1^ equivalent to insulin, 17 µM), non‐confined insulin (**Ins^(+)^**, 100 µg·mL^−1^, 17 µM), or empty phosphonated MSN at concentrations equivalent to the **PO_3_‐MSN(Ins)** (**PO_3_‐MSN**, 340 µg·mL^−1^) in complete cell culture medium. The samples were added to the apical side of cell culture inserts (500 µL/well), and the medium in the basolateral compartment was replaced with fresh complete cell culture medium. TEER was measured before and immediately after treatment with **PO_3_‐MSN(Ins)**, **PO_3_‐MSN**, or non‐confined insulin **Ins^(+)^**, and following incubation at 37°C for 1, 3, 6, and 24 h using an Epithelial Voltohmmeter (EVOM2) coupled to a chopstick electrode pair (STX2, both from World Precision Instruments, Sarasota, FL, USA). A control consisting of non‐treated Caco‐2/HT29‐MTX‐E12 co‐culture was included in the experimental layout, and TEER was measured at the same points as for the treated cells. Prior to measurements, the electrodes were rinsed with ethanol and equilibrated in complete medium at room temperature for 20 min [109]. All experiments were performed using three independent cell preparations (biological triplicates; *N* = 3), including technical duplicates for each experimental condition. At least three readings were taken per well for each time point.

After incubation for 6 or 24 h, 100 µL aliquots were collected from the basolateral compartment of the cell culture inserts. To each aliquot, 100 µL of pre‐cooled MeOH/AcOH (90:10 v/v, −20°C) was added, and the mixtures were stored at −20°C for at least 1 h. Following FBS precipitation, the samples were centrifuged (12 000 rpm, 13 800 ×* g*, 20 min), and the supernatants were collected for HPLC–MS analysis. Insulin was additionally quantified in the apical and basolateral compartments of cell‐free culture inserts incubated at 37°C. This control experiment was performed under the same conditions set for the cell‐based studies, using **PO_3_‐MSN(Ins)** (100 µg·mL^−1^ equivalent to insulin, 17 µM) and non‐confined insulin (**Ins^(+)^**, 100 µg·mL^−1^, 17 µM), both dispersed in complete cell culture medium, added to the apical side (500 µL/well) and incubated for 6 or 24 h at 37°C. This setup enabled the assessment of maximum insulin permeability across the insert membrane, independent of the intestinal cell barrier. Details of the HPLC–MS method for insulin quantification are provided in the Supporting Information.

At the end of the 24 h incubation with **PO_3_‐MSN(Ins)**, **PO_3_‐MSN**, or non‐confined **Ins^(+)^**, the hydrophilic marker Lucifer Yellow was added to the apical compartment (500 µL/well) to evaluate monolayer tightness and paracellular permeability in treated cells compared with control (non‐treated Caco‐2/HT29‐MTX‐E12 co‐culture) [109]. The assay was also performed in permeable inserts without cells, as described for the cell‐free control experiment performed for insulin quantification. In this case, the fluorescence of Lucifer Yellow was measured in both apical and basolateral compartments after 1 h incubation at 37°C with the stock solution of Lucifer Yellow CH di‐lithium salt (0.1 mg·mL^−1^) in Hank's balanced salt solution (HBSS). Detailed protocol for the Lucifer Yellow assay is provided in the Supporting Information.

### 4.10 Evaluation of Insulin Bioactivity Following Transport Across the Intestinal Barrier

The bioactivity of insulin that crossed the barrier formed by the Caco‐2/HT29‐MTX‐E12 co‐culture in cell culture inserts was assessed using MCF‐7 breast cancer cells, an estrogen‐dependent model in which insulin can directly promote cell growth and proliferation by activating insulin receptor–mediated signaling pathways (PI3K/Akt and ERK1/2). These pathways regulate metabolic activity, cell cycle progression, and survival [85, 88, 110]. The viability of MCF‐7 cells was measured after incubation with media taken from the basolateral compartment of the cell culture inserts, in combination with standard (25 mM, Glu^25mM^) or high (50 mM, Glu^50mM^) glucose concentrations, to simulate normoglycemic and diabetic environments, respectively. To prepare the treatments, 400 µL aliquots were taken from the basolateral compartment of cell‐seeded inserts exposed to **PO_3_‐MSN(Ins)**, **PO_3_‐MSN**, and non‐confined **Ins^(+)^** after 24 h of incubation with Caco‐2/HT29‐MTX‐E12 cells. These aliquots were combined with 400 µL of complete cell culture medium for Caco‐2/HT29‐MTX‐E12 cells containing a standard glucose concentration for cell culture (Glu^25mM^). For the media containing Glu^50mM^, 400 µL of complete DMEM containing 100 mM glucose was added instead, yielding a final concentration of 50 mM glucose in the mixtures. Similarly, aliquots were taken from the basolateral compartment of the controls (non‐treated Caco‐2/HT29‐MTX‐E12 cells) and combined with cell culture media (Glu^25mM^ and Glu^50mM^), as described above. The samples prepared in the presence of Glu^25mM^ or Glu^50mM^ were stored at 4°C until application to MCF‐7 cells. With this purpose, 15 000 MCF‐7 cells (100 µL/well) were first seeded in 96‐well black‐sided plates (Sarstedt) and incubated at 37°C. After 48 h, confluency was observed, and the culture medium was removed. The MCF‐7 cells were washed with Dulbecco's phosphate‐buffered saline (DPBS, 100 µL/well), and 100 µL of the prepared treatments under both Glu^25mM^ and Glu^50mM^ conditions were added. Additional controls included MCF‐7 cells incubated under standard culturing conditions with the insulin supplement (**Ins^(+)^**, 0.1 µg·mL^−1^, 17 nM) or under insulin starvation (**Ins^(−)^**). Treated cells and controls (MCF‐7 medium + **Ins^(+)^** and MCF‐7 medium + **Ins^(−)^**) were incubated for 48 h at 37°C, and cell viability was subsequently measured using the CTB assay [104] to assess the effects of insulin treatments on cell proliferation under both Glu^25mM^ and Glu^50mM^ conditions. The experiments were performed using three independent cell preparations (biological triplicates; *N* = 3), with technical duplicates for each experimental condition. Details of the CTB assay protocol are provided in the Supporting Information.

### 4.11 Immunofluorescence Staining and Microscopy

Immunofluorescence experiments were performed to stain the TJ proteins Zonula occludens‐1 (ZO‐1) and Claudin‐4 (CLDN4), expressed in the Caco‐2/HT29‐MTX‐E12 intestinal co‐culture [109, 111]. Cells were seeded in 8‐well IbiTreat µ‐slides (REF: 80.826, ibidi GmbH, Gräfelfing, Germany) at a density of 85 000 cells·cm^−2^, as described for experiments using Sarstedt TC inserts. This corresponded to 85 000 cells/well (76 500 Caco‐2 cells and 8 500 HT29‐MTX‐E12 cells) in 200 µL of complete culture medium. After 14 days of differentiation, the medium was removed, and the cells were washed twice with DPBS before the addition of 200 µL of a freshly prepared suspension of fluorescein‐labeled insulin loaded onto phosphonated MSN (**PO_3_‐MSN(Ins^Flu^)**) at an insulin concentration of 100 µg·mL^−1^ (17 µM). Fluorescein‐labeled **PO_3_‐MSN** (**PO_3_‐MSN^Flu^**) were alternatively applied to the cells at the same particle concentration used in the **PO_3_‐MSN(Ins^Flu^)** treatment (260 µg·mL^−1^). The cells were incubated for 6 h at 37°C, and a control consisting of non‐treated Caco‐2/HT29‐MTX‐E12 cells was included in the experimental layout. The experiments were performed in three independent biological replicates (*N* = 3), including technical duplicates for each experimental condition. A detailed description of the implemented protocols for **PO_3_‐MSN** labeling and for ZO‐1 and CLDN4 staining is provided in the Supporting Information.

A Zeiss LSM710 laser scanning confocal microscope (ELYRA PS.1 system) equipped with a 63X/1.4 plan‐apochromatic oil immersion objective (Zeiss Microscopy GmbH, Germany) was used for imaging. For both treatments (**PO_3_‐MSN(Ins^Flu^)** and empty **PO_3_‐MSN**) and the control (non‐treated Caco‐2/HT29‐MTX‐E12 cells), at least three images were acquired per biological replicate (*N* = 3), each prepared in technical duplicate, yielding a total of *n* = 18 images per condition. Each image comprised ZO‐1, CLDN4, FITC (**Ins^Flu^** or **PO_3_‐MSN^Flu^**), and DAPI (nuclei staining) channels. In addition, Z‐stack imaging was performed to obtain 3D reconstructions, yielding *n* = 6 stacks per condition.

Orthogonal views (XY, XZ, and YZ) were generated using the DAPI channel as a reference for selecting the XY projections. Image analysis was performed using ImageJ. The XY thickness (µm) of ZO‐1 and CLDN4 staining was measured in 2D optical fields, using the cell nuclei (DAPI) signal as a positional reference. Each dataset was obtained from the analysis of at least 90 cells across three independent cell preparations (biological triplicates; *N* = 3). ZO‐1 and CLDN4 fluorescence signals were additionally quantified from maximum‐intensity projections of the respective channels in 3D reconstructions obtained from Z‐stack imaging (*n *= 6 total reconstructions derived from three independent biological triplicates, including technical duplicates).

### 4.12 Evaluation of Oral Delivery of Insulin and Insulin Tolerance in a Diabetic Mouse Model

The effect of insulin‐loaded MSN on insulin tolerance (circulating blood glucose concentration) was measured in fasted streptozotocin (STZ)‐induced diabetic C57BL/6J female mice (*n *= 5 mice per group). Mice were administered a single high dose of STZ (180 mg·kg^−1^, intraperitoneal injection), and those with two weekly blood glucose levels greater than 250 mg·dL^−1^ (13.9 mmol·L^−1^) were enrolled in the study. Prior to treatment, mice were fasted overnight (12 h) and counterbalanced into three groups: (A) mice treated with **sBL[PO_3_‐MSN(Ins)]** capsules (equivalent to 400 U·kg^−1^ of loaded insulin); (B) mice treated with a suspension of **PO_3_‐MSN(Ins)** (equivalent to 400 U·kg^−1^ of loaded insulin) in PBS containing dispersed sBL; (C) mice treated with capsules containing empty phosphonated particles (**sBL[PO_3_‐MSN]**, control capsules). In the suspension‐based formulations (group B), the protein excipient sBL content was equivalent to that used in capsules (groups A and C) and was adjusted to 3–4 mg according to mouse weight. Capsule formulations were prepared and administered using a Braintree Scientific Capsule Kit (X‐KIT M). Immediately after oral administration of the capsules (groups A and C) or particle suspensions (groups B), mice from group A were administered an additional oral gavage containing Alexa Fluor 647‐labeled MSN (**PO_3_‐MSN^AF^**) suspended in PBS (100 mg·kg^−1^) [24], which additionally enabled evaluation of particle biodistribution in intestinal tissues. Groups B (**PO_3_‐MSN(Ins)**‐suspension) and C (**sBL[PO_3_‐MSN]**, control capsules) received only additional PBS gavage. Blood glucose levels were then measured at 1, 2, 4, and 6 h post‐gavage. The protocol for the labeling of **PO_3_‐MSN** with Alexa Fluor 647 Succinimidyl Ester (**PO_3_‐MSN^AF^**) is provided in the Supporting Information.

### 4.13 Histopathology and Epifluorescence Microscopy of Intestinal Tissues

At 6 h post‐gavage, mice were euthanized by CO_2_ asphyxiation and exsanguinated by cardiocentesis for necropsy. Duplicate samples of ileum and colon of mice from group A (treated with **sBL[PO_3_‐MSN(Ins)]** capsules and additional **PO_3_‐MSN^AF^** gavage) and group B (treated with **PO_3_‐MSN(Ins)** and sBL suspended in PBS and subsequent PBS gavage) were collected for epifluorescence microscopy (frozen tissues stored at −80°C) and histopathological analysis (fixed in neutral‐buffered formalin). Samples for fluorescence microscopy were cryosectioned and counterstained with DAPI (10 µg·mL^−^^1^, 5 min). For each tissue sample, 10 sections of 5 µm thickness were prepared and analyzed using a Leica DMi8 Thunder Epifluorescence microscope (Leica Microsystems, Germany) with a 100x/1.40 plan‐apochromatic oil immersion objective and a K8 camera for the imaging of the Alexa Fluor 647 labeled samples. Tissue samples for histopathology were embedded in paraffin and stained with hematoxylin and eosin (H&E). The histopathological images were acquired with a 20x/0.75 plan‐apochromatic air objective and a Flexacam C1 camera. The Leica LAS X microscope software (Leica Thunder Imager, Leica Microsystems, Germany) was used for image acquisition, capturing brightfield or different fluorescence channels with the tile scan or Z‐stack function.

### 4.14 Statistical Evaluation

Statistical analyses and all graph generation were performed using OriginPro 2024 (OriginLab Corporation, Northampton, MA, USA). One‐way ANOVA and Fisher's tests were used to compare treatment groups, and *p*‐values < 0.05 were considered statistically significant. At least three independent cell preparations (biological triplicates; *N* = 3) were used for each experimental workflow.

## Author Contributions

C. I.‐M. designed and performed the synthesis and functionalization of the nanoparticles, insulin loading, and release experiments, as well as in vitro cell studies. E. J. optimized the protocols for synthesizing and labeling the materials. A. B. and C. G. performed proteomic analysis to detect insulin in cell lysates. T. K. optimized the protocol for evaluating insulin stability in simulated body fluids. M. G. and A. E. performed the in vivo insulin tolerance tests and prepared tissue samples for microscopy. A. H. and M. K. conducted histopathological and epifluorescence microscopy analyses on mouse tissues. H. K. performed NMR characterization and analysis. D. M. provided funding and technical support for the in vitro cell studies. M. M. and D. B. provided protocols and valuable assistance for the release studies and insulin tolerance testing, respectively. D. B. also provided guidance and supervision for storage, preparation, and microscopy analyses of mouse tissues. G. D. F. and F. K. contributed to the experimental design, provided funding, and supervised the project. C. I. M. drafted the original manuscript, and all authors contributed to the discussion and interpretation of the results.

## Ethics Statement

All procedures involving in vivo studies were conducted in accordance with institutional and national guidelines for the care and use of laboratory animals and were approved by the Institutional Animal Care and Use Committee (IACUC) at the University of California, Davis (approval number 23211).

## Associated Content

List of abbreviations; materials and methods; hydrodynamic diameters and pH‐dependent colloidal stability and zeta potential of MSN; characterization of the porosity of MSN; TGA and DSC analyses of the materials obtained; solid‐state ^29^Si CP/MAS NMR spectra of MSN; ATR‐FTIR spectra of MSN; CD spectra of sBL, native BL, and insulin released from MSN(Ins)‐based formulations; insulin release from MSN‐based formulations without sBL and long‐term stability of MSN carriers in buffer at pH 1.2 and 7.4; kinetics and mechanistic modeling of insulin release from tablet formulations under intestinal conditions; insulin stability in the presence of digestive enzymes and dissolution test of sBL tablets; cytotoxicity of “empty” MSN and insulin‐loaded MSN; UV/Vis characterization of labeled materials; structural MS characterization of the fluorescein‐labeled insulin (**Ins^Flu^**); live fluorescence imaging of HCEC‐1CT cells incubated with insulin‐loaded MSN (**MSN^Rhod^(Ins^Flu^)**); 2.5D views obtained from live cell fluorescence imaging; colocalization graphs obtained from live cell fluorescence imaging; quantification of particle internalization and intracellular release of insulin from live cell imaging; live fluorescence imaging of HCEC‐1CT cells incubated with insulin‐loaded MSN (**MSN^Flu^(Ins^Rhod^)**); energy‐dependent endocytosis of insulin‐loaded MSN; detection of insulin in cell lysates via untargeted proteomics (data are available via ProteomeXchange with identifier PXD074176); paracellular permeability of Lucifer Yellow and insulin through Caco‐2/HT29‐MTX‐E12 cells or cell‐free inserts; immunofluorescence staining of tight junction proteins and interaction of **PO_3_‐MSN** carriers with intestinal cells; histopathological characterization of intestinal tissue from mice gavaged with MSN(Ins)‐based formulations.

## Conflicts of Interest

The authors declare no conflict of interest.

## Supporting information




**Supporting File**: smll73007‐sup‐0001‐SuppMat.pdf.

## Data Availability

The data that support the findings of this study are available from the corresponding author upon reasonable request.
