# Supplementary material for: Mesoporous Silica Nanoparticles‐Based Formulations for Enhanced Oral Delivery of Peptide Drugs: A Case Study on Insulin
Source: Small. 2026 Mar 22;22(24):e13347. doi: 10.1002/smll.202513347 (PMC13114519; doi:10.1002/smll.202513347)
Supplement: Supplementary file 1 — Supporting File: smll73007‐sup‐0001‐SuppMat.pdf. [file SMLL-22-e13347-s001.pdf]

Supporting Information

Mesoporous Silica Nanoparticles–Based Formulations for Enhanced Oral Delivery of Peptide Drugs: A Case Study on Insulin

Claudia Iriarte-Mesa,[1,2] Estelle Juère,[1] Andrea Bileck,[3,4] Thomas Kremsmayr,[5] Michael L. Goodson,[6,7] Allison Ehrlich,[6] Adnan Hodžić,[8] Martin Kunert,[8] Christopher Gerner,[3,4] Hanspeter Kählig,[9] Doris Marko,[10] Markus Muttenthaler,[5,11] David Berry,[8,12] Giorgia Del Favero,[10,13] Freddy Kleitz[1*]*

[1] Department of Functional Materials and Catalysis, Faculty of Chemistry, University of Vienna, 1090 Vienna, Austria

[2] Vienna Doctoral School in Chemistry (DoSChem), University of Vienna, 1090 Vienna, Austria

[3] Department of Analytical Chemistry, Faculty of Chemistry, University of Vienna, 1090 Vienna, Austria

[4] Joint Metabolome Facility, University of Vienna and Medical University of Vienna, 1090 Vienna, Austria

[5] Institute of Biological Chemistry, Faculty of Chemistry, University of Vienna, 1090 Vienna, Austria

[6] Department of Environmental Toxicology, College of Agriculture and Environmental Science, University of California, 95616 Davis, CA, USA

[7] Department of Anatomy, Physiology and Cell Biology, School of Veterinary Medicine, University of California, 95616 Davis, CA, USA

[8] Centre for Microbiology and Environmental Systems Science, Department of Microbiology and Ecosystem Science, Division of Microbial Ecology, University of Vienna, 1030 Vienna, Austria

[9] Department of Organic Chemistry, Faculty of Chemistry, University of Vienna, 1090 Vienna, Austria

[10] Department of Food Chemistry and Toxicology, Faculty of Chemistry, University of Vienna, 1090 Vienna, Austria

[11] Institute for Molecular Bioscience, The University of Queensland, 4072 Brisbane, Queensland, Australia

[12] Joint Microbiome Facility of the Medical University of Vienna and the University of Vienna, 1090 Vienna, Austria

[13] Core Facility Multimodal Imaging, Faculty of Chemistry, University of Vienna, 1090 Vienna, Austria

Corresponding authors (*): giorgia.del.favero@univie.ac.at; freddy.kleitz@univie.ac.at

TABLE OF CONTENTS

LIST OF ABBREVIATIONS 3
MATERIALS 5
METHODS 6
Synthesis of dendritic mesoporous silica nanoparticles. 6
Preparation of the buffers for release tests. 6
Stability of non-confined insulin in the presence of digestive enzymes. 6
Dissolution testing of sBL tablets. 6
Viability assays for HCEC-1CT cells. 7
Labeling of mesoporous silica nanoparticles for confocal microscopy. 7
Labeling of insulin. 8
Proteomic analysis for the detection of insulin in cell lysates 9
Lucifer Yellow assay. 9
CellTiter-Blue (CTB) assay for MCF-7 breast cancer cells. 10
Protocol for tight junction protein immunostaining. 10
MSN labeling for assessing particle biodistribution in intestinal mouse tissues. 10
Material characterization. 11

LIST OF FIGURES

S1. Hydrodynamic diameters and pH-dependent colloidal stability and zeta potential of MSN. 14
S2. Characterization of the porosity of MSN. 15
S3. Thermogravimetric (TGA) and differential scanning calorimetry (DSC) analyses of the materials. 16
S4. Solid-state ^{29}Si CP/MAS NMR spectra of MSN. 18
S5. ATR-FTIR spectra of MSN. 19
S6. Circular dichroism spectra of sBL, native BL, and insulin released from MSN(Ins)-based formulations. 20
S7. Insulin release from MSN-based formulations without sBL and long-term stability of MSN carriers in buffers at pH 1.2 and 7.4. 21
S8. Kinetics and mechanistic modeling of insulin release from tablet formulations under intestinal conditions. 22
S9. Insulin stability in the presence of digestive enzymes and dissolution test of sBL tablets. 23
S10. Cytotoxicity of “empty” MSN and insulin-loaded MSN. 24
S11. UV/Vis characterization of labeled materials. 25
S12. Structural MS characterization of the fluorescein-labeled insulin (**Ins^{Flu}**). 25
S13. Live fluorescence imaging of HCEC-1CT cells incubated with insulin-loaded MSN (**MSN^{Rhod}(Ins^{Flu})**). 26
S14. 2.5D views obtained from live cell fluorescence imaging. 27
S15. Colocalization graphs obtained from live cell fluorescence imaging. 28
S16. Quantification of particle internalization and intracellular release of insulin from live cell imaging. 29
S17. Live fluorescence imaging of HCEC-1CT cells incubated with insulin-loaded MSN (**MSN^{Flu}(Ins^{Rhod})**). 30
S18. Energy-dependent endocytosis of insulin-loaded MSN. 31
S19. Detection of insulin in cell lysates *via* untargeted proteomics 32
S20. Paracellular permeability of Lucifer Yellow and insulin through Caco-2/HT29-MTX-E12 cells or cell-free inserts. 32
S21. Immunofluorescence staining of tight junction proteins and interaction of **PO_3-MSN** carriers with intestinal cells. 33
S22. Histopathological characterization of intestinal tissue from mice gavaged with MSN(Ins)-based formulations. 34

LIST OF TABLES

S1. Physico-chemical parameters of the functionalized, insulin-loaded, and/or labeled MSN. 17
S2. Assignment of the signals of the solid-state ^{29}Si CP/MAS NMR spectra. 18
S3. Signal assignment of the ATR-FTIR spectra of functionalized and rhodamine-labeled MSN. 20
S4. Kinetic parameters for intestinal-phase insulin release from sBL-based tablets after transference from pH 1.2 to pH 7.4. 23

LIST OF SCHEMES

S1. Synthetic steps for the labeling of silica nanoparticles with rhodamine B isothiocyanate. 7
S2. Synthetic steps for the labeling of silica nanoparticles with FITC. 8
S3. Schematic representation of insulin labeling with fluorescein (**Ins^{Flu}**). 8
S4. Synthetic steps for the labeling of silica nanoparticles with Alexa Fluor™ 647 Succinimidyl Ester. 11

REFERENCES 35

LIST OF ABBREVIATIONS

**ACN**	Acetonitrile
**AcOH**	Acetic acid
**AF**	Alexa Fluor™ 647
**APTES**	(3-Aminopropyl)triethoxysilane
**ATR-FTIR**	Attenuated total reflectance Fourier-transform infrared spectroscopy
**ATP**	Adenosine triphosphate
**AUC**	Area under the curve
**BET**	Brunauer-Emmet-Teller equation
**BL**	β-Lactoglobulin (native protein)
**$CaCl_2$**	Calcium chloride
**CD**	Circular dichroism
**CLDN4**	Claudin-4
**CP**	Cross-polarization
**CP/MAS NMR**	Cross-polarization magic angle spinning nuclear magnetic resonance
**CTAC**	Cetyltrimethylammonium chloride
**CTB**	CellTiter-Blue
**DAPI**	4′,6-Diamidino-2-phenylindole
**DLS**	Dynamic light scattering
**DMEM**	Dulbecco's Modified Eagle Medium
**DMSO**	Dimethyl sulfoxide
**DPBS**	Dulbecco's phosphate-buffered saline
**DSC**	Differential scanning calorimetry
**DSS**	2,2-Dimethyl-2-silapentane-5-sulfonate sodium salt
**DTT**	Dithiothreitol
**ERK1/2**	Extracellular signal-regulated kinase 1/2
**ESI**	Electrospray ionization
**EtOH**	Ethanol
**FA**	Formic acid
**FBS**	Fetal bovine serum
**FITC**	Fluorescein isothiocyanate
**GLP-1**	Glucagon-like peptide-1
**Glu^{25mM}**	25 mM glucose (standard concentration in cell culture medium)
**Glu^{50mM}**	50 mM glucose in cell culture medium
**HBSS**	Hank’s balanced salt solution buffer
**HCEC-1CT**	Immortalized human colonic epithelial cells
**HCl**	Hydrochloric acid
**HEPES**	4-(2-Hydroxyethyl)-1-piperazineethanesulfonic acid
**HPLC–MS**	High-performance liquid chromatography-mass spectrometry
**HR-ESI-MS**	High-resolution electrospray ionization mass spectrometry
**H&E**	Hematoxylin and eosin fast staining kit
**IAA**	2-Iodoacetamide
**$Ins^{(+)}$**	Cell culture medium containing insulin
**$Ins^{(-)}$**	Cell culture medium without insulin
**Ins^{Flu}**	Insulin labeled with fluorescein
**Ins^{Rhod}**	Insulin labeled with rhodamine B
**InsR**	Insulin receptor
**IRS-1**	Insulin receptor substrate-1
**K_0**	Zero-order release constant
**K_1**	First-order release constant
**KCl**	Potassium chloride
**K_H**	Higuchi diffusion constant
**KH_2PO_4**	Potassium dihydrogen phosphate
**K_{KP}**	Korsmeyer–Peppas kinetic constant
**LCI**	Live cell imaging solution
**LFQ**	Label-free quantification values
**MAS**	Magic angle spinning
**MeOH**	Methanol
**$MgCl_2$**	Magnesium chloride
**M_r**	Insulin released (%), normalized to the amount of insulin remaining after transfer of the tablets from pH 1.2 to 7.4
**MRE**	Mean residue ellipticity
**MRW**	Mean residue weight
**MSN**	Mesoporous silica nanoparticles
**MSN(Ins)**	Mesoporous silica nanoparticles containing insulin within the mesopores
**MSN^{Flu}**	Mesoporous silica nanoparticles labeled with fluorescein
**$MSN^{Flu}(Ins^{Rhod})$**	Mesoporous silica nanoparticles labeled with fluorescein and loaded with rhodamine B
**MSN^{Rhod}**	Mesoporous silica nanoparticles labeled with rhodamine B
**$MSN^{Rhod}(Ins^{Flu})$**	Mesoporous silica nanoparticles labeled with rhodamine B and loaded with fluorescein

**NaOH**	Sodium hydroxide
**$NH_4H_2PO_4$**	Ammonium dihydrogen phosphate
**n_{KP}**	Release exponent according to the Korsmeyer–Peppas kinetic model
**NLDFT**	Non-local density functional theory
**PBS**	Phosphate-buffered saline
**PDI**	Polydispersity indexes
**PEG-MSN**	Mesoporous silica nanoparticles functionalized with polyethylene glycol functions
**PEG-MSN(Ins)**	PEGylated nanoparticles containing insulin loaded within the mesopores
**PEG-MSN^{Flu}**	PEGylated nanoparticles labeled with fluorescein
**PEG-MSN^{Rhod}**	PEGylated nanoparticles labeled with rhodamine B
**PEG-silane**	Ethoxy silane functionalized polyethylene glycol
**PET**	Polyethylene terephthalate
**PI3K**	Phosphoinositide 3-kinase
**PKB/Akt**	Protein kinase B
**PMSF**	Phenylmethanesulfonyl fluoride
**PO_3-MSN**	Mesoporous silica nanoparticles functionalized with phosphonated moieties
**PO_3-MSN^{AF}**	Phosphonated nanoparticles labeled with Alexa Fluor 647
**PO_3-MSN^{Flu}**	Phosphonated nanoparticles labeled with fluorescein
**PO_3-MSN^{Rhod}**	Phosphonated nanoparticles labeled with rhodamine B
**PO_3-MSN(Ins)**	Phosphonated nanoparticles with loaded insulin
**PO_3-MSN(Ins^{Flu})**	Phosphonated nanoparticles with loaded fluorescein-labeled insulin
**PSD**	Pore size distributions
**Rhod-ITC**	Rhodamine B isothiocyanate
**ROI**	Regions of interest
**RP-HPLC**	Reversed-phase high-performance column chromatography
**S_{BET}**	Specific surface area determined using the Brunauer-Emmet-Teller equation
**sBL**	Succinylated β-lactoglobulin
**sBL[Ins]**	Tablets with succinylated β-lactoglobulin and insulin
**sBL[MSN(Ins)]**	Tablets with succinylated β-lactoglobulin and insulin-loaded nanoparticles
**SGF**	Simulated gastric fluid
**SIF**	Simulated intestinal fluid
**SNAC**	N-(8-[2-hydroxybenzoyl]amino)caprylate
**STAT3**	Signal transducer and activator of transcription 3
**STZ**	Streptozotocin
**TEA**	Triethanolamine
**TEER**	Transepithelial electrical resistance
**TEM**	Transmission electron microscopy
**TEOS**	Tetraethyl orthosilicate
**TFA**	Trifluoroacetic acid
**TGA**	Thermogravimetric analysis
**THMP**	3-(Trihydroxysilyl)propyl methylphosphonate
**TJs**	Tight junction
**UHPLC–MS**	Ultrahigh-performance liquid chromatography coupled to mass spectrometry
**WA-XRD**	Wide-angle X-ray diffraction
**WST-1**	Water-soluble tetrazolium salt (4-[3-(4-Iodophenyl)-2-(4-nitro-phenyl)-2H-5-tetrazolio]-1,3-benzene sulfonate)
**ZO-1**	Zonula occludens-1

MATERIALS

Tetraethyl orthosilicate (TEOS, 98%), hexane (≥99%), cetyltrimethylammonium chloride (CTAC, 25 wt% in H_2O), fluorescein isothiocyanate (FITC, ≥90%), rhodamine B isothiocyanate (Rhod-ITC, >70%), 3-(trihydroxysilyl)propyl methylphosphonate (THMP, monosodium salt solution, 50 wt% in H_2O), anhydrous toluene (99.8%), methanol (MeOH, ≥99.9%), Pancreatin from porcine pancreas (suitable for cell culture, 4 × USP specifications), donkey serum (D9663), D-(+)-glucose (suitable for cell culture), streptozotocin (STZ, ≥75% α-anomer basis, ≥98%), and insulin (human recombinant, 5800 Da) were purchased from Sigma-Aldrich, Merck (Taufkirchen, Germany). PEG-silane ($(CH_3O)_3Si$-PEG-OCH_3, 2 kDa) was obtained from Rapp Polymere (Tübingen, Germany). Pepsin was provided by Acros Organics, VWR International (Vienna, Austria). Triethanolamine (TEA, 98%), sodium hydroxide (NaOH, 1.0 N standardized solution), anhydrous ethanol (EtOH, 95%), (3-aminopropyl)triethoxysilane (APTES, 98%), and dimethyl sulfoxide (DMSO, ≥99%) were purchased from Alfa Aesar (Massachusetts, USA). Hydrochloric acid (HCl, 37%) was acquired from Fluorochem (Hadfield, UK). HEPES (4-(2-hydroxyethyl)-1-piperazineethanesulfonic acid) buffer, glycine, Triton X-100, and hematoxylin and eosin (H&E) fast staining kit were obtained from Carl Roth (Karlsruhe, Germany). Succinylated β-lactoglobulin (sBL, 50% succinylated) was provided by Aventus Innovations (Levis, QC, Canada). The cell proliferation reagent WST-1 (water-soluble tetrazolium salt 4-[3-(4-iodophenyl)-2-(4-nitro-phenyl)-2H-5-tetrazolio]-1,3-benzene sulfonate) was purchased from Roche Diagnostics (Mannheim, Germany). Crystal Violet cell stain solution was provided by Cell Biolabs (San Diego, CA, USA). Lucifer Yellow CH di-lithium salt was purchased from Santa Cruz Technologies (Dallas, TX, USA). *CellTiter-Blue*® (CTB) reagent was purchased from Promega (Madison, WI, USA). HEPES buffer, $CaCl_2$, $MgCl_2$, glycine, and Triton X-100 were purchased from Carl Roth (Karlsruhe, Germany). Methanol (MeOH) and glacial acetic acid (AcOH) were acquired from Honeywell (Seelze, Germany) and Merck (Darmstadt, Germany), respectively. Primary antibodies for immunofluorescence experiments were obtained from Abcam (Cambridge, U.K.): anti-Zonula occludens-1 (ZO-1, ab190085, goat polyclonal) and anti-Claudin-4 (CLDN4, ab53156, rabbit polyclonal). The mounting medium containing DAPI (4′,6-diamidino-2-phenylindole, ab104139) was also acquired from Abcam (Cambridge, U.K.). The Alexa Fluor™ 647 NHS ester (succinimidyl ester) used for MSN labeling, as well as the CellMask™ Deep Red Plasma Membrane Stain (C10046), and fluorescently labeled secondary antibodies: Alexa Fluor™ 647 donkey anti-goat IgG (H+L, 705-605-003) and Alexa Fluor™ 568 donkey anti-rabbit IgG (H+L, A10042), were obtained from Probes-Life Technologies Invitrogen, Thermo Fisher Scientific (Waltham, MA, USA). If not otherwise specified, materials for cell culture were purchased from GIBCO Invitrogen (Karlsruhe, Germany), Sigma-Aldrich, Merck (Munich, Germany), Sarstedt AG & Co (Nümbrecht, Germany), Lonza Group Ltd (Basel, Switzerland), VWR International GmbH (Vienna, Austria), and Thermo Fisher Scientific (Vienna, Austria).

METHODS

Synthesis of dendritic mesoporous silica nanoparticles. The synthesis of dendritic mesoporous silica nanoparticles (MSN) was performed according to a previously reported procedure.[1] An aqueous solution was prepared by mixing TEA (360 mg) and CTAC (8 mL) with 72 mL of deionized water, followed by 1 h stirring (150 rpm) at 60°C. An organic mixture of hexane (32 mL) and TEOS (8 mL) was then added dropwise. The biphasic system was slowly stirred (150 rpm) at 60°C overnight. Prior to centrifugation (10,000 rpm;15,600 x *g*; 20 min), the organic phase was removed, and the particles were subsequently dried at 100°C overnight. To remove the organic templates, the product (**MSN**) was extracted for 2 h with 100 mL of EtOH and one drop of HCl (37%, 12 N) at 90°C and washed thrice with EtOH. The **MSN** were dried overnight at 100°C and further calcined at 550°C for 5 h.

Preparation of the buffers for release tests. For the preparation of the pH 1.2 buffer, 4 g NaCl and 14 mL HCl (37%, 12 N) were mixed in 2 L of nanopure H_2O (ultrapure type I, 18.2 MΩ·cm, purified using a PURELAB® flex 3 system, ELGA LabWater, UK). The pH 7.4 buffer was obtained by dissolving 13.6 g of KH_2PO_4 in 1.3 L of nanopure H_2O and adding 380 mL of 0.2 M NaOH. The pH was adjusted to 7.4 using 0.2 M NaOH, and the volume was completed to 2 L with nanopure H_2O.

Buffers containing digestive enzymes were prepared according to a published protocol to assess peptide stabilities under gastric and intestinal conditions.[2] Simulated gastric fluid (SGF) was obtained after adding 320 mg of pepsin (2167 U/mg of protein) to 100 mL of pH 1.2 buffer. For the preparation of simulated intestinal fluid (SIF), 6.8 g of KH_2PO_4 were dissolved in 250 mL of nanopure H_2O and combined with 100 mL of 0.2 M NaOH and 900 mL of nanopure H_2O. The pH was adjusted to 6.8 using 0.2 M NaOH, and the final volume was then brought to 1 L with nanopure H_2O. Finally, 250 mg of pancreatin (4 x USP activity: 1,400 FIP-U/g Protease, 24,000 FIP-U/g Lipase, 30,000 FIP-U/g Amylase) was added to 100 mL of the freshly prepared pH 6.8 buffer. The digestive enzymes were added shortly before starting the stability experiments, and further sonication was required to homogenize the enzyme dispersions.

Stability of non-confined insulin in the presence of digestive enzymes. To evaluate the stability of non-confined insulin in simulated body fluids (SGF and SIF), 80 µL of a stock solution of the commercial drug in water (200 $\mu g\cdot mL^{-1}$, 34 µM) was added to 1.92 mL of SGF or SIF, previously filtered (Nalgene™ Syringe Filters, 0.2 µm pore size, 13 mm diameter) and pre-incubated for 15 min at 37ºC. The SGF or SIF solutions containing insulin (8 $\mu g\cdot mL^{-1}$, 1.4 µM) were continuously shaken at 37ºC for 24 h. Aliquots of 100 µL were taken out at predetermined time intervals (0, 0.04, 0.08, 0.25, 0.5, 1, 2, 4, 6, and 24 h) and replaced by 100 µL of the respective fresh SGF or SIF to maintain the volume constant. The insulin concentration was further quantified in each aliquot using an ultra-high-performance liquid chromatography system coupled to an LTQ Orbitrap Velos mass spectrometer (UHPLC-MS) after the addition of 100 µL of MeOH or trifluoroacetic acid (TFA, 5% v/v in H_2O) for experiments performed in SGF or SIF, respectively. Three independent experiments were carried out. The insulin concentrations at the studied time slots were expressed as relative values (%) with respect to the initial total concentration (t_0), determined from UHPLC-MS analysis.

Dissolution testing of sBL tablets. The same procedure implemented for release tests was performed using sBL-tablets (without non-confined insulin or insulin-loaded MSN) to evaluate the stability and solubility of the succinylated protein excipient (sBL, 114 mg) in control conditions (pH 1.2 or pH 7.4 buffers) or in buffers containing digestive enzymes (SGF or SIF). The sBL-tablets were immersed in 100 mL of the buffers (pH 1.2 or 7.4), SGF or SIF. After

soaking for 1, 2, 2.5, 3, 4, 5, or 7 h, the remaining solids were removed from the solutions and placed for 3 min in 5 mL of PBS (for experiments performed in control conditions) or quenching solutions of MeOH and TFA (5% v/v in H_2O) for experiments carried out in SGF and SIF, respectively. After lyophilization for 20 h, the sBL content (wt%) was calculated by comparing the weight of the dry solids with that of control tablets containing the exact amount of sBL in the tested tablets at the beginning of the experiment. Such control tablets were lyophilized but not immersed in buffers, SGF, or SIF.

Viability assays for HCEC-1CT cells. The viability of HCEC-1CT cells was determined with the cell proliferation reagent WST-1 (4-[3-(4-iodophenyl)-2-(4-nitro-phenyl)-2H-5-tetrazolio]-1,3-benzene sulfonate). This tetrazolium salt is cleaved by cellular enzymes (mitochondrial dehydrogenases) once added to viable cells. Therefore, the spectrophotometric quantification of formazan produced by metabolically active cells correlated with cell viability.[3,4] After incubation with particles, the cells were washed with phenol red-free Dulbecco's Modified Eagle Medium (DMEM, 200 µL/well) to remove the cell culture medium containing treatments. WST-1 solution (WST-1:phenol red-free DMEM, 1:20) was then applied (100 µL/well), and the cells were incubated for 30 min at 37°C. The formazan absorbance was subsequently measured at 450 nm against a background blank (WST-1 solution incubated without cells, 100 µL/well) using a plate reader (CYTATON 5762, BioTek Instruments, Winooski, VT, USA). The reference wavelength was set to 650 nm to reduce baseline drift and refractive index changes induced by baseline wander. Cell viability was additionally evaluated *via* cell biomass determination (Crystal Violet assay).[5,6] After fixation with cold EtOH (99%, 100 µL/well, 10 min), cell staining was performed for 5 min using crystal violet solution (0.1%, 50 µL/well). Cells were washed four times using autoclaved water (150 µL/well) and were then lysed for 10 min on an orbital shaker (500 $U \cdot min^{-1}$) using a destaining solution (99% EtOH, 1% AcOH, 50 µL/well) before measuring crystal violet absorbance at 595 nm (CYTATON 5762 plate reader, BioTek Instruments, Winooski, VT, USA). Cell viabilities (both WST-1 metabolism and cell biomass, %) associated with each treatment were compared with the respective positive controls (treatment/control (**Ins**$^{(+)}$) [%]). The experiments were performed in three independent biological replicates ($N = 3$), and technical duplicates were measured for each experimental condition. Data groups were compared using one-way ANOVA and Fisher tests, with a significance threshold of $p < 0.05$.

Labeling of mesoporous silica nanoparticles for confocal microscopy. The calcined and functionalized MSN were labeled with rhodamine B isothiocyanate (Rhod-ITC) based on a published method (Scheme 1), involving the covalent coupling of Rhod-ITC with the amino-functionalized silane (3-aminopropyl)triethoxysilane (APTES).[1] First, Rhod-ITC (24 mg, 4 eq.) was dissolved in 10 mL of anhydrous DMSO, and APTES (2.6 µL, 1 eq.) was added at room temperature (25ºC). The reaction was conducted in the dark under stirring (800 rpm) and in an argon atmosphere for 24 h, while 100 mg of the calcined or functionalized particles (**MSN**, **PEG-MSN**, **PO_3-MSN**) was degassed overnight at 80°C. The particles were then dispersed in 30 mL of anhydrous toluene at 50ºC under stirring (700 rpm).

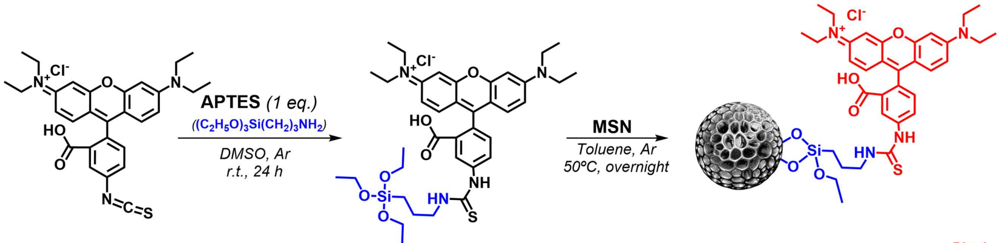

Rhod-ITC *(4 eq.)* | Rhod-APTES silane | rhodamine-labeled MSN (MSN^{Rhod})

Scheme S1. Synthetic steps for the labeling of silica nanoparticles with rhodamine B isothiocyanate.

Subsequently, the Rhod-APTES stock solution was added to each silica dispersion (50 µL to **MSN** dispersion or 100 µL to both **PEG-MSN** or **PO$_3$-MSN** dispersions). The grafting reactions were kept overnight at 50ºC under stirring (700 rpm) and argon atmosphere. The rhodamine-labeled MSN (**MSN^{Rhod}**, **PEG-MSN^{Rhod}**, **PO$_3$-MSN^{Rhod}**) were recovered by centrifugation (9,000 rpm; 11,770 x *g*; 20 min), washed once with toluene and thrice with EtOH, and then dried at 35°C for 24 h.

The same protocol was implemented for the MSN labeling with FITC (Scheme 2).[7,8] In this case, a fluorescein-APTES conjugate was prepared by dissolving FITC (26 mg, 4 eq.) in 10 mL of anhydrous EtOH and adding APTES (3 µL, 1 eq.) to the mixture. The reaction was kept at room temperature under stirring (800 rpm) and argon atmosphere for 24 h before adding the FITC-APTES stock solution to the MSN dispersions (**MSN**, **PEG-MSN**, or **PO$_3$-MSN**) as described above for rhodamine B labeling. The fluorescein-labeled MSN (**MSN^{Flu}**, **PEG-MSN^{Flu}**, **PO$_3$-MSN^{Flu}**) were recovered, washed, and stored in the same conditions implemented for the rhodamine-labeled particles.

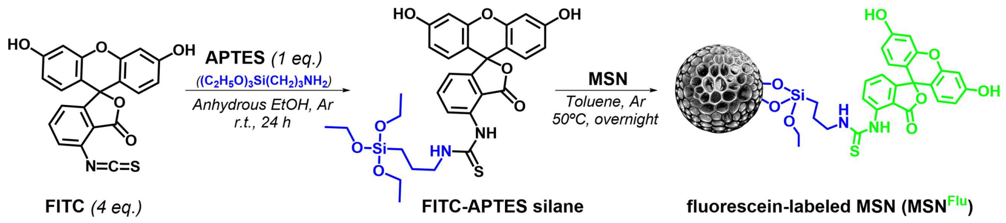

Scheme S2. Synthetic steps for the labeling of silica nanoparticles with FITC.

Labeling of insulin. FITC was coupled to insulin according to a published protocol.[1,9] FITC (5 mg, 7 eq.) was dissolved in 500 µL of DMSO. The fluorophore solution (100 µL) was added to insulin (10 mg, 1 eq.) previously dissolved in 1 mL of 0.1 M Na_2CO_3 (pH 9.5). The mixture was stirred at room temperature for 1 h and protected from light exposure (Scheme S1).

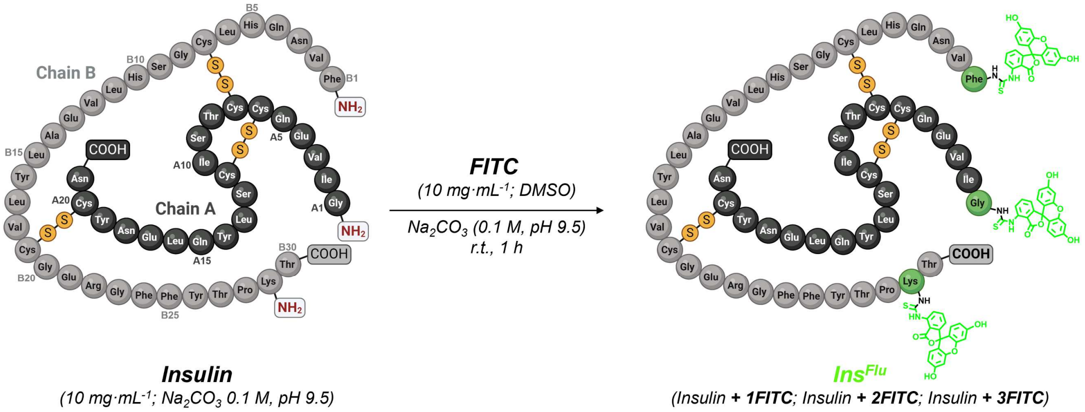

Scheme S3. Schematic representation of insulin labeling with fluorescein (**Ins^{Flu}**). FITC binds to the three primary amine sites B1 (Phe), A1 (Gly), and/or B29 (Lys) of insulin and forms mono-, di-, and/or tri-conjugates (created with BioRender.com).

After adding 2 mL of 0.1 M Na_2CO_3 (pH 9.5), the product was purified by reversed-phase high-performance column chromatography (RP-HPLC). The labeled sample was injected on a Waters Prep 150 System on a Kromasil C_4 semipreparative RP-HPLC column using a gradient from 5% to 45% solvent B (ACN with 0.08% TFA) in solvent A (H_2O with 0.1% TFA) over 30 min at a flow rate of 10 mL·min^{-1}. The fractions of all UV peaks with absorbance above 100 mAU were collected and analyzed by direct injection mass spectrometry (20 µL) on a Thermo Fisher Scientific MSQ system. The fractions containing the conjugate (**Ins^{Flu}**) were combined and lyophilized overnight. The lyophilized

Ins^{Flu} powder was characterized by high-resolution electrospray ionization mass spectrometry (HR-ESI-MS) and stored at -20°C for further use.

Proteomic analysis for the detection of insulin in cell lysates. To perform proteomic analysis, 600,000 cells were seeded in T75 flasks using 10 mL of complete cell culture medium. The cells were incubated for 48 h at 37ºC, and once confluent, treatments with insulin-loaded MSN (**MSN(Ins)**, **PEG-MSN(Ins)**, or **PO_3-MSN(Ins)**) were applied at an equivalent insulin concentration of 10 μg mL^{-1} (1.7 μM), as in non-confined insulin as a positive control (**Ins$^{(+)}$**). All treatments were prepared in an insulin-free cell culture medium. A negative control (starved cells without insulin) was additionally tested *via* incubation with an insulin-free cell culture medium (**Ins$^{(-)}$**). After 6 h incubation at 37ºC, the cells were lysed under mechanical shear stress using isotonic lysis buffer (10 mM HEPES/NaOH, pH 7.4, 0.25 M sucrose, 10 mM NaCl, 3 mM $MgCl_2$, 0.5% Triton X-100), supplemented with Protease and Phosphatase Inhibitor Cocktail (Sigma-Aldrich, Vienna, Austria) and 1 mM of the protease inhibitor phenylmethanesulfonyl fluoride (PMSF, Thermo Fisher Scientific, USA). For protein digestion, the ProtiFi S-trap™ protocol was employed.[10] In short, proteins were reduced and alkylated using dithiothreitol (DTT) and 2-iodoacetamide (IAA), respectively, followed by the addition of trapping buffer (90% v/v methanol, 0.1 M triethylammonium bicarbonate, pH 8.5). Samples were loaded onto S-trap™ cartridges and digested with Trypsin/Lys-C (Promega Corporation, Madison, WI, USA) for 2 h at 37°C. Supernatants containing the digested peptides were eluted, dried, and reconstituted in 5 μL formic acid (FA) containing 10 fmol of four synthetic standard peptides and diluted with 40 μL of mobile phase A (98% H_2O, 2% ACN, 0.1% FA). LC-MS/MS measurements were performed on a timsTOF Pro mass spectrometer (Bruker Daltonics) hyphenated with a Dionex UltiMate™ 3000 RSLCnano system (Thermo Fisher Scientific, Austria). The peptide solution (5 μL) was loaded onto an Acclaim™ PepMap™ C_{18} 100 pre-column (Thermo Fisher Scientific, Austria) at a flow rate of 10 μL·min^{-1} using mobile phase A and eluted onto an Aurora Series emitter column (Ionopticks) applying a flow rate of 300 nL·min^{-1}. Separation was achieved by applying a gradient of 8% to 40% of mobile phase B (80% ACN, 2% H_2O, 0.1% FA) over 90 min. For MS detection, scans were performed from m/z 400–1400 at a resolution of 70,000 (at $m/z = 200$). Fixed modifications included carbamidomethylation of cysteine residues and methionine oxidation. N-terminal protein acetylation was set as a variable modification. Data analysis was performed using MaxQuant 1.6.0.1 software, including the Andromeda search engine for protein identification against the UniProt database.

The mass spectrometry proteomics data were deposited to the ProteomeXchange Consortium (http://proteomecentral.proteomexchange.org) via the PRIDE partner repository[11] with the dataset identifier PXD074176 and the title "*Mesoporous Silica Nanoparticles-Based Formulations for Enhanced Oral Delivery of Peptide Drugs: A Case Study on Insulin*".

Lucifer Yellow assay. Lucifer Yellow permeability was assessed to evaluate monolayer integrity as previously reported.[8,12] After 24 h of incubation of the cell culture inserts with particle suspensions (**PO_3-MSN(Ins)** or **PO_3-MSN**) or non-confined insulin (**Ins$^{(+)}$**), both apical and basolateral compartments were washed with Hank's balanced salt solution (HBSS) containing 25 mM D-glucose, 20 mM HEPES, 1.25 mM $CaCl_2$, and 0.5 mM $MgCl_2$ (pH 7.4). Subsequently, 0.5 mL of a 0.1 mg·mL^{-1} Lucifer Yellow CH di-lithium salt solution in HBSS and 1.5 mL of HBSS were added to the apical and basolateral compartments, respectively. After 1 h incubation at 37°C, the fluorescence of the basolateral medium was measured in triplicate (excitation: 485 nm, emission: 535 nm) using a plate reader (CYTATON 5762, BioTek Instruments, Winooski, VT, USA) and compared with the fluorescence of pure Lucifer Yellow stock

solution. Fluorescence values from the sample wells were normalized to those of pure Lucifer Yellow after subtraction of the HBSS blank and expressed as a percentage of permeability.

CellTiter-Blue (CTB) assay for MCF-7 breast cancer cells. Viability of MCF-7 cells was evaluated with the CTB assay after treatment with aliquots taken from the basolateral compartments of cell culture inserts cultured with the Caco-2/HT29-MTX-E12 intestinal model (**PO_3-MSN(Ins)**, **PO_3-MSN**, and non-confined **Ins$^{(+)}$** treatments, or medium from non-treated intestinal cells) in combination with either standard glucose (25 mM, Glu^{25mM}) or high glucose (50 mM, Glu^{50mM}) concentrations. The assay relies on the ability of metabolically active cells to reduce the redox dye resazurin into the fluorescent product resorufin. Non-viable cells lack metabolic activity and therefore do not produce a fluorescent signal, allowing cell viability to be directly correlated with resorufin fluorescence.[13] After 48 h of incubation with sample treatments (100 µL/well), the cell media was aspirated, and a 1:10 dilution of *CellTiter-Blue*® reagent (Promega) in DMEM without phenol red was added to the MCF-7 cells, followed by a 4 h incubation at 37°C.[14] The fluorescence of the supernatants was measured using a plate reader (CYTATON 5762, BioTek Instruments, Winooski, VT, USA) with excitation at 560 nm and emission at 590 nm. Resorufin fluorescence values were normalized to solvent controls (HBSS buffer), and viability was expressed as a percentage relative to the control MCF-7 cells incubated in standard conditions (complete MCF-7 medium supplemented with insulin, **Ins$^{(+)}$**, 0.1 µg/mL^{-1}, 17 nM). The experiments were performed in three independent biological replicates ($N = 3$), with technical duplicates for each experimental condition.

Protocol for tight junction protein immunostaining. After 6 h of treatment with **PO_3-MSN(Ins^{Flu})**, the Caco-2/HT29-MTX-E12 cells were washed twice with DPBS (250 µL/well), then fixed with MeOH/AcOH (90:10) and incubated overnight at 4°C. Thereafter, cells were permeabilized with 0.2% Triton X-100 in phosphate-buffered saline (PBS-A) for 15 min, and non-specific binding sites were blocked with 2% donkey serum for 1 h at room temperature. Cells were then incubated overnight at 4°C with the primary antibodies anti-ZO-1 (dil. 1:500 PBS-A) and anti-CLDN4 (dil. 1:500 PBS-A). Primary antibodies were removed by three consecutive washings with 0.05% Triton X-100 (washing buffer) for 10 min each. After two additional washing steps with PBS-A for 5 min, the secondary antibodies Alexa Fluor™ 647 donkey anti-goat IgG (H+L) (dil. 1:1000 PBS-A) and Alexa Fluor™ 568 donkey anti-rabbit IgG (H+L) (dil. 1:1000 PBS-A) were applied. The cells were then incubated in the dark at room temperature for 1.5 h. After three washing steps with 0.05% Triton X-100 and 2 washing steps with PBS-A, cells were incubated with MeOH/AcOH (90:10) for 15 min at room temperature as a post-fixation step. The cells were subsequently washed with PBS-A, and the fixative was quenched with glycine (100 mM in PBS-A). The cells were then embedded in a mounting medium containing DAPI and kept at 4°C until imaging.

MSN labeling for assessing particle biodistribution in intestinal mouse tissues. Phosphonated MSN (**PO_3-MSN**) were labeled with Alexa Fluor™ 647 (**PO_3-MSN^{AF}**) and administered by oral gavage to diabetic mice to evaluate MSN distribution in the ileum and colon tissues. First, an AF–APTES silane was synthesized (Scheme S4) by covalent coupling of Alexa Fluor™ 647 (AF) succinimidyl ester with APTES.[15] Briefly, 245 µL of Alexa Fluor™ 647 succinimidyl previously dissolved in DMSO (1.9 mM, 1.5 eq.) were combined with 5 µL of an APTES solution in DMSO (62 mM, 1.0 eq.). The reaction mixture was kept in the dark for 24 h. In parallel, 100 mg of **PO_3-MSN** were degassed overnight at 80°C and dispersed in 30 mL of anhydrous toluene at 50°C under stirring (700 rpm). Subsequently, the AF–APTES stock solution (50 µL, $6.2 \cdot 10^{-4}$ mmol/g silica) was added to the **PO_3-MSN** dispersion, and grafting was

carried out overnight. The AF-labeled MSN (**PO_3-MSN^{AF}**) were collected by centrifugation (9,000 rpm,11,770 x *g*, 20 min), washed once with toluene and three times with ethanol, and dried at 35°C for 24 h.

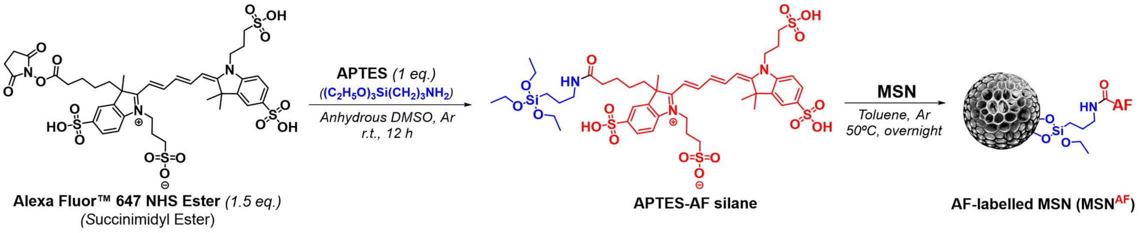

Scheme S4. Synthetic steps for the labeling of silica nanoparticles with Alexa Fluor™ 647 Succinimidyl Ester.

Material characterization. Transmission electron microscopy (TEM) images of the calcined **MSN** and functionalized particles (**PEG-MSN** and **PO_3-MSN**) were collected with a Philips CM200 microscope at an accelerating voltage of 200 kV. The samples for TEM imaging were prepared by dropping EtOH (4 µL) containing the suspended powder material on a holey carbon film-coated 300 mesh copper grid. For long-term stability studies of MSN in simulated gastrointestinal buffers, the particle suspensions were directly deposited onto copper grids and dried for 24 h prior to measurements.

Dynamic light scattering (DLS) analyses and zeta-potential measurements were performed on a Malvern DTS Nano Zetasizer at a 173° scattering angle (equilibrium time set at 3 min, 3 measurements for each sample). To ensure accurate calibration before zeta-potential measurements, a standard suspension (carboxylate-modified polystyrene latex microspheres) with a zeta potential of -40 (± 6) mV was used. The calcined and functionalized particles (**MSN**, **PEG-MSN**, and **PO_3-MSN**) were dispersed in H_2O at a concentration of 0.7 mg·mL^{-1}, vortexed (10 min), and sonicated (90 min) prior to the analysis. The hydrodynamic diameter and zeta potential of the colloidal dispersions were additionally measured at different pH values (pH range: 2.0-7.4) in both water and a saline medium (PBS buffer composition: Na_2HPO_4, KH_2PO_4, NaCl, and KCl).

N_2 physisorption isotherms were measured at -196°C (77 K) using an Autosorb-iQ3 sorption analyzer (Anton Paar, Boynton Beach, USA). Prior to analysis, the calcined **MSN** were outgassed for 10 h at 150°C; the functionalized particles (**PEG-MSN** and **PO_3-MSN**) for 10 h at 80°C; and the insulin-loaded particles (**MSN(Ins)**, **PEG-MSN(Ins)**, or **PO_3-MSN(Ins)**) for 20 h at 35°C. The specific surface area (S_{BET}) was determined using the Brunauer-Emmett-Teller (BET) equation[16] in the relative pressure range of 0.05-0.3 P/P_o. The total pore volume was determined at $P/P_0 = 0.95$. The pore size distributions (PSD) were estimated using the non-local density functional theory (NLDFT) method on the adsorption branch, considering an amorphous SiO_2 surface and a cylindrical pore model.[12] The calculations were performed using ASiQwin 5.2 software from Anton Paar Quantatech Inc.

Thermogravimetric (TGA) and differential scanning calorimetry (DSC) analyses were executed using a Netzsch STA-449 F3 Jupiter instrument from 25 to 800°C under airflow (20 mL·min^{-1}) as carrier gas with a heating rate of 10°C·min^{-1}. The mass losses (%) were estimated in the temperature range from 150 to 700°C.

Solid-state magic-angle-spinning nuclear magnetic resonance spectroscopy (MAS NMR) was performed on a Bruker Avance NEO 500 wide-bore system (Bruker BioSpin, Rheinstetten, Germany) at room temperature. A 4 mm triple-resonance MAS probe was used. Cross-polarization (CP) was employed using a ramped contact pulse, sweeping the proton radio frequency field from 50% to 100%. For ^{29}Si, the resonance frequency was 99.38 MHz, the MAS spinning speed was 8 kHz, and the CP contact time was 5 ms. The resonance frequency for ^{13}C NMR was 125.78 MHz, the MAS

rotor spinning was set to 14 kHz, and the CP contact time to 3ms. ^{31}P NMR experiments were performed at a frequency of 202.49 MHz with an MAS spinning speed of 14 kHz and a CP contact time of 3 ms. Chemical shifts (δ) were reported in ppm and referenced externally for ^{13}C to adamantane by setting its low field signal to 38.48 ppm, for ^{29}Si to 2,2-dimethyl-2-silapentane-5-sulfonate sodium salt (DSS) by setting the signal to 0 ppm, and for ^{31}P to $NH_4H_2PO_4$ by setting the signal to 0 ppm.

Attenuated total reflectance Fourier-transformed infrared (ATR-FTIR) spectra were recorded using a Bruker Vertex 70 FTIR spectrometer equipped with the Specac Golden Gate ATR accessory. The spectra were obtained by acquiring 72 scans at 4 cm^{-1} resolution over the mid-infrared range (4000-500 cm^{-1}). Before each measurement, a background spectrum was collected by acquiring 72 scans at a resolution of 4 cm^{-1}.

UV/Vis absorption spectra (200-800 nm) were collected on an Onda UV-30 SCAN spectrophotometer at room temperature to characterize the fluorescein and rhodamine-labeled materials. All samples were analyzed in 1 cm quartz cuvettes.

Wide-angle powder X-ray diffraction (WA-XRD) measurements were performed using a Panalytical Empyrean diffractometer (Malvern Panalytical, UK) in reflection geometry (Bragg-Brentano HD) with Cu $K\alpha_{1+2}$ radiation, operated at 45 kV and 40 mA, with a fixed divergence slit of 0.05 mm. The measurements were performed continuously with a step size 2θ of 0.013° and a time per step of 200 s.

Circular dichroism (CD) spectra of insulin released from MSN, sBL (50% succinylation), and native non-succinylated BL (0% succinylation) were recorded on a Chirascan Plus spectropolarimeter (Applied Photophysics) using a 1 mm path length microcuvette (Hellma Analytics). Each sample was prepared at an equivalent protein concentration of 200 $\mu g \cdot mL^{-1}$. Spectra were collected at 25°C over a wavelength range of 190–260 nm with a 1 nm step size. For each sample, five scans were averaged, and the corresponding background spectrum was subtracted. Raw data were exported using Pro-Data software and further processed in OriginPro 2024. CD data were expressed as the mean residue ellipticity (MRE, [θ], $deg \cdot cm^2 \cdot dmol^{-1}$), calculated according to Equation 1:

$$[\theta] = \frac{\theta \times MRW}{c \times l} \quad \textbf{[1]}$$

θ is the observed ellipticity (mdeg), MRW is the mean residue weight (molecular weight of the protein divided by the number of amino acid residues: 114 Da for insulin, 118 Da for sBL with 50% succinylation, and 113 Da for native BL), l is the optical pathlength (1 mm), and c is the protein concentration (200 $mg \cdot mL^{-1}$).[17] The MRE values at 208 nm obtained from CD spectra of insulin samples were used to estimate the α-helical content (%) of insulin released from **MSN(Ins)**, **PEG-MSN(Ins)**, and **PO_3-MSN(Ins)**, as well as non-confined insulin (control). The α-helical content (%) was calculated according to Equation 2:

$$\alpha\text{-helix } (\%) = -\frac{(\mathrm{MRE}_{208\,nm} - 4000)}{33000 - 4000} \times 100 \quad \textbf{[2]}$$

−33,000 $deg \cdot cm^2 \cdot dmol^{-1}$ and −4,000 $deg \cdot cm^2 \cdot dmol^{-1}$ correspond to the ellipticities of fully α-helical and random-coil conformations, respectively.[18]

The quantification of insulin from release studies and stability tests of tablet-based formulations was performed using a Vanquish Horizon UHPLC system (Thermo Fisher Scientific, Germering, Germany) coupled to an LTQ Orbitrap Velos mass spectrometer (Thermo Fisher Scientific, Bremen, Germany) equipped with an ESI source with the voltage set to 3 kV and an ion transfer capillary temperature of 380°C. Separation of analytes was carried out on a C_{18} analytical column Acclaim 120 (2.1 × 150 mm, 3 µm, Thermo Fisher Scientific, Vienna, Austria) at a flow rate of 0.4 $mL \cdot min^{-1}$.

The column oven temperature was set to 30°C, and the injection volume was 5 µL. The mobile phases were A: H_2O (0.1% (v/v) FA) and B: ACN (0.1% (v/v) FA). A linear gradient method was applied as follows: 0.0–8.0 min (5–53% B), 8.0–8.5 min (53–95% B), 8.5–12.5 min (95% B), 12.5–13.0 min (95–5% B). Re-equilibration was done from 13–17 min. Quantification of insulin released from powder samples without sBL, as well as insulin present in the basolateral compartment of cell culture inserts, was performed under the same conditions described above, but using a Vanquish Core HPLC system coupled to an Orbitrap Exploris 120 mass spectrometer equipped with an ESI source (Thermo Fisher Scientific). The full-scan mass spectra were acquired in positive mode in the range of 400–2000 m/z at a resolution of 60,000. Insulin concentration was calculated after peak integration (retention time: 5.3 min, m/z: 1161.7068-1163.9657) and interpolation in the calibration line (standard concentration: 0.01-6.0 $\mu g \cdot mL^{-1}$). The calibration standards used in release and stability tests under simulated gastrointestinal conditions were prepared in buffers at pH 1.2 or 7.4. For quantification of insulin permeability through cell culture inserts, standards were prepared in the complete culture medium used for Caco-2/HT29-MTX-E12 cells and processed under the same conditions as the samples collected from the basolateral compartments. This included the addition of pre-cooled MeOH/AcOH (90:10 v/v, -20°C), storage at -20°C for at least 1 h, and centrifugation (12,000 rpm, 13,800 x *g*, 20 min) prior to HPLC–MS analysis.

HR-ESI-MS spectra of labeled insulin (**Ins^{Flu}**) were obtained on a maXis UHR ESI-Qq-TOF mass spectrometer (Bruker Daltonics, Bremen, Germany) in positive ion mode in the range of 100-3000 m/z. The samples were introduced *via* direct infusion in ACN/H_2O (1:1, + 0.1% FA) at a flow rate of 3 $\mu L \cdot min^{-1}$. The capillary voltage was set at ± 4,500 V, the dry gas flow at 4 $L \cdot min^{-1}$ (nitrogen), and the dry temperature at 180°C. The sum formulas of the detected ions were determined using Bruker Compass DataAnalysis 4.1 based on the mass accuracy ($\Delta m/z \leq 5$ ppm) and isotopic pattern matching (SmartFormula algorithm). Charge state determination and deconvolution of ESI mass-to-charge ratio spectra and determination of the molecular mass of the labeled insulin (**Ins^{Flu}**) were performed using the MagTran software program.[19]

FIGURES AND TABLES

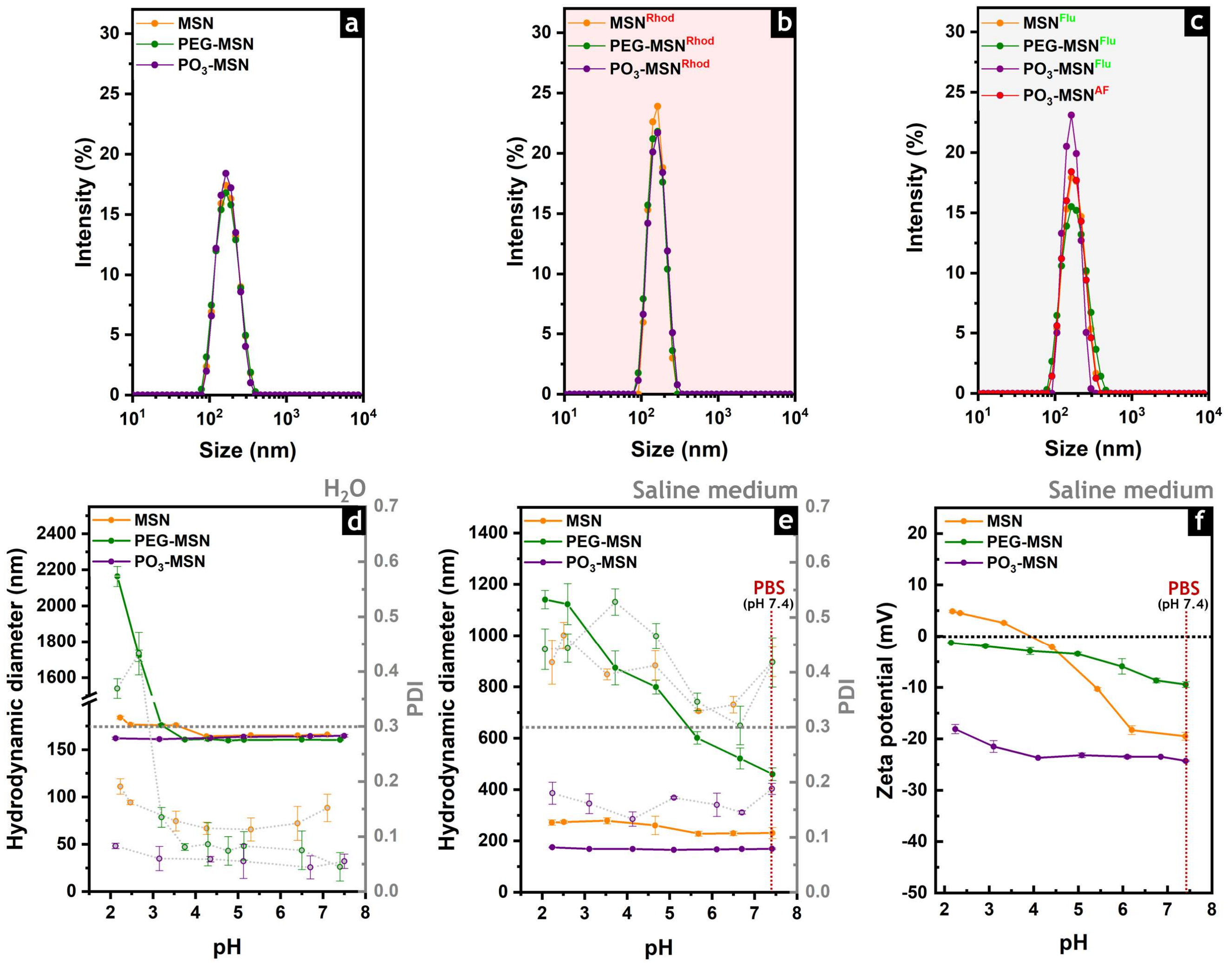

Figure S1. Hydrodynamic diameters and pH-dependent colloidal stability and zeta potential of MSN. (a) Dynamic light scattering (DLS) profiles of (a) calcined **MSN** and functionalized particles (**PEG-MSN** and **PO_3-MSN**), (b) rhodamine-labeled MSN (**MSN^{Rhod}**, **$PEG\text{-}MSN^{Rhod,}$** and **PO_3-MSN^{Rhod}**), and (c) fluorescein-labeled MSN (**MSN^{Flu}**, **$PEG\text{-}MSN^{Flu}$**, and **PO_3-MSN^{Flu}**) and AF-labeled phosphonated MSN (**PO_3-MSN^{AF}**), dispersed in H_2O (0.7 mg·mL^{-1}). Hydrodynamic diameters and respective polydispersity indexes (PDI) of **MSN**, **PEG-MSN**, and **PO_3-MSN**, measured in (d) H_2O or in (e) saline medium (PBS buffer composition: Na_2HPO_4, KH_2PO_4, NaCl, and KCl), between pH 2 and 7.4. (f) Zeta potential of **MSN**, **PEG-MSN**, and **PO_3-MSN**, measured in the saline medium over pH 2–7.4 (PBS).

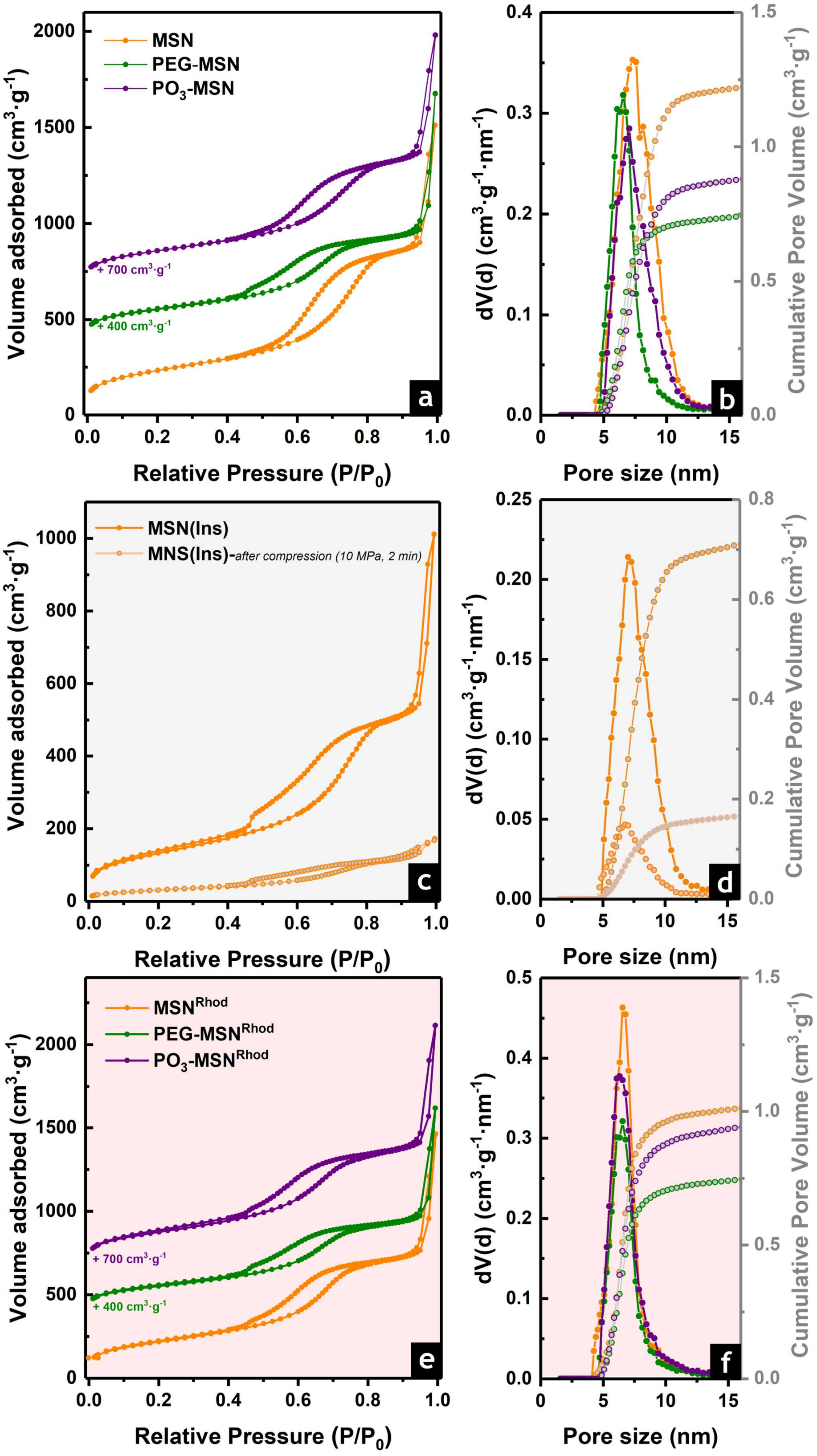

Figure S2. Characterization of the porosity of MSN. N_2-physisorption isotherms (77 K) of (a) **MSN**, **PEG-MSN**, and **PO_3-MSN**, (c) insulin-loaded MSN (**MSN(Ins)**) before and after compression (10 MPa, 2 min) to form tablets without sBL, and (e) rhodamine-labeled **MSN^{Rhod}**, **$PEG\text{-}MSN^{Rhod}$**, and **$PO_3\text{-}MSN^{Rhod}$**. (b, d, f) Respective NLDFT pore size distributions (PSD) and cumulative pore volume plots.

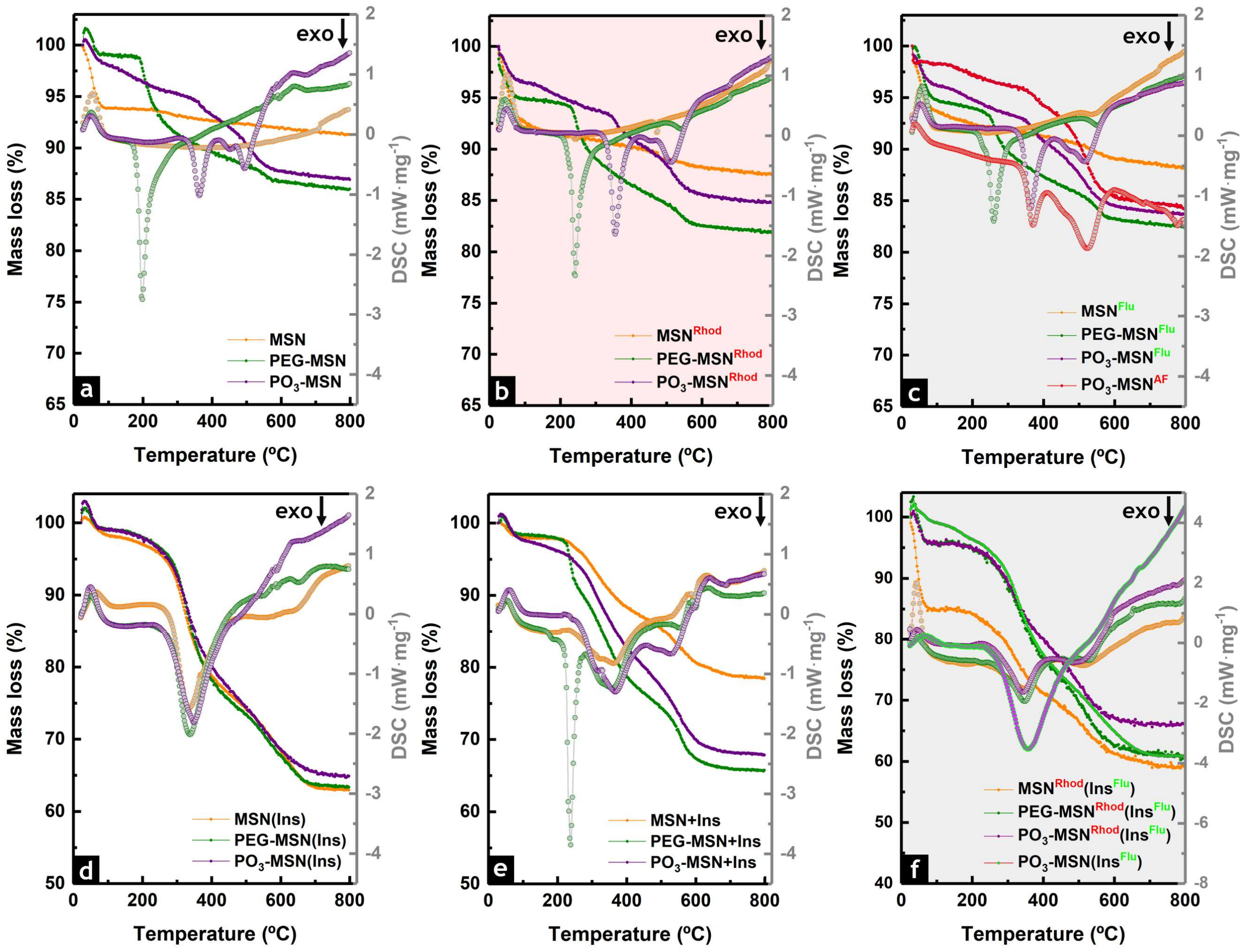

Figure S3. Thermogravimetric (TGA) and differential scanning calorimetry (DSC) analyses of the materials. (a) calcined and functionalized MSN (**MSN**, **PEG-MSN**, and **PO_3-MSN**), (b) rhodamine-labeled MSN (**MSN^{Rhod}**, **PEG-MSN^{Rhod}**, and **PO_3-MSN^{Rhod}**), (c) fluorescein-labeled MSN (**MSN^{Flu}**, **PEG-MSN^{Flu}**, and **PO_3-MSN^{Flu}**), and AF-labeled phosphonated MSN (**PO_3-MSN^{AF}**), (d) insulin-loaded MSN (**MSN(Ins)**, **PEG-MSN(Ins)**, and **PO_3-MSN(Ins)**), (d) physical mixtures of MSN and insulin (20 wt% equivalent to insulin), (e) fluorescein-labeled insulin (**Ins^{Flu}**) loaded on rhodamine-labeled particles (**MSN^{Rhod}(Ins^{Flu})**, **PEG-MSN^{Rhod}(Ins^{Flu})**, and **PO_3-MSN^{Rhod}(Ins^{Flu})**) and on phosphonated MSN (**PO_3-MSN(Ins^{Flu})**).

Table S1. Physico-chemical parameters of the functionalized, insulin-loaded, and/or labeled MSN.

Material	S_{BET}[a] ($m^2 \cdot g^{-1}$)	Pore size[a] (nm)	Pore volume[a] ($cm^3 \cdot g^{-1}$)	Particle size[b] (nm)	Zeta-potential (mV)	Mass loss[d] (%)
**MSN**	828	7.3	2.00	160 (± 1)<br>160 (± 1) [c]	-34 (± 1)<br>-19 (± 1) [c]	-
**PEG-MSN**	560	6.6	1.48	165 (± 1)<br>164 (± 1) [c]	-30 (± 1)<br>-12 (± 1) [c]	+ 11.3<br>(PEG-silane)
**PO_3-MSN**	666	6.1	1.73	164 (± 1)<br>162 (± 1) [c]	-38 (± 1)<br>-39 (± 1) [c]	+ 10.1<br>(THMP)
*Rhodamine-labeled MSN (**MSN^{Rhod}**)*						
**MSN^{Rhod}**	782	6.6	1.82	156 (± 1)<br>171 (± 3) [c]	-28 (± 1)<br>-13 (± 5) [c]	+ 2.6 (rhodamine)
**PEG-MSN^{Rhod}**	560	6.6	1.45	162 (± 1)<br>192 (± 1) [c]	-27 (± 1)<br>-6 (± 1) [c]	+ 3.1 (rhodamine)
**PO_3-MSN^{Rhod}**	588	7.0	1.64	157 (± 1)<br>160 (± 1) [c]	-39 (± 2)<br>-36 (± 3) [c]	+ 2.4 (rhodamine)
*Fluorescein-labeled MSN (**MSN^{Flu}**)*						
**MSN^{Flu}**	-	-	-	169 (± 1)<br>164 (± 1) [c]	-24 (± 1)<br>-19 (± 1) [c]	+ 3.0 (fluorescein)
**PEG-MSN^{Flu}**	-	-	-	166 (± 1)<br>174 (± 2) [c]	-25 (± 2)<br>-17 (± 1) [c]	+ 2.9 (fluorescein)
**PO_3-MSN^{Flu}**	-	-	-	160 (± 1)<br>161 (± 1) [c]	-37 (± 1)<br>-37 (± 1) [c]	+ 3.1 (fluorescein)
*AF-labeled MSN (**MSN^{AF}**)*						
**PO_3-MSN^{AF}**	-	-	-	165 (± 1)	-38 (± 1)	+ 2.0 (Alexa Fluor)
*Insulin-loaded MSN (**MSN(Ins)**)*						
**MSN(Ins)**	483	7.0	1.31	-	-	+ 24.9 (Insulin)
**MSN(Ins)-*compressed***[e]	113	6.8	0.25	-	-	-
**PEG-MSN(Ins)**	362	6.1	1.09	-	-	+ 22.4 (Insulin)
**PO_3-MSN(Ins)**	218	7.0	0.80	-	-	+ 22.7 (Insulin)
***Ins^{Flu}** loaded on rhodamine-labeled MSN (**MSN^{Rhod}(Ins^{Flu})**)*						
**MSN^{Rhod}(Ins^{Flu})**	-	-	-	-	-	+ 21.3 (Ins^{Flu})
**PEG-MSN^{Rhod} (Ins^{Flu})**	-	-	-	-	-	+ 21.9 (Ins^{Flu})
**PO_3-MSN^{Rhod} (Ins^{Flu})**	-	-	-	-	-	+ 18.5 (Ins^{Flu})
***Ins^{Flu}** loaded on MSN (**MSN(Ins^{Flu})**)*						
**PO_3-MSN(Ins^{Flu})**	-	-	-	-	-	+ 27.8 (Ins^{Flu})

[a] Porosity and textural properties (specific surface area S_{BET}, pore volume, and pore size) were obtained from the N_2-physisorption analysis (77 K). [b] The particle size (hydrodynamic diameter) was measured by DLS. [c] Particle size and zeta-potential measured at pH 4. [d] The mass losses (%) were obtained from TGA and were estimated in the temperature range from 150 to 700°C. The increases in mass losses after consecutive synthetic steps (+ wt%) were calculated using the mass loss (%) corresponding to the respective precursors as a reference. [e] To evaluate the effect of tableting on the overall MSN porosity, insulin-loaded MSN (**MSN(Ins)**) were compressed using a single-punch press (PerkinElmer, UK) at 10 MPa for 2 min, following the same conditions applied for the preparation of the corresponding tablet formulation (**sBL[MSN(Ins)]**), but without sBL.

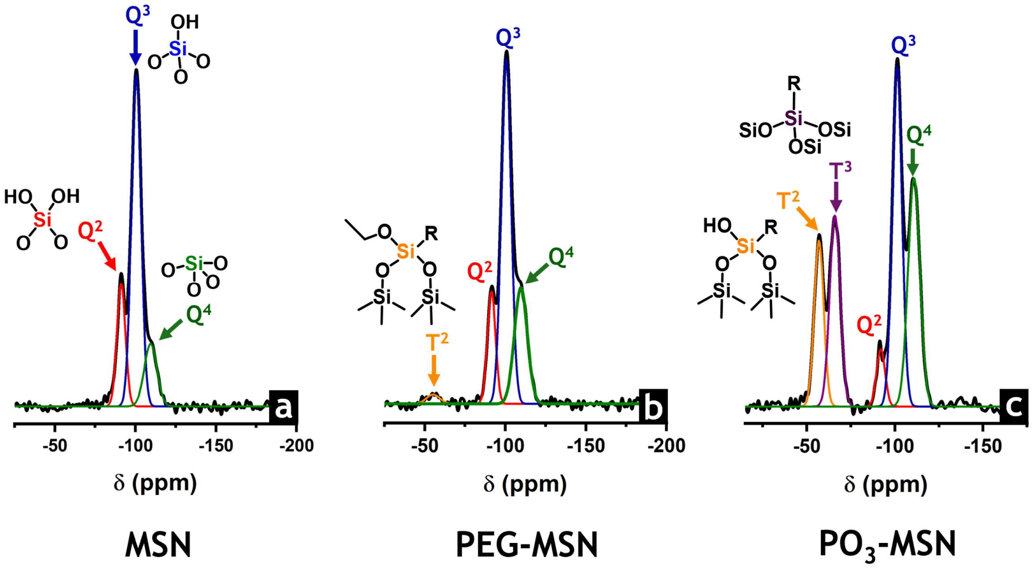

Figure S4. Solid-state ^{29}Si CP/MAS NMR spectra of MSN. (a) **MSN**, (b) **PEG-MSN**, and (c) **PO3-MSN** ("**R**" = chemical functions from the PEG-silane or THMP grafted on **PEG-MSN** or **PO3-MSN**, respectively).

Table S2. Assignment of the signals of the solid-state ^{29}Si CP/MAS NMR spectra.

Signal [20,21]	δ (ppm)		
	MSN	PEG-MSN	PO3-MSN
(≡SiO)$_2$Si(OH)R (T^2)	-	-56.3	-57.6
(≡SiO)$_3$SiR (T^3)	-	-	-65.6
Si(OH)$_2$(O–)$_2$ (Q^2)	-91.8	-92.3	-91.5
Si(OH)(O–)$_3$ (Q^3)	-100.5	-101.9	-102.2
Si(O–)$_4$ (Q^4)	-111.0	-110.1	-110.7

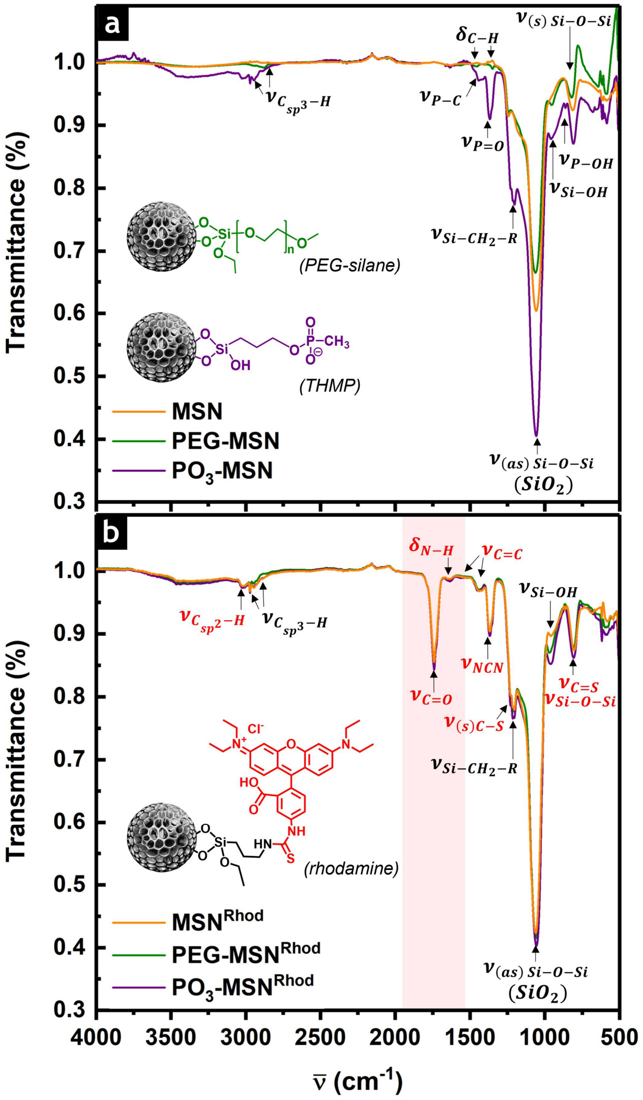

Figure S5. ATR-FTIR spectra of MSN. (a) Calcined and functionalized particles (**MSN**, **PEG-MSN**, and **PO_3-MSN**), and (b) rhodamine-labeled MSN (**MSN^{Rhod}**, **$PEG\text{-}MSN^{Rhod}$**, and **$PO_3\text{-}MSN^{Rhod}$**).

Table S3. Signal assignment of the ATR-FTIR spectra of functionalized and rhodamine-labeled MSN.

Signal[22–25]	$\bar{\nu}$ (cm^{-1})			
	MSN	PEG-MSN	PO_3-MSN	rhodamine-labeled MSN[§]
$\nu_{C_{sp^2}-H}$ *(aromatic)*	-	-	-	3024; 3006
$\nu_{C_{sp^3}-H}$ *(alkyl)*	-	2946; 2879	2970; 2945; 2888	*
$\nu_{C=O}$				1740
δ_{N-H}	-	-	-	1655
δ_{C-H} *(PEG-silane)*	-	1456; 1350	-	*
ν_{P-C} *(THMP)*	-	-	1444	*
$\nu_{P=O}$ *(THMP)*	-	-	1368	*
$\nu_{C=C}$ *(aromatic ring)*	-	-	-	1562; 1439; 1421
ν_{NCN}	-	-	-	1366
$\nu_{(s)C-S}$	-	-	-	1228
ν_{Si-CH_2-R} *(THMP)*	-	-	1216; 1205	*
$\nu_{(as)\,Si-O-Si}$ *(SiO_2)*	1059	1065	1058	*
ν_{Si-OH} *(SiO_2)*	956	953	959	*
ν_{P-OH} *(THMP)*	-	-	863	*
$\nu_{C=S}$	-	-	-	807
$\nu_{(s)\,Si-O-Si}$ *(SiO_2)*	815	819	810	*

§ The ATR-FTIR spectra of the rhodamine-labeled MSN (**MSN^{Rhod}**, **PEG-MSN^{Rhod},** and **PO_3-MSN^{Rhod}**) exhibited characteristic signals corresponding to the thiourea derivative formed after functionalization with the Rhod-APTES silane. *Signals of the respective precursors (**MSN**, **PEG-MSN**, and **PO_3-MSN**) overlapped with the peaks of the labeled fluorophore.

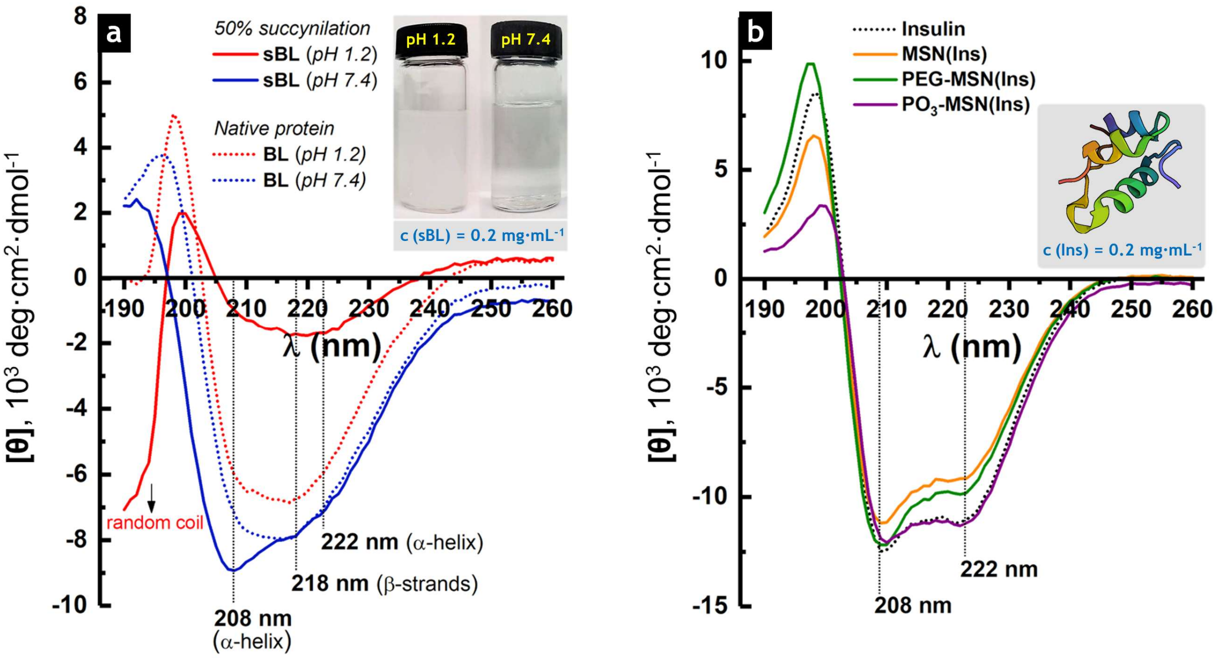

Figure S6. Circular dichroism spectra of sBL, native BL, and insulin released from MSN(Ins)-based formulations. (a) pH-dependent secondary structure of sBL (50% succinylation, solid lines) compared with the native protein (dotted lines). The inset images show sBL suspensions at pH 1.2 and pH 7.4, illustrating protein aggregation under acidic conditions. (b) Retention of the secondary structure of insulin released from loaded MSN (**MSN(Ins)**, **PEG-MSN(Ins)**, and **PO_3-MSN(Ins)**) after 2 h of incubation in buffer at pH 7.4 and 37°C, compared with non-encapsulated insulin (control, black dotted line). The schematic representation of the insulin structure was created with BioRender.com.

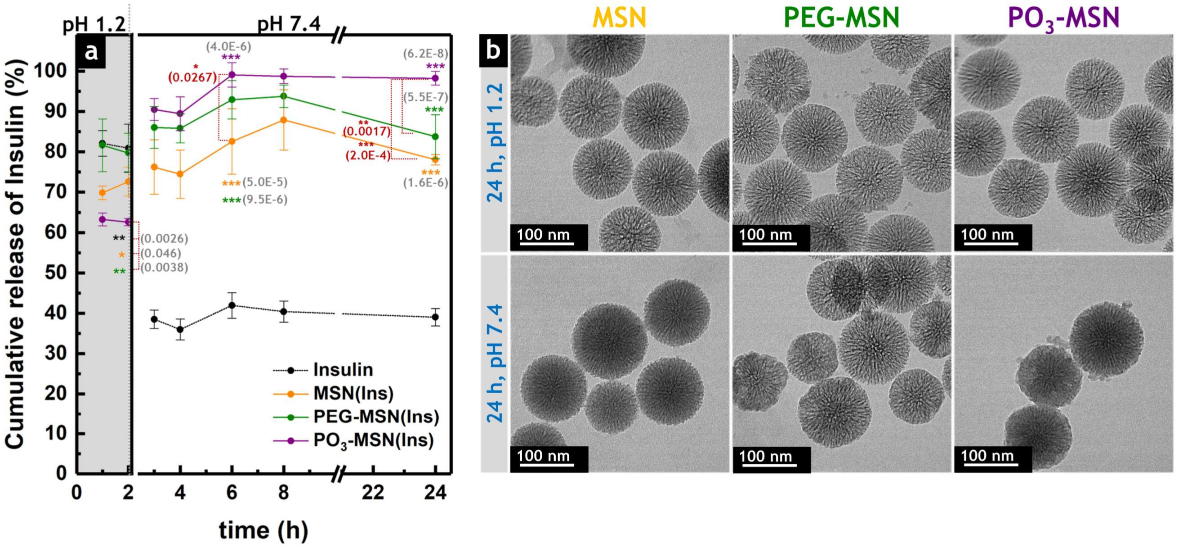

Figure S7. Insulin release from MSN-based formulations without sBL and long-term stability of MSN carriers in buffers at pH 1.2 and 7.4. (a) Cumulative insulin release from insulin-loaded MSN (**MSN(Ins)**, **PEG-MSN(Ins)**, and **PO_3-MSN(Ins)**) in powder form, without tableting with sBL. Data are presented as the mean ± standard deviation ($N = 3$). One-way ANOVA followed by Fisher's test was used to assess significant differences between MSN-based formulations and non-encapsulated insulin (control, dotted line), which are indicated as *(p < 0.05), **(p < 0.01), and ***(p < 0.001). (b) TEM images of insulin-loaded MSN (**MSN(Ins)**, **PEG-MSN(Ins)**, and **PO_3-MSN(Ins)**) after 24 h-release at 37°C under pH 1.2 and pH 7.4.

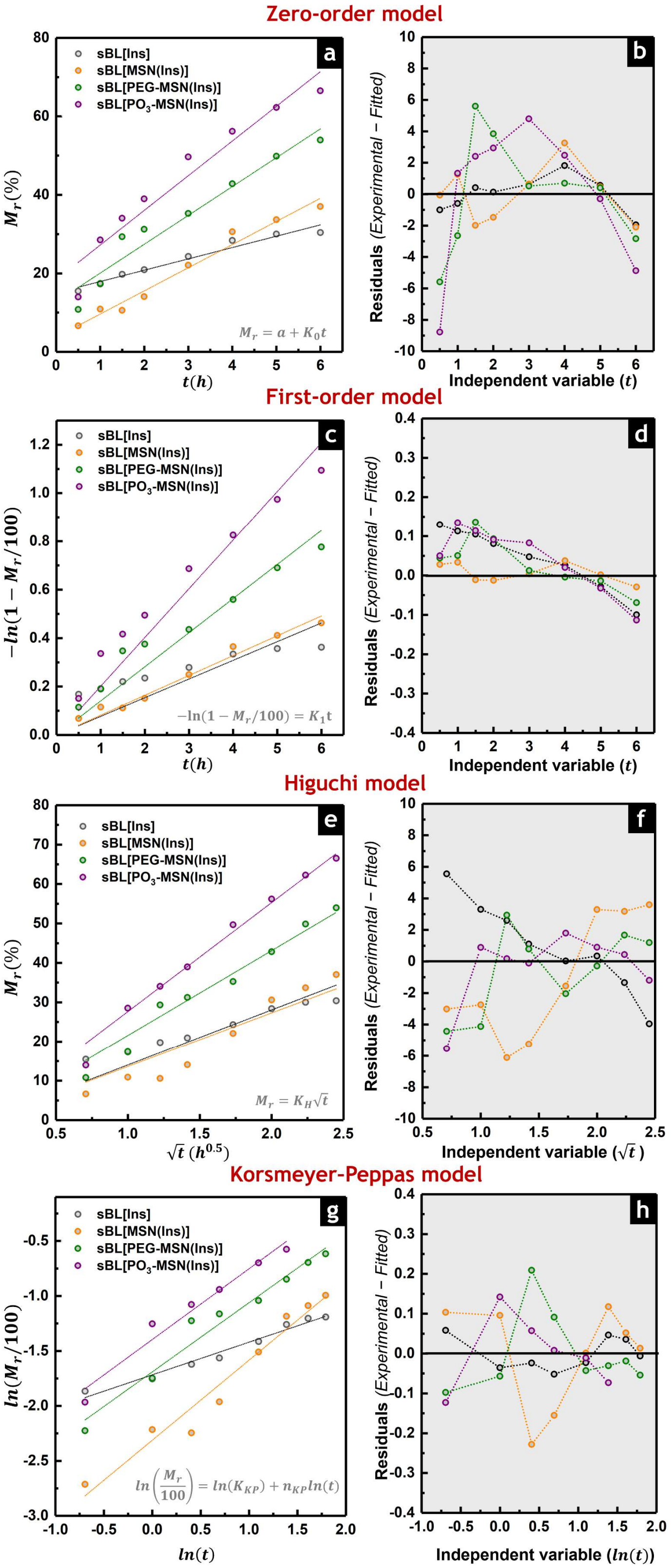

Figure S8. Kinetics and mechanistic modeling of insulin release from tablet formulations under intestinal conditions. Linearized kinetic fits (left) and corresponding residual plots (right) for zero-order (a,b), first-order (c,d), Higuchi (e,f), and Korsmeyer–Peppas (g,h) models describing intestinal-phase insulin release from sBL-tablet formulations containing non-confined insulin (**sBL[Ins]**, control) or insulin-loaded MSN (**sBL[MSN(Ins)]**, **sBL[PEG-MSN(Ins)]**, or **sBL[PO$_3$-MSN(Ins)]**). Residuals represent experimental minus fitted values. For kinetic analysis, the amount of insulin remaining after 2 h of release at pH 1.2 was used to recalculate the percentage of insulin released at pH 7.4 (M_r), and time was corrected to account exclusively for the intestinal phase (pH 7.4). Model fitting was restricted to the early intestinal release window (0.5–6 h).

Table S4. Kinetic parameters for intestinal-phase insulin release from sBL-based tablets after transference from pH 1.2 to pH 7.4.

**Model**	**Zero-order**[26]			**First-order**[27]		**Higuchi**[28]		**Korsmeyer–Peppas**[29]			
**Equation**[a]	$M_r(\%) = a + K_0 t$			$\frac{M_r}{100} = 1 - e^{-K_1 t}$		$M_r(\%) = K_H \sqrt{t}$		$\frac{M_r}{100} = K_{KP} t^{n_{KP}}$			
**Linear fits**[b]	$M_r\ vs. t$			$-ln\left(1 - \frac{M_r}{100}\right) vs. t$		$M_r\ vs. \sqrt{t}$		$ln\left(\frac{M_r}{100}\right)\ vs. \ln(t)$			
**Tablet formulations**	$\boldsymbol{a}$ (%)	$\boldsymbol{K_0}$ ($\% \, h^{-1}$)	$\boldsymbol{R^2}$	$\boldsymbol{K_1}$ (h^{-1})	$\boldsymbol{R^2}$	$\boldsymbol{K_H}$ ($\%h^{-0.5}$)	$\boldsymbol{R^2}$	$\boldsymbol{a}$ ln (K_{KP})	$\boldsymbol{K_{KP}}$ (h^{-n})	$\boldsymbol{n_{KP}}$ [c]	$\boldsymbol{R^2}$
**sBL[Ins]**	15	2.9	0.96050	0.077	0.90129	14.0	0.98534	-1.72	0.18 $h^{-0.30}$	0.30	0.97436
**sBL[MSN(Ins)]**	4	5.7	0.97599	0.082	0.99301	13.6	0.97319	-2.31	0.10 $h^{-0.73}$	0.73	0.9601
**sBL[PEG-MSN(Ins)]**	13	7.3	0.94072	0.141	0.98055	21.6	0.99493	-1.69	0.18 $h^{-0.63}$	0.63	0.96618
**sBL[PO3-MSN(Ins)]**	18	8.8	0.93597	0.201	0.98359	27.6	0.99789	-1.40	0.25$h^{-0.64}$	0.64	0.96418

[a] M_r is the percentage of insulin released at time t, normalized to the amount of insulin remaining after transfer of the tablets from pH 1.2 to pH 7.4; t is the time after transfer to pH 7.4 (h); K_0 is the zero-order release constant (%·h⁻¹); K_1 is the first-order release constant (h^{-1}); K_H is the Higuchi diffusion constant ($\%\cdot h^{-1/2}$); n_{KP} is the release exponent; K_{KP} is the Korsmeyer–Peppas kinetic constant (h^{-n}); a is the intercept of the linear regression (fixed at 0 for the first-order and Higuchi models); and R^2 is the coefficient of determination (R-Square, COD) reported by OriginPro 2024 for the corresponding linear fits.

[b] Linear regression was performed using pH 7.4 release data only, with time corrected to account exclusively for the intestinal phase and restricted to the early intestinal release window (0.5–6 h). Release values (M_r%) were recalculated based on the amount of insulin remaining after the preceding 2 h release at pH 1.2.

[c] According to the Korsmeyer–Peppas model, $n_{KP} < 0.45$ indicates Fickian diffusion– or depletion-dominated release; $0.45 < n_{KP} < 0.89$ corresponds to anomalous (non-Fickian) transport involving combined diffusion and matrix relaxation, and $n_{KP} \approx 1$ is associated with Case-II transport or erosion-controlled release. The model is applicable only within the initial release regime (≤ 60% of cumulative release).[29]

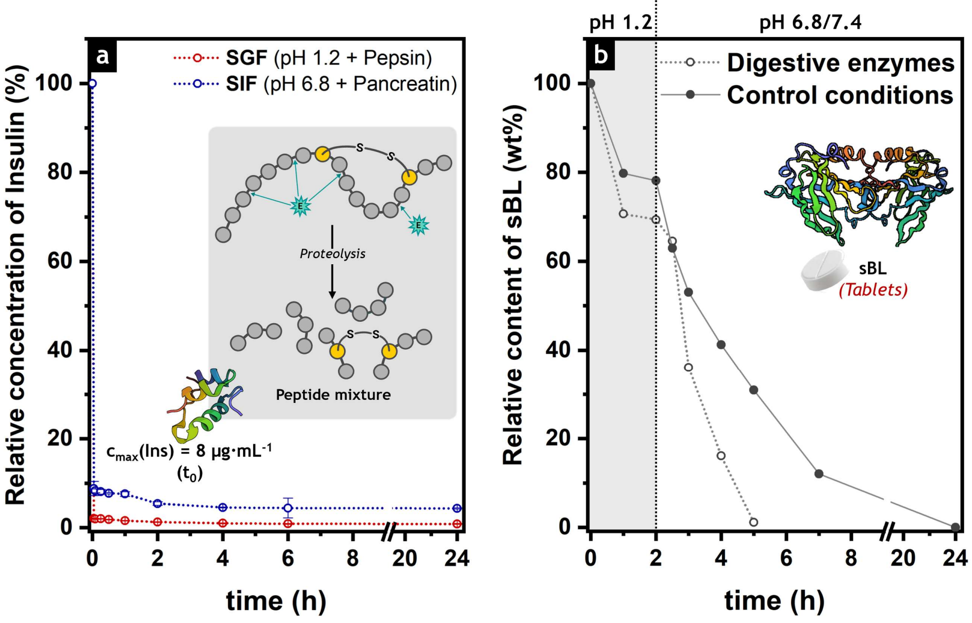

Figure S9. Insulin stability in the presence of digestive enzymes and dissolution test of sBL tablets. (a) Relative concentration of non-confined insulin over time when dispersed in SGF or SIF. Experiments were performed in triplicate, and data sets are presented as the mean ± standard deviation (*N* = 3). (b) Relative content of succinylated β-lactoglobulin (sBL, wt%) after placing the tablets for 2 h in pH 1.2 buffer and subsequently in pH 7.4 buffer (control conditions, straight line) or for 2 h in SGF (pH 1.2 + Pepsin) followed by SIG (pH 6.8 + Pancreatin), both containing digestive enzymes (dotted line). The schematic representations of the insulin and sBL structures were created using BioRender.com.

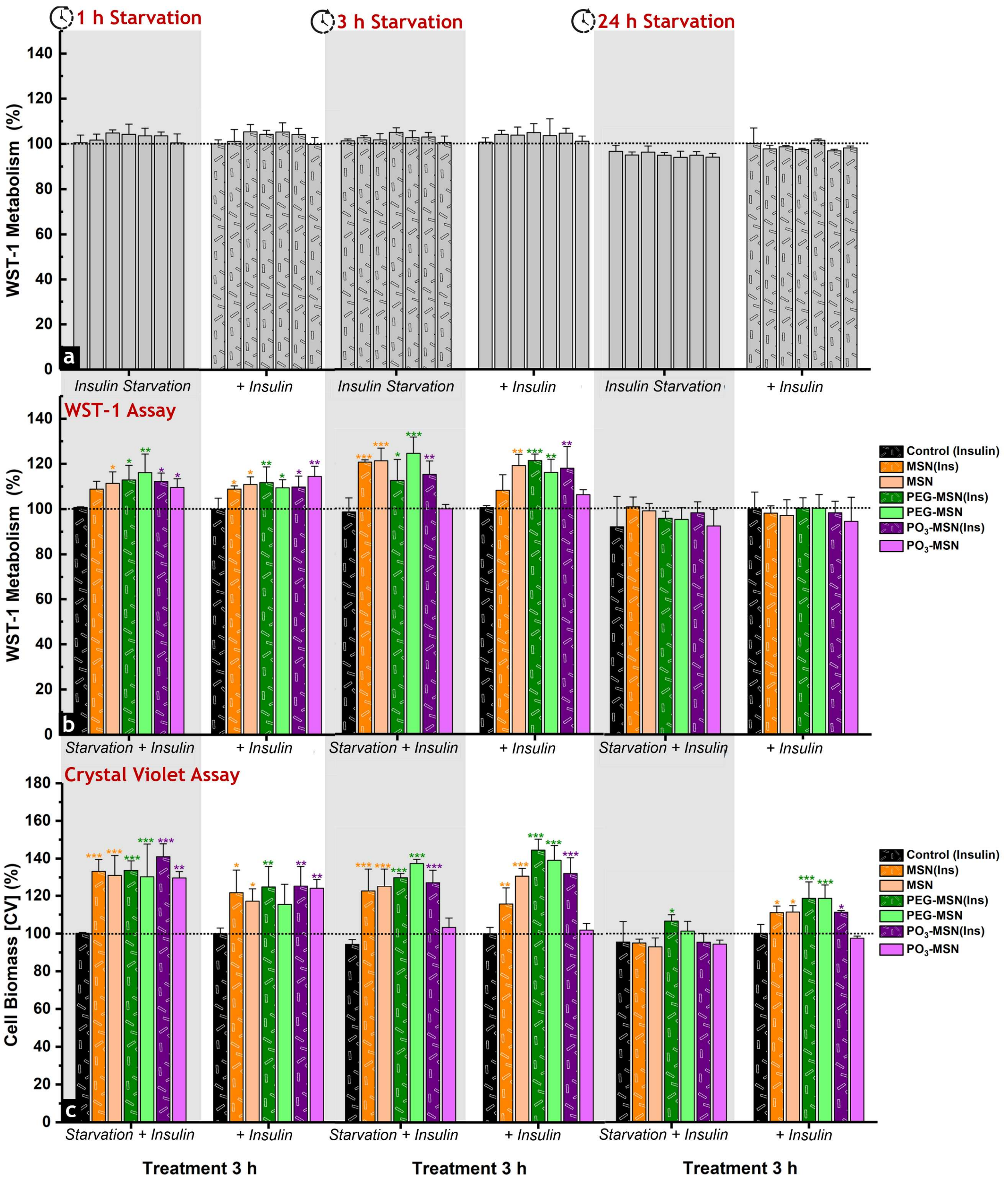

Figure S10. Cytotoxicity of "empty" MSN and insulin-loaded MSN. (a) WST-1 assay based on the incubation of HCEC-1CT cells for 1 h, 3 h, or 24 h in the absence (starvation conditions, **Ins**$^{(-)}$, gray background) or presence of insulin (10 μg mL^{-1}, 1.7 μM), prior to (b) 3 h-incubation with different treatments: commercial human insulin (non-confined insulin, 10 μg mL^{-1}, 1.7 μM), insulin-loaded-MSN (confined insulin, 10 μg mL^{-1}, 1.7 μM), and empty MSN (**Ins**$^{(-)}$). (c) Cell Biomass was measured *via* the Crystal Violet assay after incubation of cells in culture medium (in starvation or control conditions), followed by a 3 h-treatment with nanoparticle formulations. The values are expressed as percentages relative to the positive control (cell incubated with complete cell culture medium, **Ins**$^{(+)}$, 10 μg mL^{-1}, 1.7 μM). All data were obtained from three independent cell preparations, measured in technical duplicates, and shown as the mean ± standard deviation (N = 3). One-way ANOVA and Fisher Test expressing significant differences with respect to the positive control (**Ins**$^{(+)}$) are indicated with * ($p < 0.05$), ** ($p < 0.01$), or *** ($p < 0.001$).

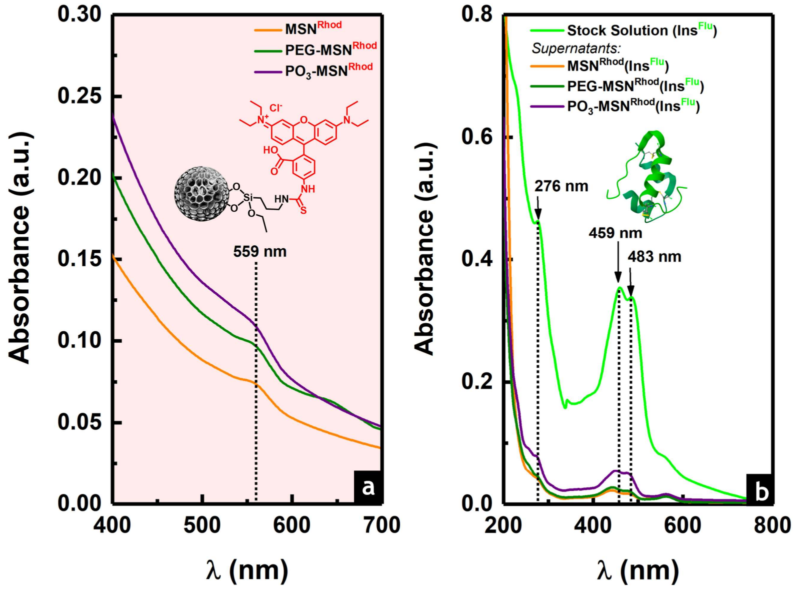

Figure S11. UV/Vis characterization of labeled materials. Spectra of (a) rhodamine-labeled MSN (**MSN^{Rhod}**, **PEG-MSN^{Rhod}**, and **PO_3-MSN^{Rhod}**) and (b) supernatants obtained after the loading of **Ins^{Flu}** (fluorescein-labeled insulin) on rhodamine-labeled MSN. The UV/Vis spectrum of the stock solution of **Ins^{Flu}** is represented in green (control, 20x dilution).

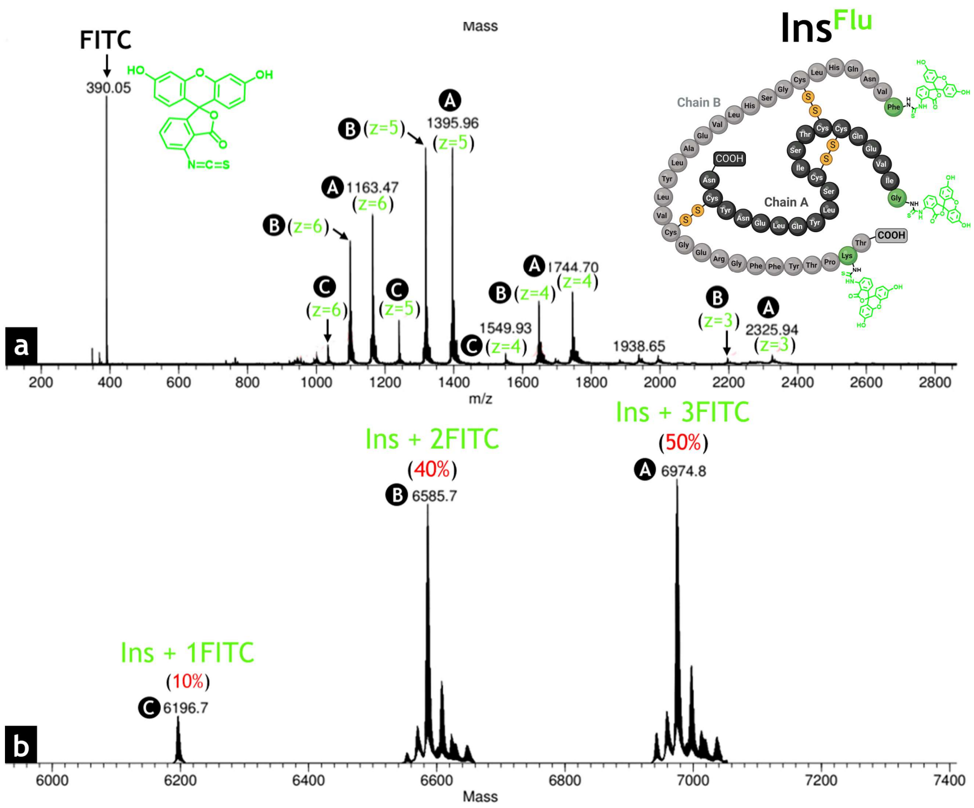

Figure S12. Structural MS characterization of the fluorescein-labeled insulin (Ins^{Flu}). (a) HR-ESI-MS spectrum of **Ins^{Flu}**. (b) Deconvoluted ESI mass spectrum of **Ins^{Flu}** showing peaks from molecular ions $(M+H)^+$ corresponding to one (mass = 6196.7; 10%, *C*), two (mass = 6585.7; 40%, *B*), or three (mass = 6974.8; 50%, *A*) molecules of FITC (mass = 390.05) grafted on native insulin (mass = 5806.7) after labeling. Deconvolution was performed using MagTran software. The schematic representation of the **Ins^{Flu}** structure was created with BioRender.com.

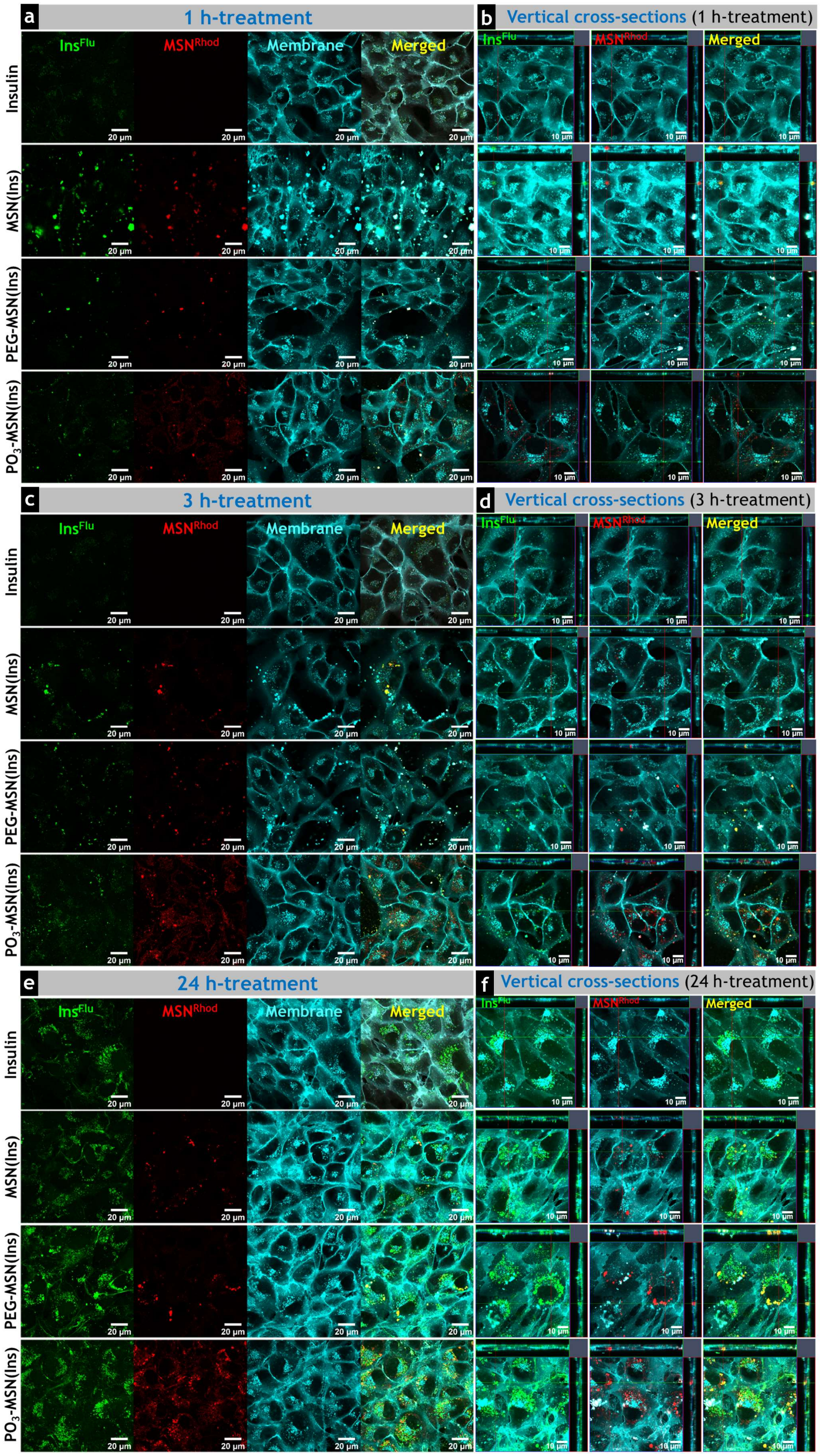

Figure S13. Live fluorescence imaging of HCEC-1CT cells incubated with insulin-loaded MSN ($MSN^{Rhod}(Ins^{Flu})$). Representative images upon incubation for (a) 1 h, (c) 3 h, and (e) 24 h at 37°C with $\mathbf{MSN^{Rhod}(Ins^{Flu})}$, $\mathbf{PEG\text{-}MSN^{Rhod}(Ins^{Flu})}$, $\mathbf{PO_3\text{-}MSN^{Rhod}(Ins^{Flu})}$, or fluorescein-labeled insulin ($\mathbf{Ins^{Flu}}$, control). The fluorescence signals from insulin and MSN were simultaneously detected and represented in green (Ins^{Flu}) and red (MSN^{Rhod}), respectively. The plasma membrane is depicted in cyan (CellMask™ Deep Red Plasma Membrane Stain). Scale bars stand for 20 µm. Respective vertical cross-sections of 3D cut stacks obtained from Z-stack imaging after (b) 1 h, (d) 3 h, and (f) 24 h-treatments. Scale bars stand for 10 µm.

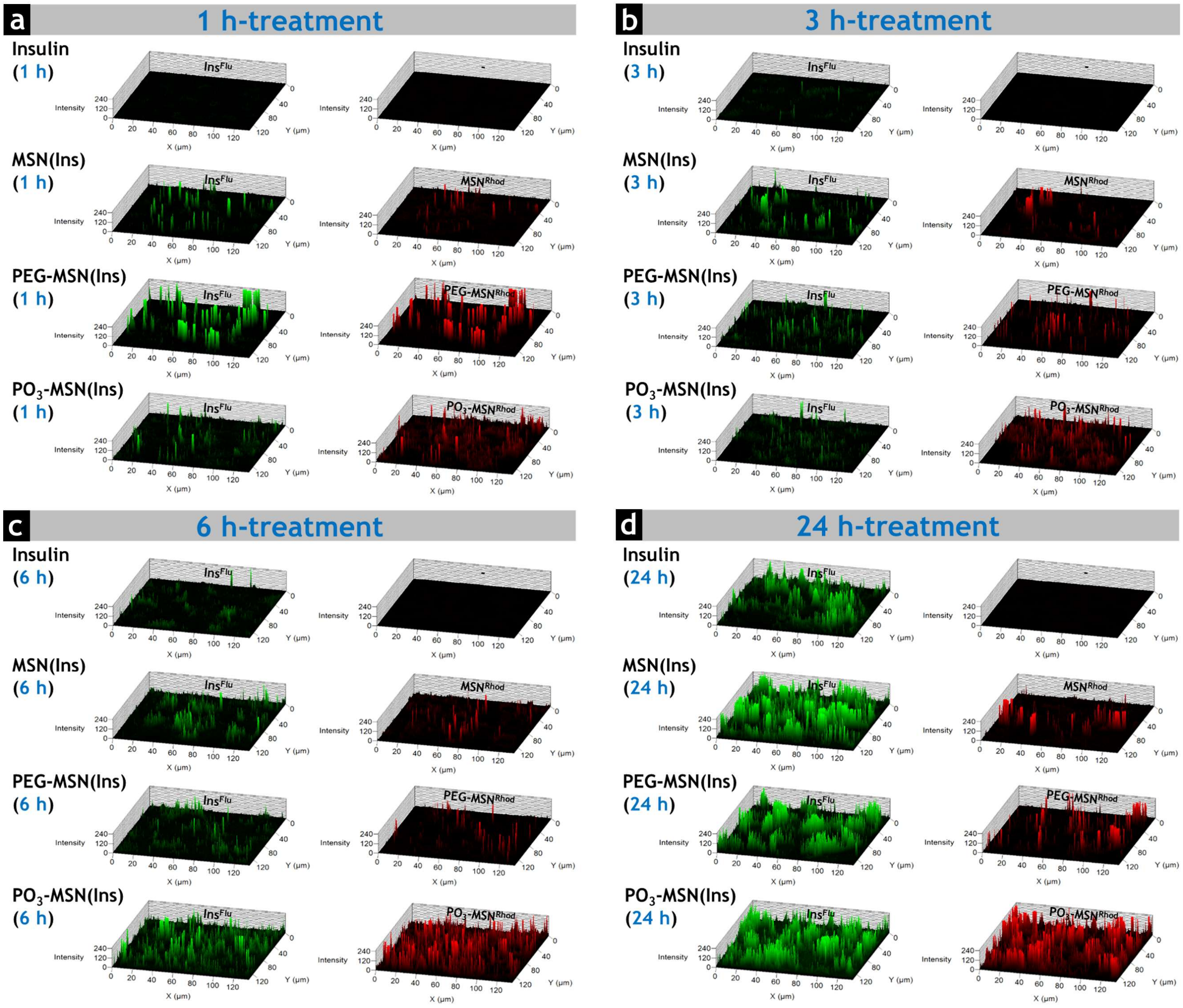

Figure S14. 2.5D views obtained from live cell fluorescence imaging. Representative images after treatment of HCEC-1CT cells for (a) 1 h, (b) 3 h, (c) 6 h, and (d) 24 h at 37°C with insulin-loaded MSN (**MSN^{Rhod}(Ins^{Flu})**, **PEG-MSN^{Rhod}(Ins^{Flu})**, or **PO_3-MSN^{Rhod}(Ins^{Flu})**) or non-confined insulin (**Ins^{Flu}**, control). The scale bar segmentation is 20 µm, and the fluorescence signals of FITC-labeled insulin (**Ins^{Flu}**) and Rhod-labeled particles (**MSN^{Rhod}**) are represented in green and red, respectively.

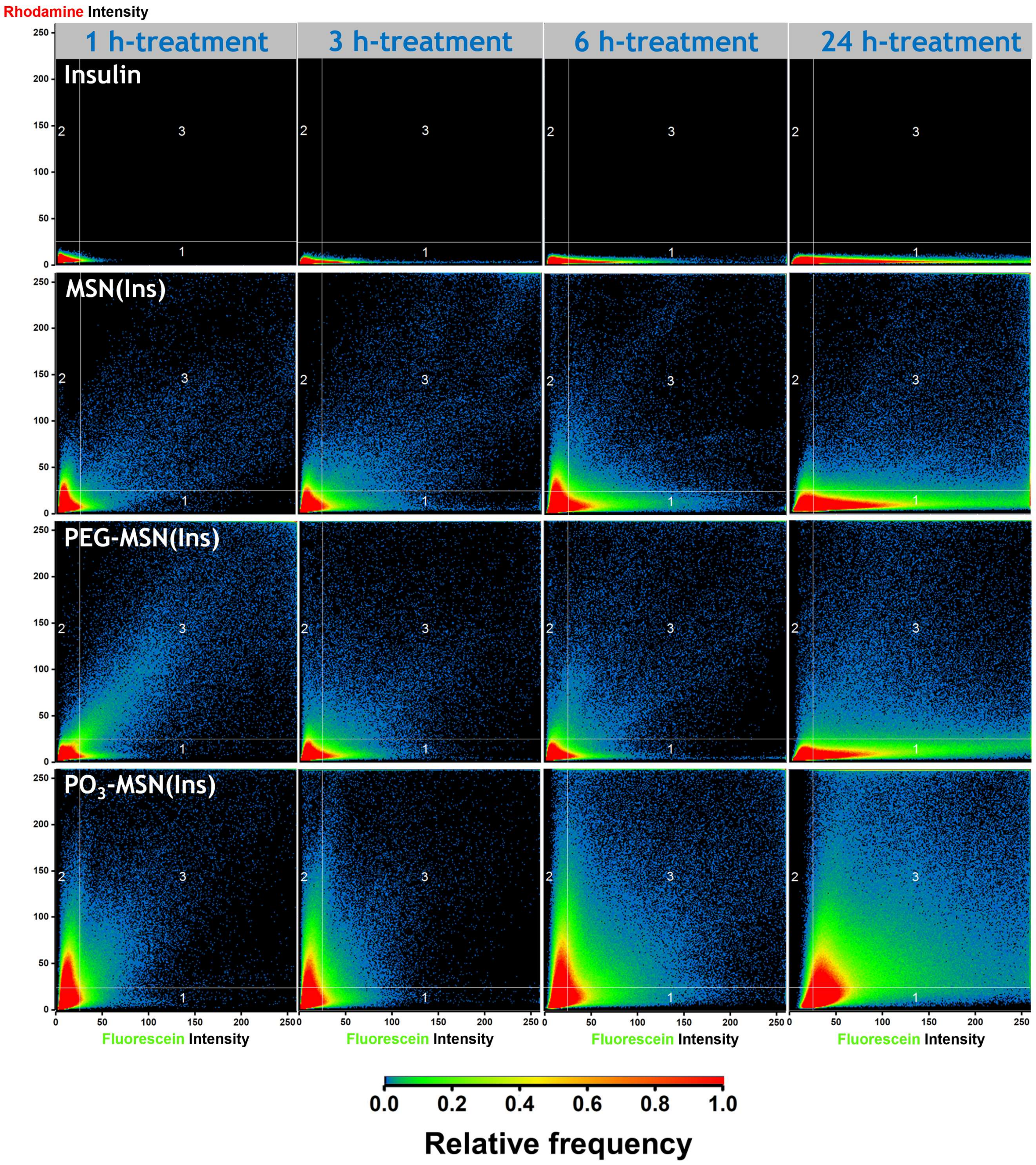

Figure S15. Colocalization graphs obtained from live cell fluorescence imaging. Representative graphs of HCEC-1CT cells treated with non-confined insulin (**Ins^{Flu}**, control) and insulin-loaded particles (**MSN^{Rhod}(Ins^{Flu})**, **PEG-MSN^{Rhod}(Ins^{Flu})**, or **PO3-MSN^{Rhod}(Ins^{Flu})**) after treatment for 1 h, 3 h, 6 h, and 24 h at 37°C, showing the fluorescence of fluorescein (**1:** Ins^{Flu}), rhodamine (**2:** MSN^{Rhod}), and simple spatial overlap of both signals (**3:** Overlap region).

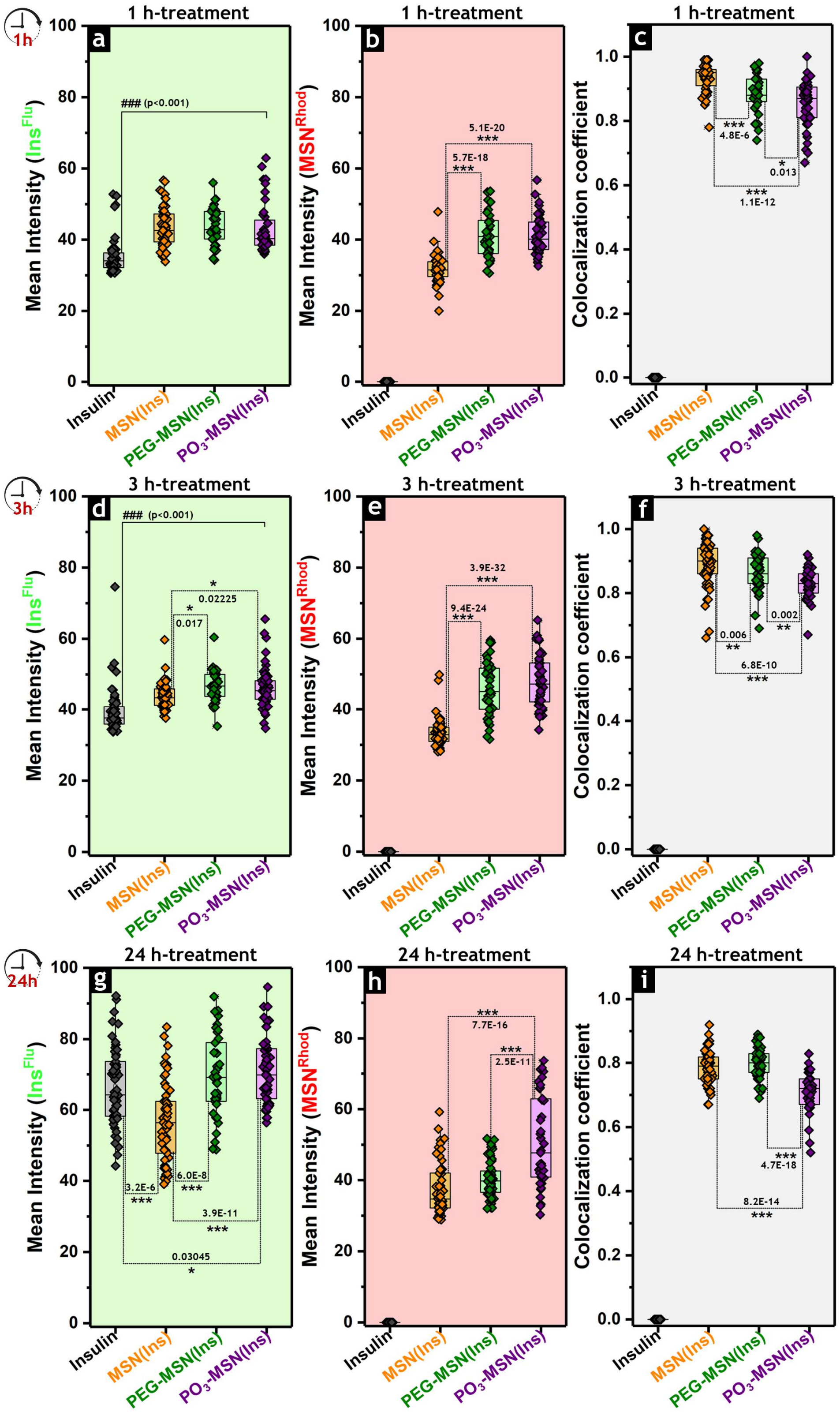

Figure S16. Quantification of particle internalization and intracellular release of insulin from live cell imaging. Quantification of the fluorescence intensities of **Ins^{Flu}** (a, d, g) and rhodamine-labeled MSN (b, e, h), as well as the colocalization coefficient (c, f, i) after 1 h (a-c), 3 h (d-f), and 24 h (g-i) treatments of HCEC-1CT cells with insulin-loaded MSN (**MSN^{Rhod}(Ins^{Flu})**, **PEG-MSN^{Rhod}(Ins^{Flu})**, or **PO$_3$-MSN^{Rhod}(Ins^{Flu})**) or non-confined insulin (**Ins^{Flu}**, control). Data were obtained in three independent cell preparations by quantifying more than 25 regions of interest ($n \geq 25$). One-way ANOVA and Fisher Test expressed significant differences by *($p < 0.05$), **($p < 0.01$) and ***($p < 0.001$).

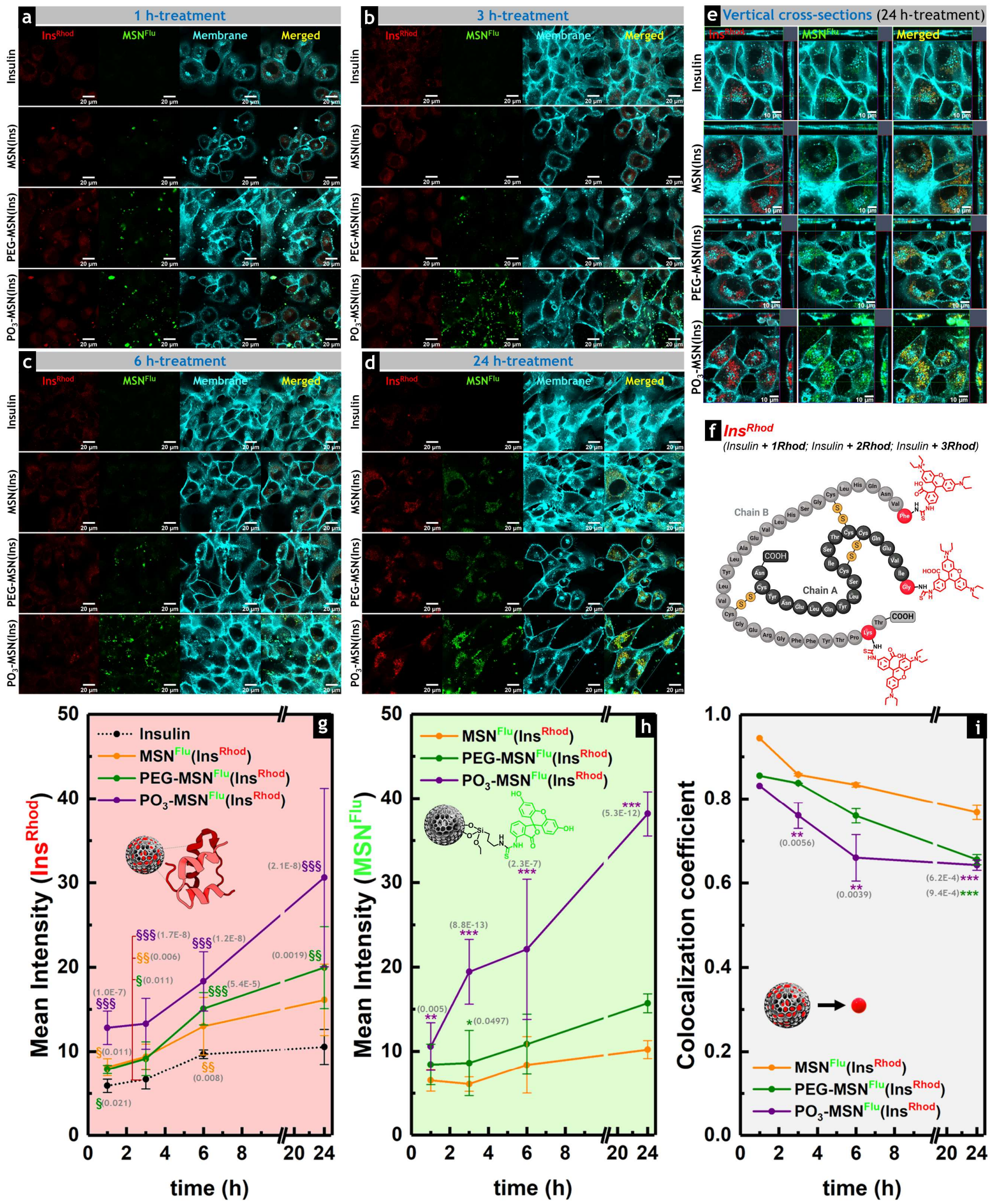

Figure S17. Live fluorescence imaging of HCEC-1CT cells incubated with insulin-loaded MSN (MSN^{Flu}(Ins^{Rhod})). Representative images after incubation for (a) 1 h, (b) 3 h, (c) 6 h, and (d) 24 h at 37°C with insulin-loaded MSN (**MSN^{Flu}(Ins^{Rhod})**, **PEG-MSN^{Flu}(Ins^{Rhod})**, or **PO$_3$-MSN^{Flu}(Ins^{Rhod})**) or rhodamine-labeled insulin (**Ins^{Rhod}**, control). The fluorescence signals from insulin and fluorescein-labeled MSN were simultaneously detected and represented in red (**Ins^{Rhod}**) and green (**MSN^{Flu}**), respectively. The plasma membrane is represented in cyan (CellMask™ Deep Red Plasma Membrane Stain). Scale bars stand for 20 µm. (b) Representative cross-sections of 3D cut stacks obtained from Z-stack imaging after incubation with MSN^{Flu}(Ins^{Rhod}) formulations or **Ins^{Rhod}** for 24 h. Scale bars stand for 10 µm. (f) Schematic representation of the structure of the rhodamine-labeled insulin (**Ins^{Rhod}**) containing up to 3 units of rhodamine per insulin molecule after labeling (created with BioRender.com). Evolution of the fluorescence intensities of (g) **Ins^{Rhod}** and (h) fluorescein-labeled MSN (MSN^{Flu}), as well as (i) the colocalization coefficient over time. Data were obtained in three independent cell preparations by quantifying more than 10 regions of interest ($n \geq 10$) and presented as mean ± standard deviation. Statistically significant differences according to One-way ANOVA and Fisher Test when treatments are compared with respect to the non-confined insulin (**Ins^{Rhod}**, §) or insulin-loaded into calcined MSN (**MSN^{Flu}(Ins^{Rhod})**) are indicated with §/* ($p < 0.05$), §§/** ($p < 0.01$), or §§§/*** ($p < 0.001$).

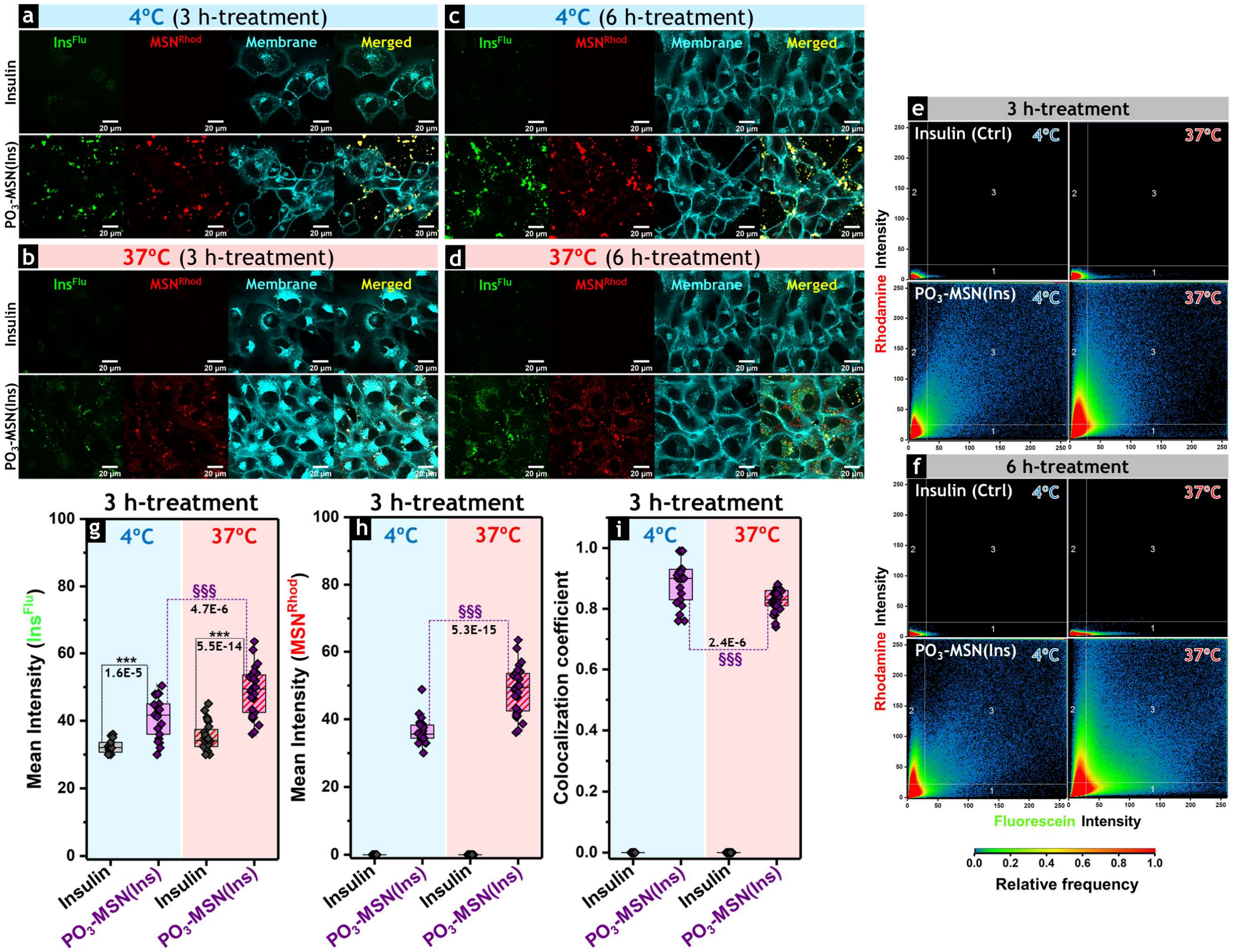

Figure S18. Energy-dependent endocytosis of insulin-loaded MSN. Representative live cell fluorescence images of HCEC-1CT cells incubated with non-confined insulin (**Ins^{Flu}**, control) or insulin loaded on phosphonated MSN (**PO_3-MSN^{Rhod}(Ins^{Flu})**) for 3 h (a, b) and 6 h (c, d), at 4°C (a, c) or 37°C (b, d). The fluorescence signals corresponding to insulin and **PO_3-MSN** were simultaneously detected and represented in green (**Ins^{Flu}**) and red (**PO_3-MSN^{Rhod}**), respectively. The plasma membrane is depicted in cyan (CellMask™ Deep Red Plasma Membrane Stain). Scale bars stand for 20 µm. Representative colocalization graphs showing the fluorescence of fluorescein (**1:** Ins^{Flu}), rhodamine (**2:** MSN^{Rhod}), and spatial overlap of both signals (**3:** Overlap region) after incubation for (e) 3 h or (f) 6 h at 4°C and 37°C. Quantification of the fluorescence intensity of (g) **Ins^{Flu}** and (h) **PO_3-MSN^{Rhod}**, as well as (i) the colocalization coefficient of both signals after 3 h-treatment at 4°C or 37°C. Data were obtained in three independent cell preparations by quantifying more than 21 regions of interest ($n \geq 21$). Statistically significant differences according to One-way ANOVA and Fisher Test when treatments are compared at the same temperature (*) or when the effect of temperature is compared for the same treatment (§) are indicated with ***/§§§ ($p < 0.001$).

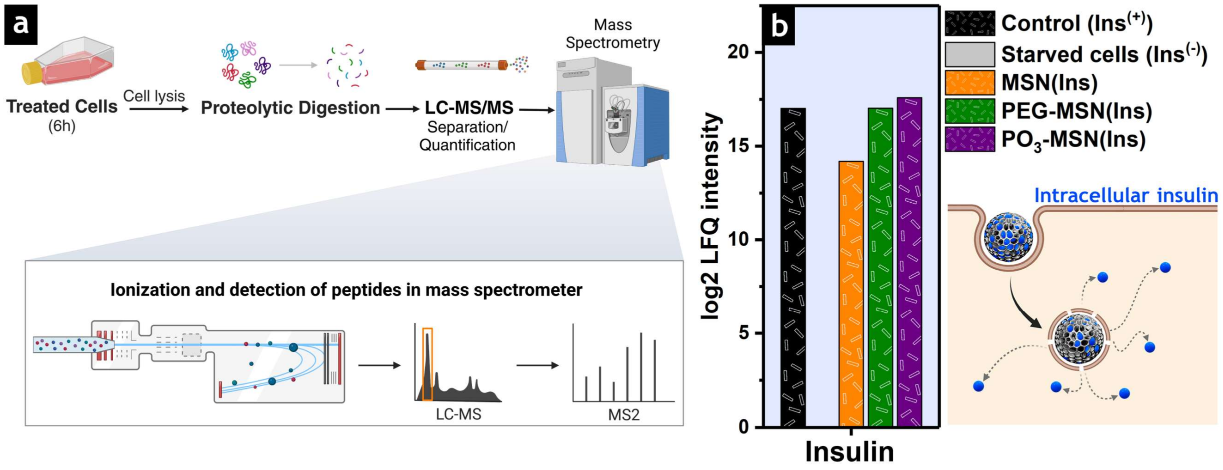

Figure S19. Detection of insulin in cell lysates via untargeted proteomics. (a) Schematic representation of the workflow implemented. (b) log2 LFQ (label-free quantification) intensities corresponding to human insulin detected in HCEC-1CT cells incubated for 6 h with commercial human insulin (control, **Ins$^{(+)}$**, 10 µg·mL^{-1}, 1.7 µM), or with insulin-loaded MSN (**MSN(Ins)**, **PEG- MSN(Ins)**, and **PO$_3$-MSN(Ins)**) dispersed in insulin-free cell culture medium, or in starvation conditions (**Ins$^{(-)}$**). Graphical elements were created with BioRender.com.

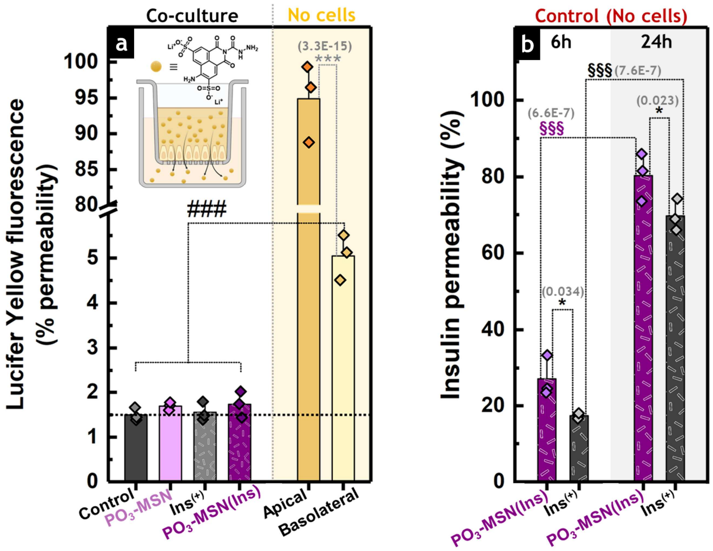

Figure S20. Paracellular permeability of Lucifer Yellow and insulin through Caco-2/HT29-MTX-E12 cells or cell-free inserts. (a) Lucifer Yellow permeability (%), calculated from the fluorescence intensity detected in the basolateral compartment after 24 h-incubation with phosphonated MSN (**PO$_3$-MSN**), non-confined insulin, or insulin-loaded phosphonated particles (**PO$_3$-MSN(Ins)**), followed by 1 h of incubation with Lucifer Yellow in the apical compartment. The dotted line indicates the % permeability of the control (non-treated cells), calculated relative to the total fluorescence of the Lucifer Yellow stock solution (0.1 mg·mL^{-1}). Cell-free permeable inserts (yellow background) showed the maximum Lucifer Yellow permeability through the inserts (apical vs. basolateral compartments), which was significantly higher than the % permeability observed in cell-containing systems (###, one-way ANOVA with Fisher's test, $p < 0.001$). Statistically significant differences between apical and basolateral compartments in these cell-free controls are indicated as *** ($p < 0.001$). (b) Insulin permeability (%) after incubation of non-confined insulin **Ins$^{(+)}$** or **PO$_3$-MSN(Ins)** in the apical compartment of cell-free permeable inserts for 6 h or 24 h, expressed as the relative concentration of insulin detected in the basolateral compartment with respect to the total insulin in the apical stock suspension (100 µg·mL^{-1}, 17 µM). Statistically significant differences were determined by one-way ANOVA and Fisher's test, either between treatments at the same time point (*) or between incubation times for the same treatment (§), and are indicated as */§ $p < 0.05$ and ***/§§§ $p < 0.001$. Data sets are presented as mean ± standard deviation ($N = 3$, biological triplicates).

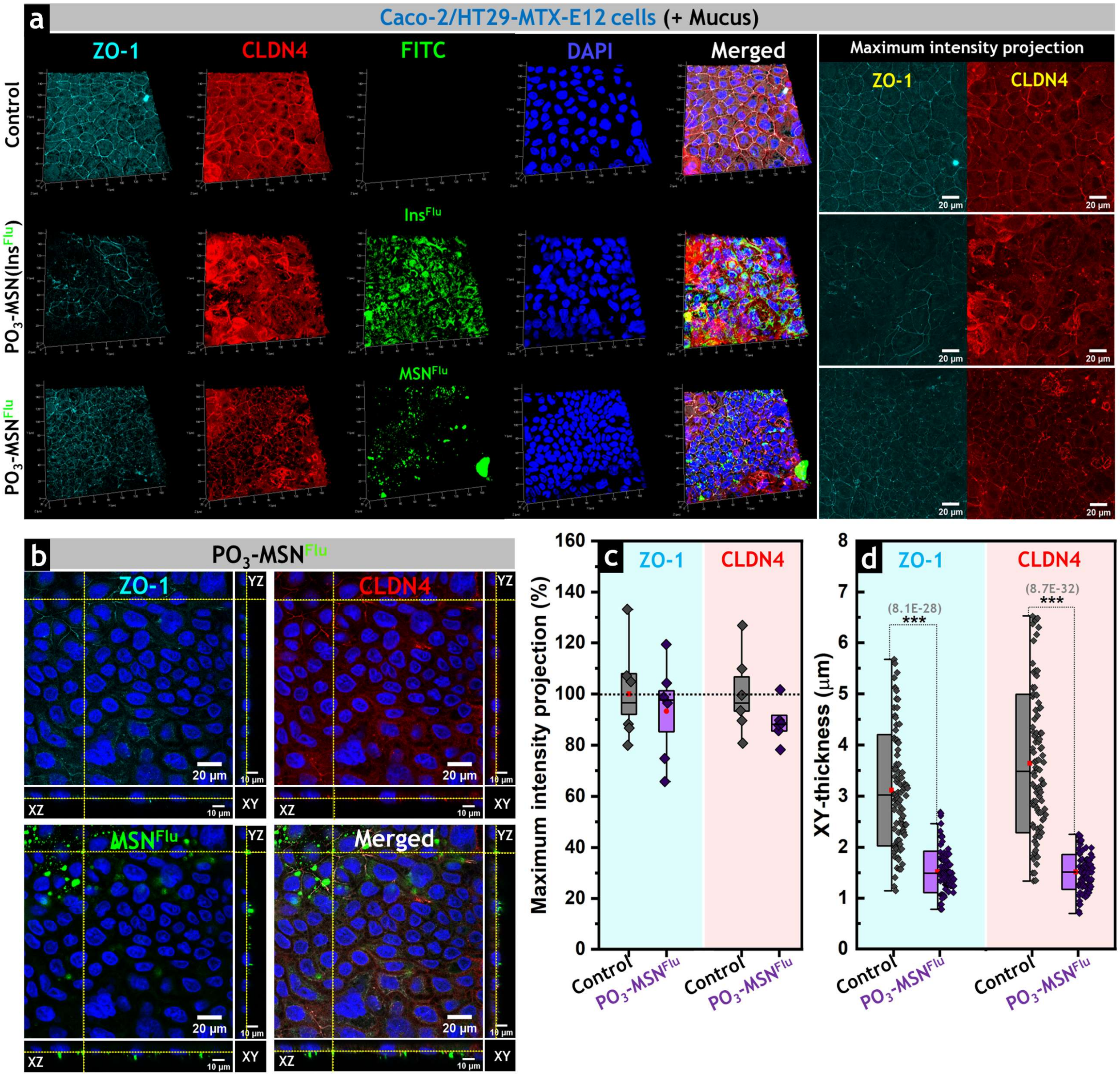

Figure S21. Immunofluorescence staining of tight junction proteins and interaction of PO3-MSN carriers with intestinal cells. Representative 3D reconstructions (63× magnification) showing the distribution of ZO-1 (cyan) and CLDN4 (red), obtained by z-stack imaging of Caco-2/HT29-MTX-E12 cells after 6 h of treatment with fluorescein-labeled insulin loaded into phosphonated MSN (**PO3-MSN(Ins^{Flu})**) or with fluorescein-labeled phosphonated MSN (**PO3-MSN^{Flu}**), both shown in green. The control corresponds to non-treated cells incubated in complete culture medium. Nuclei are stained with DAPI (blue). Scale bars indicate 10 µm in orthogonal views and 20 µm in Z-projection images of ZO-1 and CLDN4. (b) Representative cross-sectional views of 3D reconstructed Z-stack images showing ZO-1 (cyan), CLDN4 (red), and FITC (green) fluorescence signals following treatment with **PO3-MSN^{Flu}**. Nuclei staining (DAPI, blue) served as a reference for selecting the XY projections. Scale bars are 20 µm in XY projections and 10 µm in XZ and YZ projections. (c) Quantification of mean fluorescence intensity of ZO-1 and CLDN4 of maximum intensity projection images obtained from 3D reconstructions ($n = 6$ images derived from independent biological triplicates, including technical duplicates). (d) Quantification of XY-thickness (d, µm) of ZO-1 and CLDN4, based on the analysis of more than 90 cells ($n \geq 90$) from three independent preparations (biological triplicates including technical duplicates). Data sets are presented as box plots, with the mean (red dot) and standard deviation shown. Statistically significant differences, determined by one-way ANOVA and Fisher's test, between **PO3-MSN^{Flu}**-treated and control (non-treated) cells are indicated as *** $p < 0.001$.

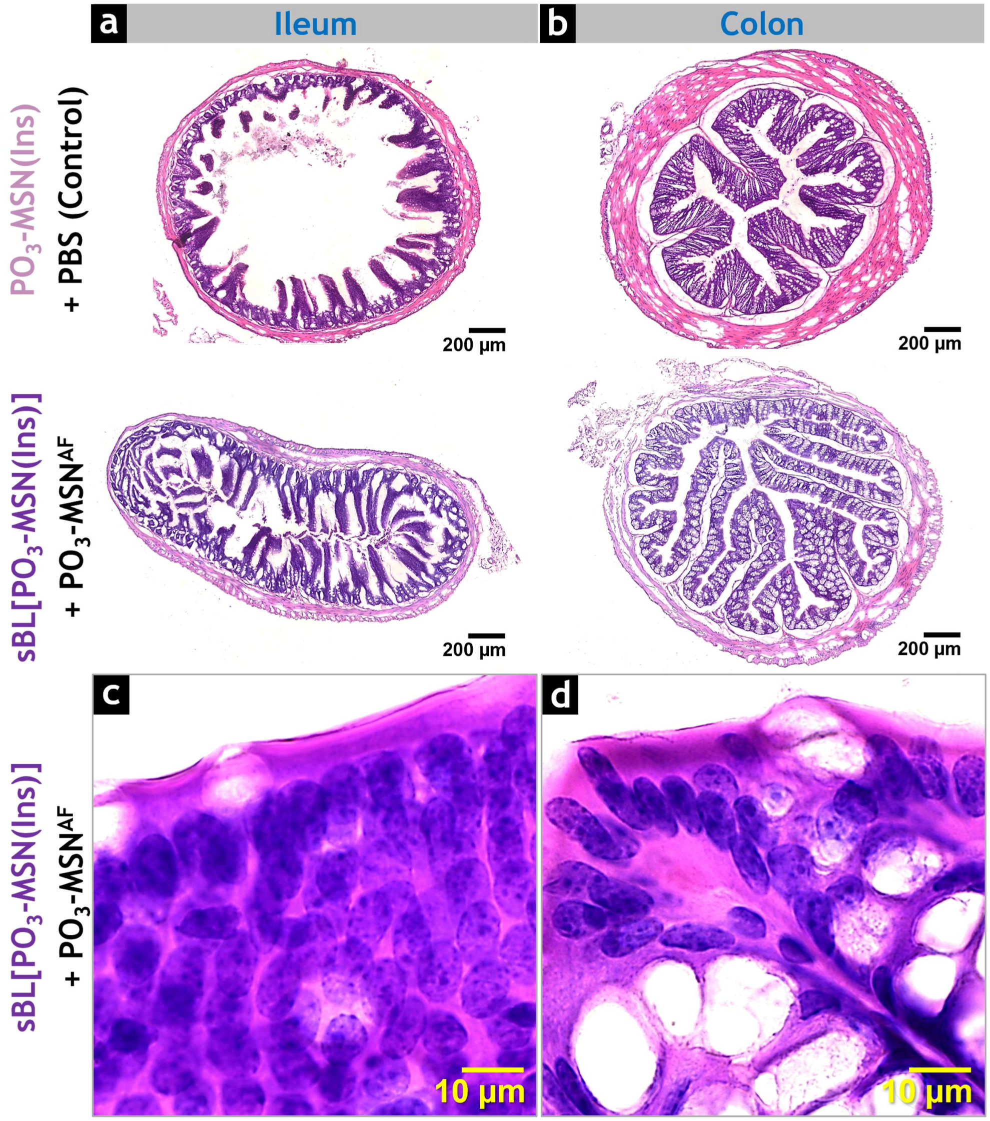

Figure S22. Histopathological characterization of intestinal tissue from mice gavaged with MSN(Ins)-based formulations. Representative images of (a) ileum and (b) colon tissue sections stained with hematoxylin and eosin (H&E) from mice that received insulin **PO_3-MSN(Ins)**-suspension in PBS (control) or **sBL[PO_3-MSN(Ins)]** capsules followed by an additional oral gavage of **PO3-MSN^{AF}** suspension in PBS. Cell nuclei were counterstained with DAPI (10 µg·mL^{-1}). Representative images of tissue samples from the treated group (**sBL[PO_3-MSN(Ins)]**-capsules) obtained at a higher magnification showed no pathological alterations in either (c) ileum or (d) colon, suggesting that the **PO_3-MSN** particles do not compromise epithelial cell integrity.

REFERENCES

(1) Juère, E.; Caillard, R.; Marko, D.; Del Favero, G.; Kleitz, F. Smart Protein-Based Formulation of Dendritic Mesoporous Silica Nanoparticles: Toward Oral Delivery of Insulin. *Chem. - A Eur. J.* **2020**, *26* (23), 5195–5199. https://doi.org/10.1002/chem.202000773.

(2) Kremsmayr, T.; Aljnabi, A.; Blanco-Canosa, J. B.; Tran, H. N. T.; Emidio, N. B.; Muttenthaler, M. On the Utility of Chemical Strategies to Improve Peptide Gut Stability. *J. Med. Chem.* **2022**, *65* (8), 6191–6206. https://doi.org/10.1021/acs.jmedchem.2c00094.

(3) Bergen, J.; Karasova, M.; Bileck, A.; Pignitter, M.; Marko, D.; Gerner, C.; Del Favero, G. Exposure to Dietary Fatty Acids Oleic and Palmitic Acid Alters Structure and Mechanotransduction of Intestinal Cells in Vitro. *Arch. Toxicol.* **2023**, *97*, 1659–1675. https://doi.org/10.1007/s00204-023-03495-3.

(4) Peskin, A. V.; Winterbourn, C. C. Assay of Superoxide Dismutase Activity in a Plate Assay Using WST-1. *Free Radic. Biol. Med.* **2017**, *103* (December 2016), 188–191. https://doi.org/10.1016/j.freeradbiomed.2016.12.033.

(5) Jobst, M.; Kiss, E.; Gerner, C.; Marko, D.; Del Favero, G. Activation of Autophagy Triggers Mitochondrial Loss and Changes Acetylation Profile Relevant for Mechanotransduction in Bladder Cancer Cells. *Arch. Toxicol.* **2023**, *97* (1), 217–233. https://doi.org/10.1007/s00204-022-03375-2.

(6) Del Favero, G.; Zeugswetter, M.; Kiss, E.; Marko, D. Endoplasmic Reticulum Adaptation and Autophagic Competence Shape Response to Fluid Shear Stress in T24 Bladder Cancer Cells. *Front. Pharmacol.* **2021**, *12* (May), 647350. https://doi.org/10.3389/fphar.2021.647350.

(7) Hohagen, M.; Guggenberger, P.; Kiss, E.; Kählig, H.; Marko, D.; Del Favero, G.; Kleitz, F. TANNylation of Mesoporous Silica Nanoparticles and Bioactivity Profiling in Intestinal Cells. *J. Colloid Interface Sci.* **2022**, *623*, 962–973. https://doi.org/10.1016/j.jcis.2022.05.035.

(8) Iriarte-Mesa, C.; Jobst, M.; Bergen, J.; Kiss, E.; Ryoo, R.; Kim, J. C.; Crudo, F.; Marko, D.; Kleitz, F.; Del Favero, G. Morphology-Dependent Interaction of Silica Nanoparticles with Intestinal Cells: Connecting Shape to Barrier Function. *Nano Lett.* **2023**, *23* (16), 7758−7766. https://doi.org/10.1021/acs.nanolett.3c00835.

(9) Shah, D.; Guo, Y.; Ocando, J.; Shao, J. FITC Labeling of Human Insulin and Transport of FITC-Insulin Conjugates through MDCK Cell Monolayer. *J. Pharm. Anal.* **2019**, *9* (6), 400–405. https://doi.org/10.1016/j.jpha.2019.08.002.

(10) Zougman, A.; Selby, P. J.; Banks, R. E. Suspension Trapping (STrap) Sample Preparation Method for Bottom-up Proteomics Analysis. *Proteomics* **2014**, *14* (9), 1006–1000. https://doi.org/10.1002/pmic.201300553.

(11) Perez-Riverol, Y.; Csordas, A.; Bai, J.; Bernal-llinares, M.; Hewapathirana, S.; Kundu, D. J.; Inuganti, A.; Griss, J.; Mayer, G.; Eisenacher, M.; Enrique, P.; Uszkoreit, J.; Pfeuffer, J.; Sachsenberg, T.; Yılmaz, S.; Tiwary, S.; Cox, J.; Audain, E.; Walzer, M.; Jarnuczak, A. F.; Ternent, T.; Brazma, A.; Vizcaino, J. A. The PRIDE Database and Related Tools and Resources in 2019 : Improving Support for Quantification Data. *Nucleic Acids Res.* **2019**, *47* (November 2018), 442–450. https://doi.org/10.1093/nar/gky1106.

(12) Iriarte-Mesa, C.; Bergen, J.; Danielyan, K.; Crudo, F.; Marko, D.; Kählig, H.; Del Favero, G.; Kleitz, F. Functionalization of Silica Nanoparticles for Tailored Interactions with Intestinal Cells and Chemical Modulation of Paracellular Permeability. *Small Sci.* **2025**, *5*, 2400112. https://doi.org/10.1002/smsc.202400112.

(13) Rwigemera, A.; Mamelona, J.; Martin, L. J. Inhibitory Effects of Fucoxanthinol on the Viability of Human Breast Cancer Cell Lines MCF-7 and MDA-MB-231 Are Correlated with Modulation of the NF-KappaB Pathway. *Cell Biol. Toxicol.* **2014**, *30* (3), 157–167. https://doi.org/10.1007/s10565-014-9277-2.

(14) Del Favero, G.; Bergen, J.; Palm, L.; Fellinger, C.; Matlaeva, M.; Szabadi, A.; Fernandes, A. S.; Saraiva, N.; Schröder, C.; Marko, D. Short-Term Exposure to Foodborne Xenoestrogens Affects Breast Cancer Cell Morphology and Motility Relevant for Metastatic Behavior In Vitro. *Chem. Res. Toxicol.* **2024**, *37* (10), 1634–1650.

https://doi.org/10.1021/acs.chemrestox.4c00061.

(15) Lim, C. Y.; Owens, N. A.; Wampler, R. D.; Ying, Y.; Granger, J. H.; Porter, M. D.; Takahashi, M.; Shimazu, K. Succinimidyl Ester Surface Chemistry: Implications of the Competition between Aminolysis and Hydrolysis on Covalent Protein Immobilization. *Langmuir* **2014**, *30* (43), 12868–12878. https://doi.org/10.1021/la503439g.

(16) Brunauer, S.; Emmett, P. H.; Teller, E. Adsorption of Gases in Multimolecular Layers. *J. Am.Chem.Soc.* **1938**, *60* (2), 309–319. https://doi.org/10.1021/ja01269a023.

(17) Greenfield, N. J. Using Circular Dichroism Spectra to Estimate Protein Secondary Structure. *Nat. Protoc.* **2007**, *1* (6), 2876–2890. https://doi.org/10.1038/nprot.2006.202.

(18) Amaral, M.; Martins, A. S.; Catarino, J.; Faísca, P.; Kumar, P.; Pinto, J. F.; Pinto, R.; Correia, I.; Ascensão, L.; Afonso, R. A.; Gaspar, M. M.; Charmier, A. J.; Figueiredo, I. V.; Reis, C. P. How Can Biomolecules Improve Mucoadhesion of Oral Insulin? A Comprehensive Insight Using Ex-Vivo, in Silico, and in Vivo Models. *Biomolecules* **2020**, *10* (5), 675. https://doi.org/10.3390/biom10050675.

(19) Zhang, Z.; Marshall, A. G. A Universal Algorithm for Fast and Automated Charge State Deconvolution of Electrospray Mass-to-Charge Ratio Spectra. *J. Am. Soc. Mass Spectrom.* **1998**, *9* (3), 225–233. https://doi.org/10.1016/S1044-0305(97)00284-5.

(20) Maria Chong, A. S.; Zhao, X. S. Functionalization of SBA-15 with APTES and Characterization of Functionalized Materials. *J. Phys. Chem. B* **2003**, *107* (46), 12650–12657. https://doi.org/10.1021/jp035877+.

(21) Protsak, I. S.; Morozov, Y. M.; Dong, W.; Le, Z.; Zhang, D.; Henderson, I. M. A 29Si, 1H, and 13C Solid-State NMR Study on the Surface Species of Various Depolymerized Organosiloxanes at Silica Surface. *Nanoscale Res. Lett.* **2019**, *14*, 160. https://doi.org/10.1186/s11671-019-2982-2.

(22) Ateia, E. E.; Saeid, Y. A.; Abdelmaksoud, M. K. Synthesis and Characterization of CaFe1.925Gd0.025Sm0.05O4/PEG Core–Shell Nanoparticles for Diverse Applications. *J. Supercond. Nov. Magn.* **2023**, *36* (3), 1011–1024. https://doi.org/10.1007/s10948-023-06535-2.

(23) Widjonarko, D. M.; Jumina, J.; Kartini, I.; Nuryono, N. Phosphonate Modified Silica for Adsorption of Co(II), Ni(II), Cu(II), and Zn(II). *Indones. J. Chem.* **2014**, *14* (2), 143–151. https://doi.org/10.22146/ijc.21251.

(24) Singh, A.; Guleria, A.; Neogy, S.; Rath, M. C. UV Induced Synthesis of Starch Capped CdSe Quantum Dots: Functionalization with Thiourea and Application in Sensing Heavy Metals Ions in Aqueous Solution. *Arab. J. Chem.* **2020**, *13* (1), 3149–3158. https://doi.org/10.1016/j.arabjc.2018.09.006.

(25) Das, S.; Manam, J. Fluorescein Isothiocyanate and Rhodamine B Dye Encapsulated Mesoporous SiO2 for Applications of Blue LED Excited White LED. *Opt. Mater. (Amst).* **2018**, *79* (March), 259–263. https://doi.org/10.1016/j.optmat.2018.03.052.

(26) Siepmann, J.; Peppas, N. A. Modeling of Drug Release from Delivery Systems Based on Hydroxypropyl Methylcellulose (HPMC). *Adv. Drug Deliv. Rev.* **2012**, *64*, 163–174. https://doi.org/10.1016/j.addr.2012.09.028.

(27) Costa, P.; Lobo, J. M. S. Modeling and Comparison of Dissolution Profiles. *Eur. J. Pharm. Sci.* **2001**, *13*, 123–133. https://doi.org/10.1016/S0928-0987(01)00095-1.

(28) Higuchi, T. Mechanism of Sustained- Action Medication. Theoretical Analysis of Rate of Release of Solid Drugs Dispersed in Solid Matrices. *J. Pharm. Sci.* **1963**, *52* (12), 1145–1149. https://doi.org/10.1002/jps.2600521210.

(29) Korsmeyer, R. W.; Gumy, R.; Doelker, E.; Buri, P.; Peppas, N. A. Mechanisms of Solute Release from Porous Hydrophilic Polymers. *Int. J. Pharm.* **1983**, *15*, 25–35. https://doi.org/10.1016/0378-5173(83)90064-9.
